# Biomedical titanium-tantalum alloys for orthopedic implant applications: From manufacturing to biological performance

**DOI:** 10.1016/j.bioactmat.2026.02.038

**Published:** 2026-03-02

**Authors:** S.D. Luo, S. Paisansuthichol, N. Tran, M. Qian, T. Song

**Affiliations:** a Centre for Additive Manufacturing, School of Engineering, RMIT University, Melbourne, VIC, 3000, Australia; b School of Science, RMIT University, Melbourne, VIC 3000, Australia

**Keywords:** Ti-Ta alloy, Biomaterial, Additive manufacturing, Lattice structure, Biological response

## Abstract

An ideal biomedical material for orthopedic implant applications is characterized by being biologically compatible with the human body, mechanically matching human cortical or cancellous bones, and releasing no cytotoxic ions over its servicing time. Titanium-tantalum (Ti-Ta) binary alloy is such a potential biomaterial that is composed of the most biocompatible metal elements, and both metals have been heavily used as implant materials in clinical surgeries for decades. Additive manufacturing (AM) enables the innovative design of patient-specific medical implants, e.g., Ti-Ta lattices which display unique mechanical and biological properties. Such Ti-Ta lattice implants can best mimic the mechanical properties of natural bones and offer open pores and pore networks to promote implant-bone biological interactions, which are essential to successful and long-term implantation. This article extensively reviews the biomedical Ti-Ta alloys. It starts with a brief overview of Ti-Ta alloy development, then elaborates on its manufacturing, the resulting microstructures pertinent to cooling conditions, mechanical properties, and surface modification to improve the bioactivity. Ti-Ta lattices from AM process are particularly highlighted as they enable elastic moduli close to those of natural bones. Next, the corrosion resistance and wettability of Ti-Ta alloys are addressed. Last, the biological responses of Ti-Ta alloy *in vitro* and *in vivo* are scrutinized and discussed, highlighting the significance of the biocompatible Ti-Ta chemistry, the surface features and the AM-enabled lattice structures. This paper aims to establish a comprehensive and systematic understanding of binary Ti-Ta alloys for biomaterials research and artificial bone implant applications.

## Highlights

• An exhaustive review of the manufacturing, microstructure and mechanical properties of Ti-Ta alloys.
• A comprehensive mechanical comparison of AM Ti-Ta lattices with human bone.
• An in-depth critical evaluation of the biological responses of Ti-Ta alloys.
• A clear identification of opportunities for AM Ti-Ta lattices with or without surface treatments as bone implants.

## Graphical abstract

**Figure:**
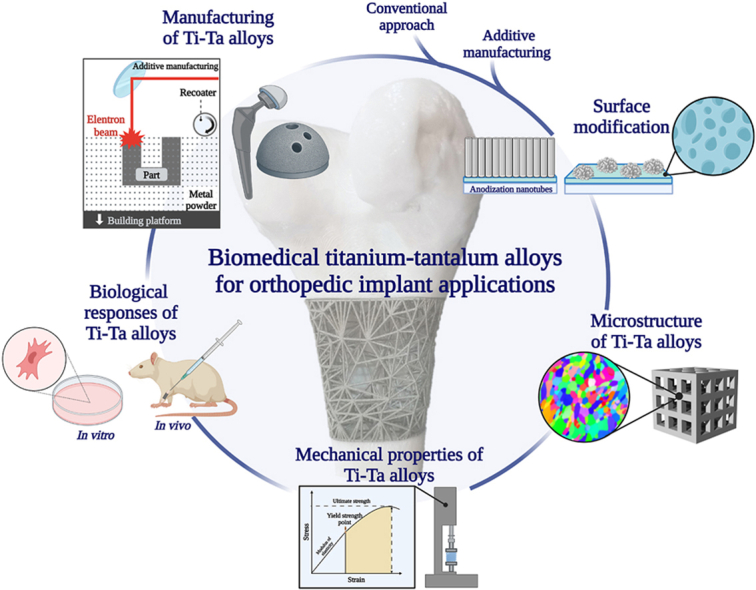

## 1 Introduction

Global population and life expectancy increases entail a growing demand for artificial bone implants. They replace age-related degenerated bones or repair bone defects caused by trauma, tumor resection, revision arthroplasty, etc. Take Australia as an example, approximately one in every 170 individuals underwent primary or revision hip/knee/shoulder joint replacement surgeries in 2023, with a total of 146,795 cases and a 20% increase from 2018, reported by the Australian Orthopaedic Association [1]. Ceramics, polymers, and metals are common material systems used to meet the diverse orthopedic demands, each with its merits and deficiencies. Bioinert ceramics, such as hydroxyapatite, alumina, and zirconia, excel in hardness, wear, and corrosion resistance but are deficient in ductility, stiffness, and flexibility, making them suitable for joint replacements and bone coating, among other applications [2]. Polymers such as polyethylene, polylactic acid, and polyaryletherketone are advantageous in terms of flexibility, biodegradability, density, and cost; however, their mechanical strength and tribological properties are inadequate, which restricts their applications to fracture fixation, bone cement, and drug delivery, among others [3]. Metals have exceptional mechanical strength, ductility and durability, which are essential for load-bearing bone repairs; however, they face disadvantages such as a high elastic modulus, insufficient resistance to corrosion and wear, and the resulting release of harmful ions and debris [4,5]. To pursue improved biosafety, bifunctionality and longevity, a range of metallic biomaterials has been investigated, under development or commercialized for increasing orthopedic applications [[6], [7], [8], [9], [10], [11], [12]], summarized in Table 1 [6,7,10,[13], [14], [15], [16], [17], [18], [19]].

**Table 1:** Physical and mechanical properties of metallic biomaterials (in a solid form). Properties of human natural bones are included.

Materials	Density (g/cm^3^)	Cytotoxic element	Young's modulus (GPa)	Tensile strength (MPa)	Strain to fracture (%)	Remarks	Ref.
SS 316L	7.9-8.1	Fe, Cr, Ni	189-205	490-1731	12-52	Mostly used as short-term implants and screws	[6,7]
Co-Cr	8.27-9.20	Co, Cr	220-250	600-2280	8-28	High hardness and wear	[6,7]
Au	19.3	None	80	130-200	4-50	Dental implants, least affordable	[7]
Mg alloy	1.74-2.00	Al, RE	40-60	150-380	2-40	Biodegradable, unsuitable for load-bearing areas	[6,10]
Pure Ti	4.51	None	105	240-550	15-24	High aspect strength but low hardness and wear	[7]
Pure Ta	16.69	None	186-191	205-480	1-30	Most biocompatible but high-density and modulus	[7]
Ti-6Al-4V	4.20-4.50	Al, V	110-114	895-930	6-10	Widely used for statically loading areas	[6,7]
Ti-Zr	4.5-4.9	None	88-110	400-1025	13-18	Biocompatible and potential for dental application	[15]
Ti-6Al-7Nb	4.5	Al	114	900-1050	8-15	Temporary fixation devices	[19]
Ti-Nb-Ta-Zr	4.5-5.2	None	32-80	400-1180	13-19	Biocompatible and low modulus β-Ti alloys	[19]
Ti-Nb	4.87-5.76	None	50-99	420-920	4.6-12.6	Biocompatible and corrosion resistance	[17,18]
Ti-Mo	4.7-5.1	None	65-93	731-1069	10-47	Orthopedic and dental applications	[17]
Ti-Ta	4.8-10.8	None	67-100	500-700	10-25	Well biocompatible and relatively low modulus	[13]
Cortical bone	1.72-2.10	–b	13.6-35.3	68-213	1.07-4.0	Vary with human gender and age	[7,14]
Cancellous bone^a^	0.19-0.50	–	0.02-0.83	0.88-10.6	2.05-8.50	Vary with human gender and age	[14]

a Compressive properties.

b –: Not applicable.

Biosafety is the utmost criterion for the development of orthopedic metals, which requires candidate metals to be nontoxic and inert in the human body, thus posing no harm to the patient. This means that the candidate metals must be corrosion-resistant in a physiological environment, or their corrosion products must induce no cytotoxic reactions, adverse inflammatory, or immune responses. This criterion restricts new metallic materials to the limited non-cytotoxic metals – Ti, Ta, Zr, Nb, Mo, Mg, Zn, etc [9,19]. Apart from biosafety, the mechanical properties of the candidate biomaterial are vital for a successful long-term service after implantation in load-bearing areas. Elastic modulus is particularly pivotal because a mismatch in stiffness between the implant and host bone will elicit stress shielding, which leads to implant loosening, bone resorption and eventual implant failure. The elastic modulus varies from 13.6 GPa to 35.3 GPa in human cortical bones or from 0.02 GPa to 0.83 GPa in cancellous bones, subject to gender and age [14,20]. In contrast, commercially available metallic biomaterials exhibit a significantly higher elasticity, often exceeding 100 GPa, as shown in Table 1. Although introducing porosity to these metals/alloys can substantially reduce their effective elastic modulus [21], developing new biomaterials with an intrinsically low elastic modulus is still a top priority [22]. In addition to elasticity, some existing biomaterials release metal ions over time, such as V, Ni, Fe, Co, and Al, which have been proven toxic to cells/humans [[23], [24], [25], [26]]. Among the non-cytotoxic metals above, pure Ta is the most biosafe metal and has been surgically used as devices and implants in biomedical engineering since the 1940's [19,27,28]. However, Ta is a dense refractory metal with a density of 16.69 g/cm^3^ and a melting point of 2996 °C, which severely constrains its manufacturability. Thus, the idea of alloying Ta with these non-cytotoxic metals has naturally been sparked. Titanium, another long-practiced bio-metal [27,28], is an ideal choice because Ti is the second most biocompatible metal. It possesses a relatively low density (4.51 g/cm^3^) and melting point (1660 °C) among these metals, alongside a high strength-to-weight ratio (≥53 kN m/kg). Developing biomaterials from binary Ti-Ta alloys has attracted great interest in this regard. Compared to the existing metallic biomaterials or those under development, Ti-Ta alloys exhibit advantages as follows:

• Consisting of acclaimed Ta and Ti metals, both of which have been surgically practiced for decades.
• Highly corrosion resistant to human body fluids due to the natural formation of a passive thin oxide layer.
• Low modulus relative to the existing commercial implant metals.

Interestingly, Ti-Ta alloy was not initially intended for biomedical applications. Kroll published the first available publication on Ti-Ta alloy in 1937 [29]. He fabricated Ti-20Ta (in wt.%, unless specified otherwise) by powder metallurgy (PM) process and investigated its rollability and hardness [29]. Subsequently, Kroll prepared Ti-Ta alloys containing up to 40%Ta by vacuum arc melting and found all compositions malleable [30]. In the following decades, Ti-Ta alloys were intensively investigated, addressing the main aspects: i) the composition-microstructure relationship aiming to establish the equilibrium phase diagram [31,32]; ii) metastable phases and their transformation as a function of alloy composition and cooling condition [[33], [34], [35], [36], [37]]; iii) mechanical properties with respect to the constituent phases [35,38]; and iv) corrosion and oxidation characteristics for the potential use in nuclear reactors to contain molten plutonium [39,40].

In the late 1980s, Ti-Ta alloys started being perceived as a biomaterial. In 1989, Breme et al. [41] and Gibbesch et al. [42] explored Ti-Ta alloys as possible dental implants. They examined their corrosion behavior in a 0.9% saline solution and the microstructure and mechanical properties of Ti-Ta/alumina joints. In 1998, Prigent et al. [43] assessed the biocompatibility of Ti-5Ta for oral implantology and compared it with pure Ti, copper, and plastic. Since 2000, Ti-Ta alloys have attracted increasing interest with research efforts progressively focusing on: i) biomechanical properties, especially elasticity [13]; ii) corrosion resistance in simulated body fluid (SBF) [44]; iii) *in vitro* and *in vivo* biological properties [45,46]. Enabled by additive manufacturing (AM), the design and fabrication of Ti-Ta lattices has emerged as a hotspot since 2018 [47]. Various lattices have been fabricated using powder bed fusion techniques and comprehensively evaluated as candidate biomaterials for mimicking human bone tissue.

Despite a long research history and fruitful outputs, to the best of our knowledge, there is a lack of a comprehensive review and an insightful understanding of Ti-Ta alloys, particularly for orthopedic implant applications. Most recently, a review article was published, which briefly overviewed the major aspects of Ti-Ta alloys, but without discussion of their biological merits [48]. A few review articles covered Ti-Ta alloys only as part of the review addressing the issues pertinent to the article topics. For example, Weng et al. [17] provided a short discussion of Ti-Ta mechanical properties with respect to phases; Qian et al. [49] briefly summarised AM techniques that have been applied to Ti-Ta fabrication; Mohsan et al. [50] reviewed the fabrication of Ti-Ta alloys via laser powder bed fusion process and the resultant microstructure and mechanical properties; Bandyopadhyay et al. [9] briefly discussed the biological response of Ti-Ta alloys. In this work, we aim to provide an updated and comprehensive review of Ti-Ta alloys, summarizing and discussing their conventional and AM fabrication processes, microstructural features, mechanical properties, surface modification techniques, corrosion resistance and particularly *in vitro* and *in vivo* biological properties. Research data of key properties are exhaustively documented from existing literature, with a view to serving a solid database for biomaterial development. The scope of this article is limited to binary Ti-Ta alloys, excluding Ti-Ta based multi-element materials. This review aims to advance the understanding of Ti-Ta alloys as promising candidates for orthopedic implants and guide future research in material design, surface engineering and clinical translation.

## 2 Manufacturing of Ti-Ta alloys

**Fig. 1:**
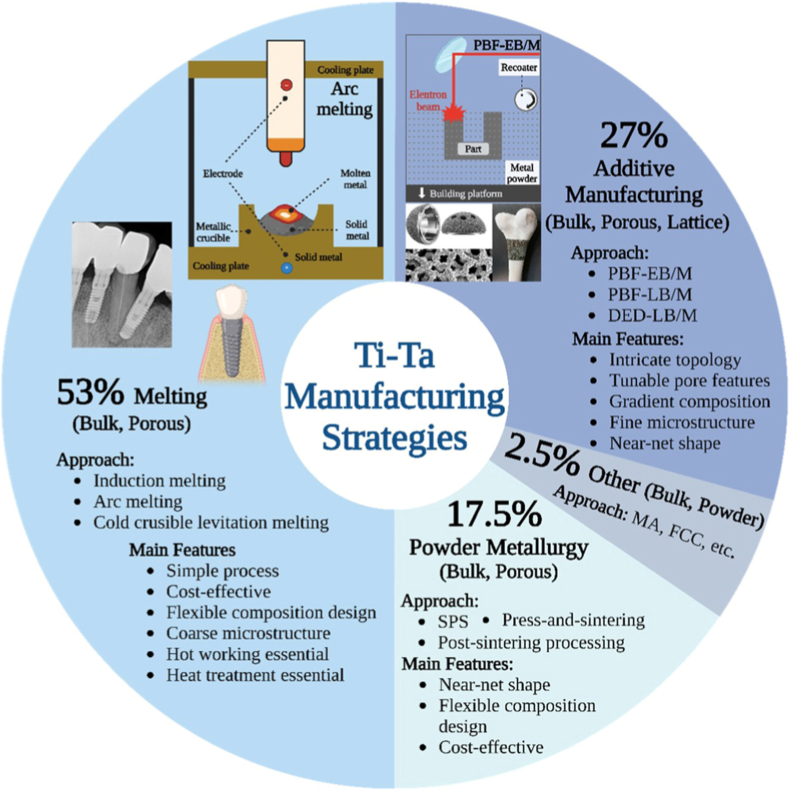
Classification of manufacturing strategies that have been applied to Ti-Ta alloy fabrication covered in this review. SPS: spark plasma sintering, MA: mechanical alloying, FFC: Fray-Farthing-Chen process, PBF-EB/M: powder bed fusion-electron beam, PBF-LB/M: powder bed fusion-laser beam, DED-LB/M: laser directed energy deposition. Images are adapted from Refs. [[51], [52], [53]].

Ti-Ta alloys have been mainly prepared from elemental Ti and Ta through melting, PM, AM, etc. Fig. 1 summarizes the manufacturing strategies applied to Ti-Ta alloy fabrication, outlining the specific approach, product form (bulk, porous, lattice, etc) and main features of the strategy. The numerical percentage indicated refers to the approximate percentage of the corresponding strategy utilized in Ti-Ta alloy studies. Two metals are so distinct in the melting points (1660 °C vs. 2996 °C) and densities (4.501 g/cm^3^ vs. 16.6 g/cm^3^) that producing a homogeneous composition and microstructure essentially remains unlikely without extensive remelting or any prolonged homogenization treatment.

### 2.1 Conventional approach – melting

Melting has long been mostly (53% as indicated in Fig. 1) used to fabricate Ti-Ta alloys. Metal sponges/chips/sheets in designed proportions are melted under a high-purity argon atmosphere or vacuum in an arc or induction heating furnace, then cast into a cold crucible to make an alloy ingot. This approach, partly due to simplicity, is prevailing but produces notable compositional inhomogeneity [35,54,55]. To remove or minimize the inhomogeneity, repetitious melting up to ten times and/or a lengthy homogenization treatment has often been practiced [13,33,34,54,55]. Thus, the produced Ti-Ta alloys exhibit coarse microstructures, which makes hot working, such as rolling and swaging, necessary to refine the microstructure, followed by heat treatment to tailor the microstructure and mechanical properties.

### 2.2 Conventional approach – powder metallurgy

PM is another conventional process widely utilized to prepare Ti-Ta alloys [[56], [57], [58], [59]]. In a typical PM route, elemental Ti and Ta powder mixes are cold compacted and isothermally sintered in vacuum or high-purity argon; during sintering, Ti and Ta powder particles bond via mutual diffusion, forming Ti-Ta solid solution [60,61]. Other PM routes, like cold spray combined with subsequent heat treatment, have also been employed to fabricate Ti-Ta alloys [62,63]. Tantalum is a slow diffuser compared to other β-stabilizing elements in Ti alloys. For example, its interdiffusion coefficient at 1200 °C is approximately 3.3 × 10^−10^ cm^2^/s in Ti-20at.%Ta and 1.53 × 10^−11^ cm^2^/s in Ti-40at.%Ta, respectively, one and two orders of magnitude lower than that of V in Ti-20at.%V and Ti-40at.%V at the same temperature [64]. Slow diffusion usually brings in two deficiencies in PM Ti-Ta alloys, i.e., insufficient densification and inhomogeneous microstructure [65]. Liu et al. [60] observed those two deficiencies in as-sintered Ti-(48.6-79.1)Ta. In their work, the compacts of fine Ti (<45 μm) and Ta (<5 μm) powders were isothermally held for 2 h at 1200-1500 °C in a vacuum of 10^−4^ Pa; a relative density below 98.5% was achieved; meanwhile, the alloys exhibited obviously Ti-enriched and Ta-enriched zones in the sintered microstructure. The formation of Kirkendall pores due to the unbalanced interdiffusion between Ti and Ta imposes another concern for achieving fully dense Ti-Ta alloys via the conventional press-and-sinter PM approach [66].

To address insufficient densification in PM, spark plasma sintering (SPS) readily enables a density greater than 98%. However, it is still unlikely to offer a homogeneous microstructure [67]. As such, homogenization through long post-sintering heat treatment is essential. For example, Bahador et al. [68] held the SPSed Ti-67Ta at 1500 °C for up to 12 h, which produced a homogeneous but much coarser microstructure. In addition, hot working can eliminate the residual pores in the as-sintered material, but achieving composition homogenization simultaneously is practically inviable. Interestingly, it can lead to some unique microstructures; for example, the hot working of inhomogeneous PM Ti-Ta alloys has led to a lamellar structure with alternating Ti-rich and Ta-rich lamellae [[69], [70], [71]].

Opposite to pursuing full densification, PM is a practically effective approach to fabricating porous Ti-Ta alloys [56]. Powder compaction at low pressures combined with pressureless sintering at relatively low temperatures can well retain the interparticle voids, offering high porosities, e.g., a porosity of 28-35% and an average pore size of 6-17 μm obtained by Ibrahim et al. [72] in Ti-61.8Ta. With the use of space holders such as magnesium [73], ammonium bicarbonate [74], sodium chloride [75], or polyvinyl alcohol [76], PM enables a delicate manipulation of the porosity and pore size in Ti-Ta alloys by adjusting the volume fraction of space holders and their particle size.

### 2.3 Other conventional approaches

Apart from melting and PM processes, other high-temperature processes can also synthesize Ti-Ta alloy. For example, Wang et al. [77] prepared Ti/Ta multilayer composites by diffusion bonding alternatively stacked Ti/Ta foils (Ti: 100 μm thick; Ta: 30/50/100 μm thick) with the use of SPS, generating thin Ti-Ta alloy layer of 32-72.6 μm thick at the Ti/Ta interface when sintered at 1000-1400 °C for 10 min at a pressure of 21 MPa. The underlying mechanism is solid-state diffusion, the same as that of PM processing of Ti-Ta alloys. As such, the alloying extent or the microstructure homogeneity is determined by the processing temperature and/or duration time at the temperature. Arc welding packed plates of pure Ti (1.6 mm thick) and Ta (0.1 mm thick) at a voltage of 24 V, current of 60 A, and speed of 2 mm/s induced the diffusion of Ta into Ti, forming a thin Ti-Ta alloy layer at the interface [78].

On the other hand, the synthesis of Ti-Ta alloy via mechanical alloying seems different from all the approaches above, as the alloying is realized at a relatively low temperature. X-ray diffraction (XRD) and microscopy analyses show that Ti and Ta elements evolve over milling time from powder particle mixing to micro-scale welding among severely deformed particles to atomic-scale bonding [79].

The Metalysis-Fray Farthing Chen (FFC) process offers another different approach to Ti-Ta alloy synthesis, starting with Ti and Ta oxides [80]. In the process, a mixture of TiO_2_ and Ta_2_O_5_ powder in a specified weight ratio acts as the cathode, while graphite acts as the anode. The cathode and anode are sunk in molten salt electrolyte CaCl_2_ at 950 °C, and then a constant current (5 A) and voltage (∼3.5 V) are applied between the electrodes; TiO_2_ and Ta_2_O_5_ in the cathode are reduced simultaneously by calcium ions, and the released metals bond to form Ti-Ta alloy; after thorough washing in hot water, the product is ground into irregular powder with a pestle and mortar. This process is incredibly flexible in terms of alloy composition design, simply by adjusting the TiO_2_ and Ta_2_O_5_ powder ratio. The Ti-Ta powders have a uniform microstructure, and Ta concentration fluctuates between the hexagonal α and cubic β phases.

### 2.4 Additive manufacturing – powder bed fusion

#### 2.4.1 Fabrication of dense parts

##### 2.4.1.1 Build density

**Fig. 2:**
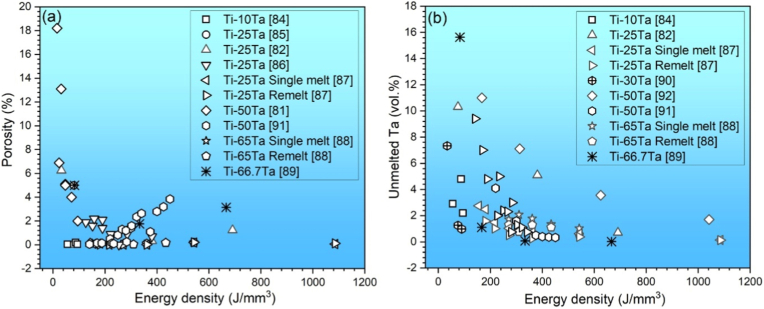
(a) Porosity and (b) unmelted Ta percentage of Ti-Ta alloys as a function of laser energy density of PBF-LB/M, using blended elemental Ti and Ta powders.

Additive manufacturing of Ti-Ta alloys has been attempted intensively via powder bed fusion (PBF) technology, using either a laser (PBF-LB/M) or an electron beam (PBF-EB/M) source. The printing is conducted in high-purity argon (PBF-LB/M) or high vacuum (PBF-EB/M) to minimize oxygen pick-up. Printing parameters, such as laser/electron beam power, scanning speed, layer thickness, and hatching distance, impact the quality of as-built alloys [47,[81], [82], [83], [84], [85], [86], [87], [88], [89], [90], [91], [92]]. For instance, surface roughness and porosity of the resulting Ti-25Ta alloy initially decreased and then increased as laser scanning speed increased from 100 mm/s to 2500 mm/s [83]. The effect of these printing parameters can be depicted as volumetric energy density ($E_{d}$) as follows:

(1) $$E_{d}=\frac{P}{vhd}$$

where P, v, h and d, respectively, are laser/electron beam power, scanning speed, hatching distance, and layer thickness. Fig. 2a shows the porosity of Ti-Ta alloys fabricated by PBF-LB/M with different $E_{d}$ [81,82,[84], [85], [86], [87], [88], [89],^91^]. There seems to exist an optimal $E_{d}$ range to produce highly dense Ti-Ta parts: the higher the Ta content, the higher the optimal $E_{d}$ range, i.e., approximately 50-100 J/mm^3^ for Ti-10Ta, 150-300 J/mm^3^ for Ti-25Ta, and 270-360 J/mm^3^ for Ti-65Ta. Besides the energy density, other factors influence the printing outcomes, as indicated by the significant porosity difference (0.02% vs. 5.0%) in Ti-25Ta printed at essentially the same volumetric energy density (∼80 J/mm^3^) by Gao et al. [82] and Zhao et al. [85]. In these two studies, the laser spot size (40 μm vs. 80 μm) and layer thickness (20 μm vs. 50 μm) are supposed to be influential. As the spot size defines the actual energy density of each single-track scanning (replace hatching space with laser spot size in eq. (1)) [93], while the layer thickness determines the packing density of the powder bed [94]. The smaller laser spot size and thinner layers employed in Gao et al.’s work resulted in a higher energy density (113.6 J/mm^3^ vs. 70.8 J/mm^3^) and packing density, contributing to its lower porosity (0.02%). This is supported by Zhao et al.’s report [86]. They reduced the layer thickness from 50 μm in Ref. [85] to 30 μm with other parameters and powder feedstock unchanged, and achieved a relatively smaller build porosity of 1.9% (vs. 5.0% in Ref. [85]).

##### 2.4.1.2 Powder feedstock

Feedstock materials used in most studies of AM Ti-Ta alloys are elemental Ti and Ta powders. This *in situ* alloying strategy is not only cost-effective but also versatile for composition design. Considering the rapid melting/solidification nature of the AM process, however, such a blended elemental (BE) powder approach makes it difficult to entirely dissolve Ta particles, particularly in high Ta-content alloys [95,96], even using a remelting strategy [87,88]. Replacing Ta with TaH_2_ powder was found to benefit Ta dissolution in Ti-10Ta build but unmelted Ta was still detectable [97]. A higher energy input enhances its dissolution, as shown in Fig. 2b [82,84,[87], [88], [89],^91^,^92^]. However, this may induce the evaporation of Ti powder since the boiling point of Ti (3287 °C) is rather close to the melting point of Ta (2996 °C), which in turn increases the porosity [89]. As a result, achieving pore-free and simultaneously unmelted Ta-free builds has remained challenging for Ti-Ta system to date, as proved by recent comprehensive studies of Ti-(30, 50)Ta alloys [90,98]. This dilemma is more intractable for PBF-EB/M than PBF-LB/M since the former runs under a high vacuum, favoring metal evaporation and powder spatter [99].

Pre-alloyed (PA) Ti-Ta powder can circumvent the aforementioned porosity-alloying challenge with the BE approach [[100], [101], [102], [103], [104], [105]]. Depending on the composition, such powder has a narrow liquidus temperature range (approximately 1700 °C-1900 °C), much lower than Ta's melting temperature. Therefore, the optimal energy density can be lowered to mitigate Ti evaporation. The advantages of using PA powder have been justified experimentally in a few studies. Recently, Polozov et al. [103] prepared nearly fully dense Ti-15Ta (>99.5% dense) from PA powder by PBF-LB/M at $E_{d}$ = 60-112 J/mm^3^; likewise, Ying et al. [102] achieved pore-free Ti-30Ta (99.97% dense) from PA powder at $E_{d}$ = 177 J/mm^3^; both alloys exhibited a homogeneous microstructure. Lauhoff et al. [101,104] attempted both PBF-LB/M and PBF-EB/M approaches to assess PA Ti-61.8Ta powder feedstock, respectively achieving a relative density of 99.99% at $E_{d}$ = 156.8 J/mm^3^ and 99.92% at $E_{d}$ = 66.67 J/mm^3^. Nevertheless, regions with incompletely dissolved Ta were still observed particularly in the as-built microstructures (see Section 3.4.2), which was ascribed to unmelted Ta in the powder feedstock.

##### 2.4.1.3 Process window

Under the criterion of build density, a process window can be defined for PBF-LB/M processing of a given Ti-Ta alloy. Gao et al. [82] presented such an attempt, identifying the parameters – around p = 100 W and v = 1100 mm/s, optimal for Ti-25Ta alloy with a resulting porosity of <1% if h = 40 or 60 μm. When printing at h = 80 μm, however, these optimal parameters yielded a porosity of ∼4%. The authors drew a second-order function from fitting the porosity-parameter variations:

(2) $$Porosity=0.84-0.775p+2.77v+1.21h-0.725ph-0.3vh+1.35p^{2}+3.5v^{2}+1.98h^{2}$$

**Fig. 3:**
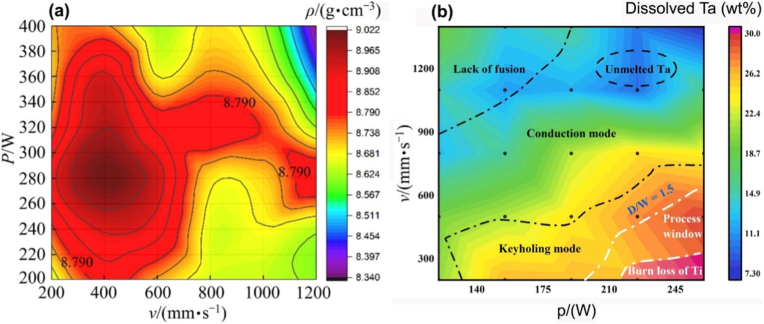
Process window for PBF-LB/M processing of Ti-Ta alloys: (a) a density map of Ti-66.7Ta with respect to the laser power and scanning speed; the alloy has a theoretical density of 8.79 g/cm^3^ [89], (b) a process map of Ti-30Ta defined against the criterion of Ta concentration in the matrix – an indicator of *in situ* alloying degree [90].

Likewise, Zhang et al. [89] produced a density map of Ti-66.7Ta alloy across p = 200-400 W and v = 200-1200 mm/s (Fig. 3a), out of which the best operating window was defined against a build density criterion of 8.79 g/cm^3^. The resulting process window covers a broad range of laser power and scanning speed, unlike that of the Ti-25Ta alloy above. Interestingly, Ti evaporation and lack-of-fusion did not occur at the top/bottom energy densities. Besides building density, including unmelted Ta in the criterion for process window definition will lead to a more complex process map. Fig. 3b is such an example derived from Ti-30Ta, which correlated single-track melt pool modes with dissolved Ta content across varying printing parameters [90]. With increasing energy density, the melt pool sequentially underwent lack-of-fusion, conduction, keyholing, process window, and Ti evaporation zones, as indicated by the dashed lines in the map (Fig. 3b). The process window proposed is situated between the keyholing zone and the Ti evaporation zone, justifying its extreme sensitivity to energy input. It should be noted that the process window discussed above appears to be only applicable to the alloy composition explored in the work, and any changes in alloy composition, laser sources, or unexamined printing parameters can yield a different outcome. Furthermore, there has been no successful case of AM Ti-Ta alloys simultaneously free of internal pores and unmelted Ta particles. As can be inferred from Fig. 2, producing a pore-free build is readily achievable, but it seems unlikely to completely remove unmelted Ta without incurring Ti evaporation. A small amount of unmelted Ta reportedly has little effect on the mechanical properties of Ti-Ta alloys [82,106], and its effect on the biological response is insignificant as well [107]. In this regard, we propose that the priority for AM processing of Ti-Ta alloys is to pursue a high density and then minimize the unmelted Ta.

#### 2.4.2 Fabrication of lattice structures

Lattice structure – a sub-category of porous or cellular structures, is a periodic arrangement of porous unit cells that comprise interconnected struts, plates, or skeletons at a unit length scale mostly less than 10 mm [108]. Metal AM, especially PBF, enables the design and manufacture of intricate lattice materials in various topologies at a unit length scale of down to sub-millimetres. Ti-Ta alloy in a lattice structure provides multiple benefits to meet biomedical implants' mechanical and biological demands: i) lattices can reduce elastic constant by a maximum of two orders of magnitude, allowing Ti-Ta orthopedic implants to match human bones' elasticity; ii) as compared to a dense material, a lattice implant provides enormous surfaces for bone cell adhesion, proliferation, and differentiation; it also provides open and interconnected pores to improve bone cell ingrowth, implant fixation, and flow transport of nutrients and metabolic waste [9,109,110]; and iii) Ti-Ta lattices significantly reduce the density and weight of implants, potentially approaching that of human bones (cortical bone density: 1.72-2.1 g/cm^3^; cancellous bone density: 0.19-0.5 g/cm^3^ [7,14]) at a lowered material cost.

Fabricating Ti-Ta lattices by AM process has been attempted hitherto using PBF-LB/M from BE powders (Table 2). Besides the presence of unmelted Ta particles and pores, as it occurs in dense builds discussed above in Section 2.4.1, the printing of Ti-Ta lattices encounters other manufacturability issues which are associated with factors below:

• **Lattice features.** Lattice features include the lattice topology such as strut-based, skeletal-triply periodic minimal surface (TPMS) based, and sheet-TPMS based structures [108], and strut thickness and inclination in strut-based lattices [111], etc. Sing et al. [47,112] observed significant differences in strut integrity and strut dimensional accuracy between horizontal, vertical, and diagonal struts in Ti-50Ta lattice; Soro et al. [106,113] revealed notable deviations of actual strut thickness, porosity level and pore size from their designed values and their dependence on the skeletal-TPMS based diamond, gyroid and Schwarz lattices in Ti-25Ta (see Table 2).
• **Scanning strategy and parameters.** Ghouse et al. [114] employed a contour scan to print stochastic lattices of Ti-30Ta and identified the dependence of porosity and strut thickness on the exposure time. Strut thickness is primarily associated with the size of the melt pool. It is thus subjected to energy density [115,116], as formulated in Eq. (1). Among the printing parameters, Sing et al. [47] proposed that laser power has a more significant influence on the strut dimension and accuracy than scanning speed and layer thickness.
• **Powder type and alloy composition.** Their effect is predictable, considering the differences between BE and PA powder feedstocks and Ti and Ta metals, as described before.

**Table 2:** Ti-Ta lattice structures printed by PBF-LB/M to date, together with powder feedstock details and major processing parameters.

Lattice topology	Ti-xTa (wt.%)	Powder feedstock	Printing parameters	Design	Outcomes	Ref.
Cubic	50	Ti: D_50_ = 43.5 μm (s^a^) Ta: D_50_ = 44 μm (i^b^)	NA^c^	Cell size: 1 × 1 × 1 mm Porosity: 63% Strut: 285 μm (square)	Porosity = 59.79% Strut = 400-450 μm	[117]
Cubic with diagonal struts	50	Ti: D_50_ = 43.5 μm (s) Ta: D_50_ = 44 μm (i)	P = 120-360 W v = 400-1200 mm/s d = 30-100 μm No hatching	Cell size: 1 × 1 × 1 mm Porosity: NA Strut: 80 μm (square)	Lattice porosity/strut dimensional accuracy were sensitive to laser power/scanning speed/layer thickness and strut inclination, and good-quality lattices were achievable with appropriate printing parameters.	[47]
Auxetic (A3P)	25	Ti: 15-53 μm (s) Ta: 15-45 μm (s)	P = 100 W v = 1000 mm/s h = 60 μm d = 20 μm	Cell size: 3.5 × 3.5 × 3.5 mm Porosity: 85% Strut: 400 μm (cylindrical)	Achieved lattices as designed.	[83]
Diamond	25	Ti: 15-53 μm (s) Ta: 15-45 μm (s)	P = 100 W v = 1000 mm/s h = 60 μm d = 20 μm	Cell size: 3.5 × 3.5 × 3.5 mm Porosity: 85% Strut: 600 μm (cylindrical)	Achieved lattices as designed.	[83]
F_2_CC_z_	25	Ti: 20-45 μm (s) Ta: 15-45 μm (s)	P = 95 W v = 600 mm/s h = 35 μm d = 25 μm	Cell size: 2 × 2 × 2 mm Porosity: 87% Strut: 350 μm (cylindrical)	Porosity = 55% Vertical strut = 463 μm Diagonal strut = 546 μm	[118]
	65				Porosity = 58% Vertical strut = 478 μm Diagonal strut = 524 μm	
Stochastic	30	Ti: 10-45 μm (s) Ta: 5-25 μm (i)	P = 50 W t = 100-950 μs d = 50 μm No hatching	No unit cell Porosity: NA Strut: 70 μm Strut angle: >30°	Laser of 70 μm in spot size was used. At various exposure times (t), porosity levels from 79% to 92% and strut length to thickness ratios ≫5 were obtained.	[114]
Voronoi tessellation	50	Ti: ≤60 μm (s) Ta: ≤40 μm (s)	P = 100-340 W v = 300-600 mm/s h = 100 μm d = 30 μm	No unit cell Porosity: NA	Voronoi-based lattices were printed at three laser energy densities, leading to porosity levels of about 58%, 39% and 30%.	[119]
Schwartz (TPMS)	25	Ti: 20-45 μm (s) Ta: 15-45 μm (i)	P = 95 W v = 300 mm/s h = 35 μm d = 25 μm	Cell size: NA Porosity: 25%, 42%, 64% Strut: 750, 600, 500 μm Pore: 250, 400, 700 μm	Porosity = 14.3%, 26%, 38.1% Strut = 606 μm (64% porosity)	[106,113]
	25	Ti: 20-45 μm (s) Ta: 15-45 μm (i)	P = 95 W v = 600 mm/s h = 35 μm d = 25 μm	Cell size: 1 × 1 × 1 mm Porosity: 60% Min strut: 330 μm Max pore: 950 μm	Porosity = 49.9% Min strut = 446 μm Max pore = 700 μm	[113]
	10 15 20	Ti: D_50_ = 36.0 μm (s) Ta: D_50_ = 24.6 μm (i)	P = 220 W v = 550 mm/s h = 140 μm d = 30 μm	Cell size: 2.5 × 2.5 × 2.5 mm Porosity: 50% Strut: NA	Achieved lattices with an ordered arrangement of circular openings.	[84]
Diamond (TPMS)	25	Ti: 20-45 μm (s) Ta: 15-45 μm (i)	P = 95 W v = 600 mm/s h = 35 μm d = 25 μm	Cell size: 1 × 1 × 1 mm Porosity: 60% Min strut: 300 μm Max pore: 400 μm	Porosity = 30.7% Min strut = 403 μm Max pore = 276 μm	[113]
Gyroid (TPMS)	25	Ti: 20-45 μm (s) Ta: 15-45 μm (i)	P = 95 W v = 600 mm/s h = 35 μm d = 25 μm	Cell size: 1 × 1 × 1 mm Porosity: 60% Min strut: 400 μm Max pore: 520 μm	Porosity = 48.5% Min strut = 505 μm Max pore = 429 μm	[113]
	25	Ti: D_50_ = 30.3 μm (s) Ta: D_50_ = 3.66 μm (i)	P = 320 W v = 600 mm/s h = 70 μm d = 30 μm	Cell size: 1.5 × 1.5 × 1.5 mm Porosity: 90% Min strut: ∼80 μm (est.) Min pore: ∼420 μm (est.)	Porosity = 87.98% Min strut = ∼350 μm (est.)	[85,86]
	10 15 20	Ti: D_50_ = 36.0 μm (s) Ta: D_50_ = 24.6 μm (i)	P = 220 W v = 550 mm/s h = 140 μm d = 30 μm	Cell size: 2.5 × 2.5 × 2.5 mm Porosity: 50% Strut: NA	Achieved lattices with well-defined struts and uniform cell distribution.	[84]
Split (TPMS)	10 15 20	Ti: D_50_ = 36.0 μm (s) Ta: D_50_ = 24.6 μm (i)	P = 220 W v = 550 mm/s h = 140 μm d = 30 μm	Cell size: 2.5 × 2.5 × 2.5 mm Porosity: 50% Strut: NA	Achieved lattices with rough surfaces and irregularities at strut intersections.	[84]

a s: spherical.

b i: irregular.

c NA: not presented.

Various Ti-Ta lattices have been built via AM, as detailed in Table 2. Two major classes have been attempted, i.e., strut-based and skeletal-TPMS-based lattices. The latter has a smooth transition at the connection points, opposite to sharp corners with strut-based lattices. Its intrinsic minimal surface area can reduce the attachment of partially melted powder particles, delivering better controllability in porosity and strut thickness [108]. Nevertheless, the extent of porosity and strut dimension deviation between the actual build and design still noticeably varies with the topology of skeletal-TPMS lattices. For example, the study by Soro et al. [113] indicates that Schwarz exhibits the least porosity deviation (17%) while gyroid exhibits the least strut deviation (26%) among the three skeletal-TPMS lattices (the third one is diamond) printed under identical conditions. Unlike strut-based and TPMS-based lattices, which consist of periodically arranged unit cells, a stochastic lattice is non-periodic and constructed of randomly connected struts inclined at various angles. This structure offers high porosity levels, for example, reaching 92% after contour scanning with a laser of 50 W in power and 70 μm in spot size for an exposure time of 100 μs with no hatching [114].

### 2.5 Additive manufacturing – directed energy deposition

Direct laser energy deposition (DED-LB/M) is another effective AM process for fabricating Ti-Ta alloys. The manufacturing is operated in a chamber with a controlled oxygen content or using flowing argon as a carrier gas to mitigate oxygen adsorption during printing. Powder feedstock is primarily composed of elemental Ti and Ta, offering excellent flexibility in alloy composition. Like Ti-Ta alloys prepared by PBF-LB/M, unmelted Ta remains present in the DED-LB/M build, and its fraction increases with Ta content [46,120]. Due to its technical limitations, there is no report on the fabrication of Ti-Ta lattices through DED-LB/M. An analogous attempt is laser powder depositing Ti-Ta grids on a Ti-6Al-4V substrate [121]. Nevertheless, porous Ti-Ta alloys can be built via DED-LB/M under appropriate printing conditions [46,120]. Unlike the preset cavities in powder bed fused lattices, the internal pores in DED-LB/M materials originate from the lack of fusion, the gas entrapped in hollow powder particles or shielding gas entrapped in melting pools. These pores migrate and coalesce; small pores escape the melting pools, while some large pores remain as their buoyancy is suppressed by the Marangoni force [122]. As a result, the pore features of DED-LB/M parts, such as porosity, pore distribution, and pore size, are poorly controllable [123], making the design and fabrication of delicate pore structures nearly impracticable.

In addition, the DED-LB/M process can offer an interesting capability to produce Ti-Ta alloy when equipped with a two-hopper powder feeder. As the hopper separately delivers Ti and Ta powders at a regulated flow rate, the alloy composition can be easily adjusted, and even compositionally graded Ti-Ta alloys can be readily prepared [[124], [125], [126], [127]]. For instance, Teixeira et al. [125] deposited 80 mm-long Ti-Ta tracks in which Ta content continuously increased from 3% to 96% within the sample length. This is hardly attainable for the PBF approach without modifying the powder feeding system [128].

## 3 Microstructure of Ti-Ta alloys

### 3.1 Constituent phases in Ti-Ta system

**Fig. 4:**
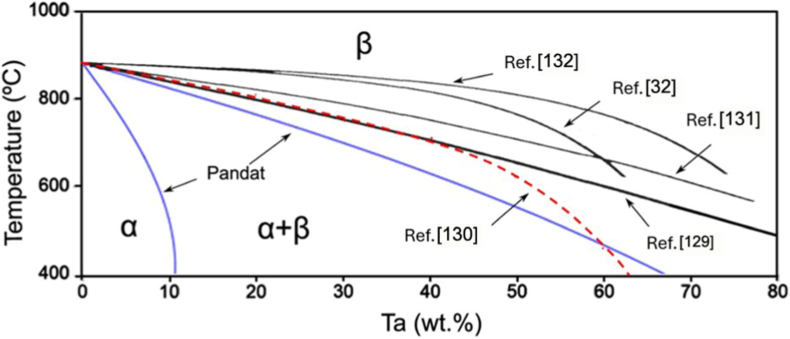
Portion of Ti-Ta phase diagram simulated on Pandat 2022, addressing β to α+β transformation. The location of β/(α+β) phase boundary (solvus) reported in the literature is superimposed [129].

Fig. 4 shows a portion of Ti-Ta phase diagram focusing on the location of β to α+β transformation (β transus), plotted from thermodynamic modeling (Pandat and Ref. [129]) and experimental results [32,[130], [131], [132]]. Due to impurities and/or non-equilibrium cooling conditions, the β transus temperature (T_β-tran_) obtained from experiments is higher than that from simulations, except in the study by Gordin et al. [130], which is in good agreement with the simulation at ≤40%Ta by Murray [129]. Gordin et al. [130] extracted T_β-tran_ of Ti-Ta samples from their electrical resistivity-temperature curves as they were isochronally heated (to 950 °C) and cooled at 2 °C/min in a high vacuum (10^−4^-10^−5^ Pa).

Stable or metastable phases can form in Ti-Ta alloys, mainly dictated by the cooling rate from T_β-tran_ and the Ta content. Stable phases include hexagonal close-packed (HCP) α and body-centered cubic (BCC) β, and their formation requires relatively slow cooling, e.g., cooling rates <10^−1^ °C/s, typically furnace cooling; whereas metastable phases, which include HCP α′ martensite, orthorhombic α'' martensite, athermal HCP or trigonal ω, and BCC β phase, generally prefers moderate cooling like air cooling or rapid cooling like iced water quenching. Sections 2, 3 discuss the constituent phases and their morphology in Ti-Ta alloys fabricated by conventional approaches and AM processes with various cooling rates.

### 3.2 Stable microstructure in conventionally prepared alloys after slow cooling

Table 3 summarizes the stable phases and their morphology in Ti-Ta alloys from slow cooling (e.g., cooling rates <10^−1^ °C/s, typically furnace cooling) with alloy composition and key fabrication procedures. It can be inferred from Table 3 that, after slow cooling, single α phase forms approximately at <10%Ta, single β forms at ≥60%Ta, and two-phase α+β structure forms in compositions between; specifically, α dominates the two-phase α+β structure at 10-40%Ta, while β dominates the structure at 40-60%Ta. Ta is a β-stabilizer in Ti alloys, so increasing its content can induce β phase or increase its fraction. Wang et al. [61] found that as Ta content increased from 15% to 25%, the volume fraction of β phase in as-sintered Ti-Ta alloys increased from 7.1% to 16.2% accordingly. The above information is close to the information we can obtain from the equilibrium phase diagram in Fig. 4: at ∼400 °C, Ti-Ta alloys with <10%Ta consist of single α, and those with ≥60%Ta consist of single β. At ∼37%Ta (Lever Rule), the two-phase α+β structure transits from α-dominant to β-dominant.

**Table 3:** Phase constitution and morphology in Ti-Ta alloys with respect to alloy composition and fabrication approach. Data corresponding to cooling from β-transus temperature are included only.

Ti-xTa (x: wt.%)	Approach and major processing parameters	Phase analysis method	Constituent phases	Phase morphology	Ref.
Slow cooling (cooling rates <10^−1^ °C/s, typically FC)					
5	Melting, homogenization (1000 °C × 3 h in Ar), slow FC	XRD	α+β	Needle-like α + lath-like β	[142]
10	Melting, homogenization (1000 °C × 3 h in Ar), slow FC	XRD	α+β	Needle-like α + lath-like β	[142]
10	Melting, homogenization (T_β-tran_±100 °C), cooling at 5 °C/min	XRD	α	Lamellar	[130]
15	Melting, homogenization (1000 °C × 3 h in Ar), slow FC	XRD	α+β	Needle-like α + lath-like β	[142]
15	Sintering (1600 °C × 3 h in Ar), cooling at 3.5 °C/min	XRD + TEM	α+β	GB/lamellar/acicular α + lath-like β	[61]
15	Sintering (1400 °C × 2 h in Ar), FC	XRD	α+β		[76]
20	Melting, homogenization (1000 °C × 3 h in Ar), slow FC	XRD	α+β	Needle-like α + lath-like β	[142]
20	Melting, homogenization (T_β-tran_±100 °C), cooling at 5 °C/min	XRD	α+β	Widmanstätten α+equiaxed β	[130]
20	Sintering (1600 °C × 3 h in Ar), cooling at 3.5 °C/min	XRD + TEM	α+β	GB/lamellar/acicular α + lath-like β	[61]
20	Melting, homogenization (1200 °C × 48 h in vacuum), FC	XRD + SEM	α+β	Widmanstätten	[54,143]
25	Sintering (1600 °C × 3 h in Ar), cooling at 3.5 °C/min	XRD + TEM	α+β	GB/lamellar/acicular α + lath-like β	[61]
30	Melting, homogenization (T_β-tran_±100 °C), cooling at 5 °C/min	XRD	α+β	Widmanstätten α+equiaxed β	[130]
40	Melting, homogenization (T_β-tran_±100 °C), cooling at 5 °C/min	XRD	α+β	Widmanstätten α+equiaxed β	[130]
40	Melting, HR (900 °C), WR (550-700 °C), AN (810 °C × 0.5 h or 1000 °C × 2 h), FC	XRD	α+β	Not presented	[35]
40	Melting, homogenization (1200 °C × 48 h in vacuum), FC	XRD + SEM	β+α	Needle-like α	[54,143]
48.5	Cold spray, HT ((1000-1250) °C × (2-72) h), cooling at 5 °C/min	XRD	β+α	Equiaxed β + acicular α	[62]
50	Melting, homogenization (T_β-tran_±100 °C), cooling at 5 °C/min	Resistivity	β+α	Equiaxed β + GB α precipitate	[130]
50	Sintering (1000 °C for up to 72 h)	XRD + TEM	β+α	Acicular α + equiaxed β	[56]
50	SPS (850 °C × 0.5 h at 30 MPa), homogenization (1600 °C × 1 h), FC	XRD + TEM	β+α''+α	Equiaxed β + needle-like α''+GB α	[144]
60	Melting, homogenization (T_β-tran_±100 °C), cooling at 5 °C/min	XRD	α+β	Widmanstätten α+equiaxed β	[130]
60	Melting, homogenization (1200 °C × 48 h in vacuum), FC	XRD + SEM	β	Equiaxed	[54,143]
70	Melting, homogenization (T_β-tran_±100 °C), cooling at 5 °C/min	Resistivity	β	Equiaxed	[130]
79	Cold spray, HT ((1000-1250) °C × (2-72) h), cooling at 5 °C/min	XRD	β	Equiaxed	[62]
80	Melting, homogenization (T_β-tran_±100 °C), cooling at 5 °C/min	XRD	β	Equiaxed	[130]
80	Melting, homogenization (1200 °C × 48 h in vacuum), FC	XRD + SEM	β	Equiaxed	[54,143]
Moderate cooling (cooling rate 10° -10^1^ °C/s, typically AC)					
5	Melting, homogenization (1000 °C), AC	XRD	α′	Lamellar	[145]
15	Melting, homogenization (1000 °C), AC	XRD	α′	Lamellar	[145]
15	Melting, AC	XRD	α′	Not presented	[120]
20	Melting, AC	XRD	α′	Not presented	[120]
20	Melting, AC	OM	α′	Acicular	[146]
22	Melting, AC	OM	α'+α''	Needle-like α''+acicular α′	[146]
24	Melting, AC	OM	α'+α''	Needle-like α''+acicular α′	[146]
25	Melting, AC	XRD + TEM	α′	Not presented	[120]
25	Melting, homogenization (1000 °C), AC	XRD	α'+α''	Lamellar α'+acicular α''	[145]
28	Melting, AC	OM	α'+α''	Needle-like α''+acicular α′	[146]
30	Melting, AC	OM	α'+α''	Needle-like α''	[146]
30	Melting, AC	XRD + TEM	α''	Not presented	[120]
35	Melting, AC	XRD	α''	Not presented	[120]
40	Melting, AC	XRD	α''	Not presented	[120]
45	Melting, AC	XRD + TEM	α''	Not presented	[120]
48.6	SPS (1200 °C × 5 min at 40 MPa), HR (700 °C) by 85%, AC	XRD + TEM	α+β	Widmanstätten (α)	[147]
50	Melting, AC	XRD	α''	Not presented	[120]
55	Melting, AC	XRD + TEM	α''	Not presented	[120]
59.5	Melting, HS (950 °C)	XRD + TEM	β+α''+ω	Equiaxed (β)	[137]
60	Melting, AC	XRD	α''	Not presented	[120]
60	Melting, HR (900 °C)	XRD + TEM	β+α''+ω	Plate-like α''+particulate ω	[138]
65	Melting, AC	XRD + TEM	α''	Not presented	[120]
67	SPS (1100 °C × 1 h at 30 MPa), homogenization (1500 °C × 12 h), HF (1100 °C)	XRD + TEM	β+α''	Elongated (β)	[68]
75	Melting, AC	XRD + TEM	β	Not presented	[120]
Rapid cooling (cooling rate >10^2^ °C/s, typically WQ)					
3.7	Melting, rolling, homogenization (1000 °C × 3 h), ice WQ	XRD + TEM	α′	Massive and lath-like	[148]
5.8	Melting, homogenization (1000 °C × 24 h), HR, AN (1000 °C × 3 h), ice WQ	XRD	α′	Feather-/plate-like	[33]
10	Melting, HF (1000 °C), HT (1000 °C × 1 h), WQ	XRD	α′	Not presented	[149]
10	Melting, HT (1100 °C × 24 h), WQ	XRD	α′	Lamellar	[150]
20	Melting, HF (1000 °C), HT (1000 °C × 1 h), WQ	XRD	α′	Not presented	[149]
20	Melting, HT (1100 °C × 24 h), WQ	XRD	α′	Lamellar	[150]
22.1	Melting, homogenization (1000 °C × 24 h), HR, AN (950 °C × 3 h), ice WQ	XRD + TEM	α′	Plate-like	[33]
25	Melting, homogenization (1100 °C × 1 h), WQ, heating (700-1000 °C × 0.5 h), WQ	XRD + TEM	α''+β	Needle-like α'' + β	[151]
25	Melting, HR (1000 °C), ST ((500-1000) °C × 6 h or 1000 °C × (0-6 h)), WQ	XRD	α''	Needle-like	[152]
25	Melting, cooling in water-cooled Cu crucible	XRD	α'+α''	Acicular	[153]
29.6	Melting, rolling, homogenization (1000 °C × 3 h), ice WQ	XRD + TEM	α'+α''	Plate-like	[148]
30	Melting, HF (1000 °C), HT (1000 °C × 1 h), WQ	XRD	α'+α''	Not presented	[149]
30	Melting, HT (1100 °C × 24 h), WQ	XRD	α''+β	Needlie-like	[150]
32.2	Melting, homogenization (1000 °C × 24 h), HR, AN (850 °C × 3 h), ice WQ	XRD + TEM	α''	Needle-like	[33]
36	Melting, homogenization (1000 °C × 24 h), HR, AN (850 °C × 3 h), ice WQ	XRD	α''	Needle-like	[33]
40	Melting, HT (1100 °C × 24 h), WQ	XRD	α''+β	Needlie-like	[150]
40	Melting, HR (900 °C), WR (550-700 °C), AN (1000 °C × 2 h), WQ	XRD + TEM	α''	Not presented	[35]
40	Melting, HR (850 °C), AN (1000 °C × 0.5 h), ice WQ	XRD + OM	α''	Needle-like	[154]
40	Melting in a water-cooled Cu crucible	XRD	α''+β	Equiaxed β+needle-like α''	[155]
40	Melting, HF (1000 °C), HT (1000 °C × 1 h), WQ	XRD	α'+α''	Not presented	[149]
40	Melting, rolling, homogenization (1000 °C × 3 h), ice WQ	XRD + TEM	α''	Plate-like	[148]
48.6	Melting, rolling, homogenization (1000 °C × 3 h), ice WQ	XRD + TEM	α''	Plate-like	[148]
53.2	Melting, homogenization (1000 °C × 24 h), HR, AN (1000 °C × 40 h), ice WQ	XRD + TEM	α''	Needle-like	[33]
55	Melting, HR (850 °C), AN (1000 °C × 0.5 h), ice WQ	XRD + OM	α''+β	Equiaxed β+needle-like α''	[154]
55.8	Melting, rolling, homogenization (1000 °C × 3 h), ice WQ	XRD + TEM	α''+β	Plate-like (α'')	[148]
60	Melting, homogenization (1000 °C × 6 h), ST (950 °C × 1 h), WQ	XRD + TEM	β+α''	Equiaxed β+needle-like α''	[156]
60	Melting, HR (900 °C), HT ((700-900) °C × 1 h), ambient WQ	XRD + TEM	β+α''+ω	Plate-like α''+particulate ω	[138]
61.8	Melting, rolling, homogenization (1000 °C × 3 h), ice WQ	XRD + TEM	β+α''	Equiaxed (β)+α'' plate	[148]
61.8	Melting, AN (1100 °C × 25 h), WQ	OM + TEM	α''	Needle-like	[55]
61.8	Melting, homogenization (1100 °C × 25 h), CR, HT (900 °C × 10 min), WQ	XRD + SEM	α''	Needle-like	[141]
65	Melting, HR (850 °C), AN (1000 °C × 0.5 h), ice WQ	XRD + OM	β+α''	Equiaxed β	[154]
67.1	Melting, rolling, homogenization (1000 °C × 3 h), ice WQ	XRD + TEM	β	Equiaxed	[148]
71.6	Melting, rolling, homogenization (1000 °C × 3 h), ice WQ	XRD + TEM	β	Equiaxed	[148]

Note: Techniques used in the fabrication and characterization of Ti-Ta alloys include spark plasma sintering (SPS), hot forging (HF), hot rolling (HR), warm rolling (WR), hot swaging (HS), cold rolling (CR), solution treatment (ST), heat treatment (HT), annealing (AN), air cooling (AC), furnace cooling (FC), water quench (WQ), optical microscopy (OM), X-ray diffraction (XRD), and transmission electron microscopy (TEM).

**Fig. 5:**
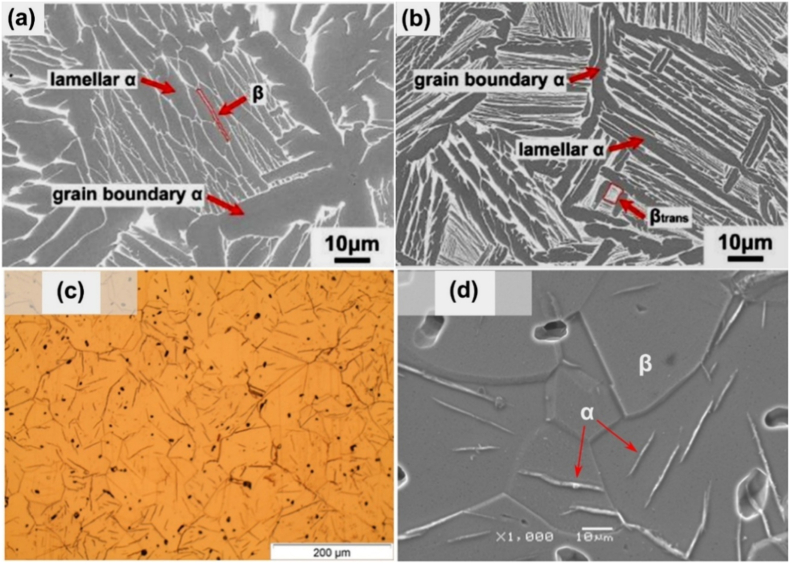
Typical microstructures of Ti-Ta alloys consisting of stable α and β phases: SEM image of an α-dominating α+β structure observed in as-sintered (a) Ti-15Ta, and (b) Ti-25Ta, mainly containing lamellar α and lath-like β, along with secondary grain boundary α [61]. The powder compacts were sintered at 1600 °C for 3 h in Ar flow and cooled at 3.5 °C/min; (c) optical microscope and (d) SEM image of a β-dominating α+β structure in as-sintered Ti-50Ta with equiaxed β grains and needle-like α precipitates in β matrix [56]. The Ti-50Ta was fabricated by isothermal sintering of cold isostatic pressed powder compacts at 1000 °C for 24 h.

Regarding the microstructure of Ti-Ta alloys with stable phases, a single α phase exhibits a lamellar morphology, and a single β phase exists as equiaxed grains. In contrast, the two-phase α+β microstructure varies with the phase fractions [44,130]. Alloys dominated by the α phase consist of lamellar α and lath-like β embedded in the α matrix (Widmanstätten-type structure). Fig. 5a and b shows such a structure observed in as-sintered Ti-(15-25)Ta [61]. In contrast, Ti-Ta alloys dominated by β phase are usually composed of equiaxed β grains embedded with needle-like α phase [56,68,133], as shown in Fig. 5c and d for as-sintered Ti-50Ta [56].

### 3.3 Metastable microstructure in conventionally prepared alloys after moderate and rapid cooling

Cooling Ti-Ta alloy from T_β-tran_ at a moderate rate (cooling rate 10°-10^1^ °C/s, typically air cooling) or rapid (cooling rate >10^2^ °C/s, typically water quenching) produces a metastable microstructure, consisting of HCP α′ martensite, orthorhombic α'' martensite, athermal HCP or trigonal ω, BCC β phase or their combinations. The alloy's phase constitution is primarily determined by its Ta content (Table 3), roughly a single α′ phase at < 26%Ta, single α'' phase at 23-53%Ta, two-phase α''+β at 52-65%Ta, and single β phase at >65%Ta [31,33,34,134]. Nevertheless, slightly different threshold Ta percentages have been reported, attributed mainly to the composition inhomogeneity and/or phase identification methods.

According to studies by Fedotov et al. [37], Ikeda et al. [135], and Zhou et al. [136], Ta content for the athermal ω phase is limited to approximately 50-65%, which virtually overlaps that of the two-phase α''+β structure in Ti-Ta alloy (52-65%Ta). This overlapping is likely associated with the decomposition of α'' martensite to ω phase during cooling. In addition, a moderate (e.g., air cooling after hot working [137]) or quenching into ambient temperature water [138]) from T_β-tran_ is critical for the formation of the ω phase. Yin et al. [139] reported an exception, where ω phase formed in Ti-59.5Ta powder from an ultra-fast cooling process (plasma rotating electrode process, cooling rate: 10^4^-10^6^ °C/s), other than a moderate or slow quench cooling rate discussed above. Because the alloy powder cooled from a droplet state rather than T_β-tran_, their observation may not deviate from the aforementioned cooling principles for forming the athermal ω phase.

Noteworthily, aging of solution-treated and quenched Ti-Ta alloys can produce an isothermal ω phase [140,141]. In the study by Ikeda et al. [135], Ti-50Ta was solution treated at 900 °C for 1 h, quenched into ice water, and then aged for 1 h at 100-900 °C at an interval of 50 °C; ω phase was observed in samples aged at 450 °C or at 500 °C. Zhou et al. [136] presented similar findings in Ti-50Ta. In their work, samples were solution treated at 950 °C for 1 h, quenched into ice water, aged at 450-600 °C for 4-144 h, followed by ice water quench; ω phase was present in samples aged at 450 °C for 4-72 h and aged at 500 °C for 4 h. Additionally, the isothermal ω phase was detected in Ti-Ta alloys with 61.8-64%Ta after aging at 300 °C for 1 h [140]. These observations suggest that the formation of the isothermal ω phase in Ti-Ta alloys prefers an aging treatment at relatively low temperatures for a short period.

### 3.4 Microstructure in additively manufactured dense alloys

**Fig. 6:**
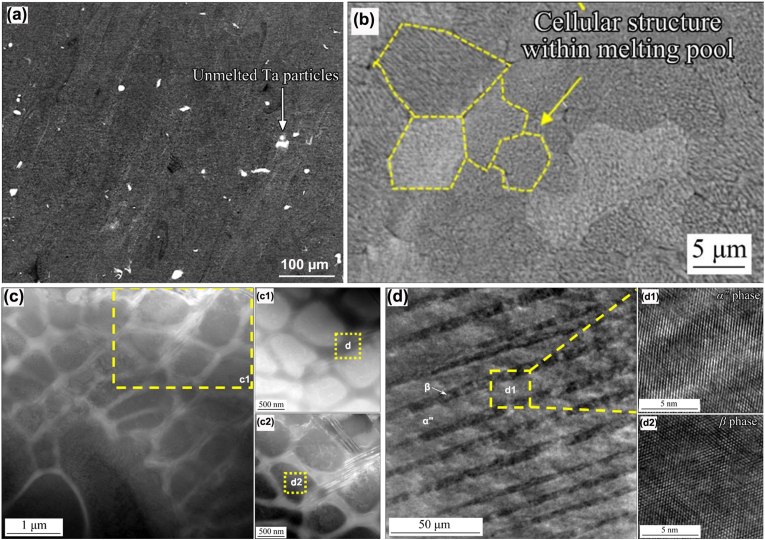
Microstructure characterization of Ti-66.7Ta alloy fabricated by PBF-LB/M [89]: (a) SEM image showing the presence of unmelted Ta particles (1.11 vol%); (b) SEM image of fine cellular structures abundant in AM Ti-Ta alloys, yellow lines depicting prior-β grains; (c) dark-field TEM image of the cellular structures, where dark regions are Ta-rich cells while their bright boundaries are rich in Ti according to EDX analysis; (d) dark-field TEM image of the yellow square c1, showing alternating β (dark) and α'' (bright) lamellae comprising the cell boundary.

Fast cooling inherent with the AM process usually offers as-built Ti-Ta alloys a metastable microstructure. Their constituent phase varies with alloy composition, similar to the variations observed in conventionally fabricated and fast-cooled alloys, as discussed in Section 3.3 (Table 3). Teixeira et al. [125] made an effort to identify the composition-phase relationship with Ta content up to 86%, and detected α′ at <29%Ta, α'' at 29-31%Ta, α''+β at 36-64%Ta, and β at ≥76%Ta in the alloys made by DED-LB/M, based on XRD analysis. This relationship is arguable due to the perturbation from the composition inhomogeneity, which is particularly significant, allowing for the unmelted Ta and composition segregation in local areas. The latter has been confirmed by Zhang et al. through TEM investigation, as shown in Fig. 6 [89]. They detected β phase only in PBF-LB/M Ti-66.7Ta build from XRD but further observed alternating fine lamellae of α'' (∼10 nm thick) and β (∼6 nm thick) at the cell interface from TEM examination.

Besides alloy composition, other factors influence the microstructure of the as-built Ti-Ta materials.

**Fig. 7:**
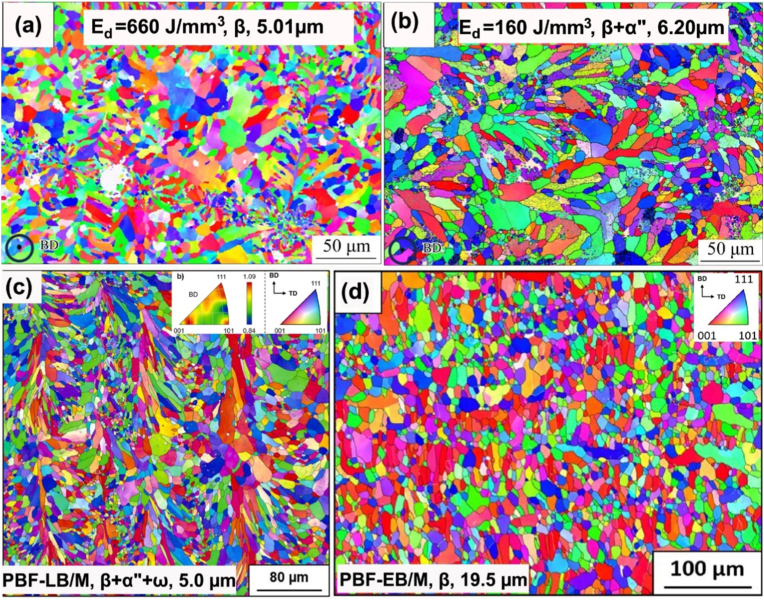
Inverse pole figure maps of AM Ti-Ta alloys showing the effect of printing parameters and approach: Ti-66.7Ta printed at (a) 660 J/mm^3^ or (b) 160 J/mm^3^ by PBF-LB/M [89], (c) Ti-61.8Ta printed by PBF-LB/M [104], (d) Ti-61.8Ta printed by PBF-EB/M [101]. The printing approach or energy density, constituent phase and average prior-β grain size are indicated.
• The printing approach is another factor. Compared to PBF-LB/M, PBF-EB/M has a much higher substrate temperature of up to 750 °C (vs. 200 °C typically adopted in PBF-LB/M) [106], which is close to or even higher than the T_β-Tran_ of Ti-Ta alloys, particularly with high Ta contents (Fig. 4). This can substantially suppress the cooling rate around T_β-Tran_ and shift the phase transformation toward an equilibrium state. As a result, the microstructure of as-built Ti-Ta alloys by PBF-EB/M is close to a stable state. For instance, a single β phase was detected in Ti-61.8Ta printed by PBF-EB/M at a substrate temperature of 540 °C [101]; in contrast, this alloy was comprised of predominant β (66 ± 15 vol%), α'' (21 ± 8 vol%) and ω (13 ± 7 vol%) phases when printed by PBF-LB/M from the same powder feedstock [104]. As indicated in Fig. 7c and d, a slow cooling rate and high substrate temperature offer PBF-EB/M Ti-Ta alloys a coarse microstructure relative to PBF-LB/M counterparts [88,101].

• The first one is the printing parameters, which determine $E_{d}$. The rationale is that increasing energy input promotes the melting and alloying of Ta with Ti, thereby increasing its concentration in Ti-Ta melt; accordingly, the stability of β phase is enhanced, making it less prone to undergo a crystal structure transformation. For example, Ti-25Ta made from BE powders by PBF-LB/M at different energy densities consist of different phases. When printed at $E_{d}$ = 76 J/mm^3^, Ti-25Ta contained 10.3 vol% unmelted Ta and the microstructure was dominated by α′ phase; whereas the same alloy printed at $E_{d}$ = 691 J/mm^3^ had 0.7 vol% unmelted Ta and α'' as the dominant phase in the microstructure [82]. Similar observations were reported in Ti-50Ta [119] and Ti-66.7Ta [89]. Printing parameters can also influence the cooling process, the phase transformation and the final microstructure. Fig. 7a and b compare the microstructure of Ti-66.7Ta, respectively printed at 660 J/mm^3^ and 160 J/mm^3^ by PBF-LB/M [89].

The morphology of constituent α′ and α'' phases in AM Ti-Ta alloys generally resembles that observed in conventionally fabricated and fast-cooled counterparts, whereas β phase exhibits a cellular structure. Besides unmelted Ta particles, the as-built microstructure mainly consists of lath-like α′ at <10%Ta, lath-like α′ or acicular α'' + cellular β at 10-25%Ta, mixed acicular α'' + lath-like α' + cellular β at 25-50%Ta, or cellular β grains at >50%Ta [46,85,86,88,89,91,92,103,104,117]. AM has a higher cooling rate (>10^2^ °C/s for DED-LB/M, and >10^3^ °C/s for PBF-LB/M) than conventional quenching (10^1^-10^2^ °C/s); therefore, the phases in AM Ti-Ta alloys appear finer. At a macro scale, thermal gradient – heat flow caused by successive layers melting, leads to coarse columnar grains orienting along the building direction. In addition to the alloy composition and printing parameters, the feedstock material plays a significant role in the homogeneity of the microstructure, and we discuss this as follows.

#### 3.4.1 Inhomogeneous microstructure from blended elemental powder feedstock

**Fig. 8:**
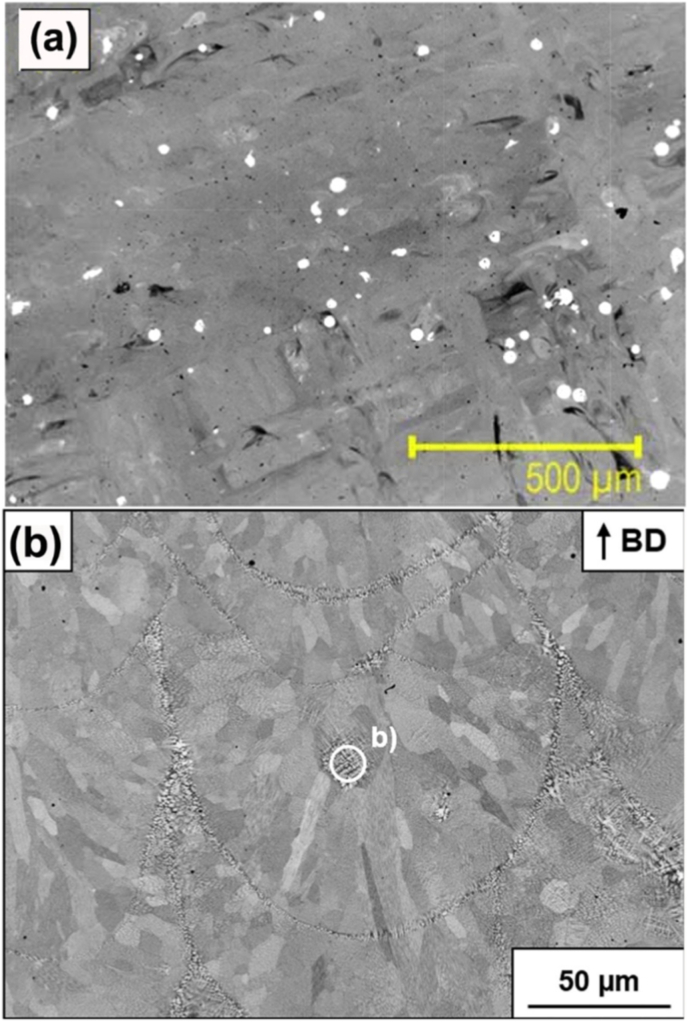
(a) Typical microstructure of Ti-Ta alloy fabricated from blended elemental Ti and Ta powders by PBF-LB/M, where white regions are unmelted Ta particles and dark regions are pores [106]. (b) The microstructure of Ti-61.8Ta alloy prepared from pre-alloyed powder using the PBF-LB/M approach [104], where unmelted Ta is virtually undetectable.

Additively manufactured Ti-Ta alloys from BE powders inevitably contain unmelted Ta (Fig. 2b), causing an inhomogeneous microstructure in the build. Fig. 8a is a typical microstructure from Ti-25Ta fabricated by PBF-LB/M, where plenty of unmelted Ta particles are retained [106]. The percentage of unmelted Ta varies with the energy density as indicated in Fig. 2b and more greatly with the Ta proportion in the BE powder mix. Interestingly, Gao et al. [82] found that Ta concentration around the unmelted Ta particle dropped sharply and no obvious diffusion gradient formed in Ti-25Ta build, according to the energy dispersive X-ray spectroscopy (EDX) result. This implies a weak bond between the Ta particle and the matrix, explaining the insignificant effect of unmelted Ta particles on the mechanical properties (see discussion in Section 4.2). Song et al. [157] detected negligible diffusion between Ti and Ta if printing Ti powder on a Ta substrate at $E_{d}$ = 147-238 J/mm^3^, but printing Ta powder on a Ti substrate induced potent diffusion regardless of $E_{d}$ (220-343 J/mm^3^). More effort is thus warranted to address these controversial phenomena.

#### 3.4.2 Homogeneous microstructure from pre-alloyed powder feedstock

In contrast, PA powder enables a homogeneous microstructure in AM Ti-Ta alloys [[102], [103], [104], [105]]. In Ref. [105], Ti-Ta ingot was initially made by vacuum induction levitation melting, followed by hydrogenation and crushing; the hydride powder was sieved and classified into a desired range of particle size, and then dehydrogenated and spheroidized by radio frequency plasma; last, the resulting Ti-Ta alloyed powder was chemically reduced and further sieved into 15-53 μm for subsequent printing. The thus produced Ti-(25, 55, 75)Ta powder exhibited a spherical shape and uniform element distribution with an oxygen content of 0.063-0.083%. As-built Ti-Ta alloys by PBF-LB/M displayed a homogeneous microstructure with no unmelted Ta observed. In a patent, Sungail et al. [158] disclosed a similar method to prepare spherical Ti-Ta PA powders capable of delivering a homogeneous microstructure to AM Ti-Ta alloy. If the PA Ti-Ta powders originally contain incompletely dissolved Ta, Ta-rich regions are still present in the as-built microstructure [101,104]. However, the overall homogeneity is far better than that fabricated from BE powder. Fig. 8b shows the microstructure of Ti-61.8Ta built from PA powders by PBF-LB/M [104].

### 3.5 Microstructure of additively manufactured lattices

Ti-Ta lattices' phase constitution resembles their dense counterparts if printed similarly. This is because the phase constitution is primarily determined by the alloy composition and cooling rate (Table 3, Table 4, Table 5, Table 6). An example from limited research available is Ti-65Ta made by PBF-LB/M, metastable β phase was identified in both lattices and dense specimens [88,118]. Nevertheless, composition inhomogeneity and/or phase identification methods may bring subtle differences. For instance, the Ti-25Ta lattice mainly consisted of acicular α′ martensite in electron backscattered diffraction (EBSD) results; on the other hand, besides α′, dense Ti-25Ta printed using a similar remelting strategy also has marginal α'' phase in Ta-rich regions detected by TEM [87,118,159].

**Table 4:** Microstructure and tensile properties of Ti-Ta alloys prepared by conventional routes.

Ti-xTa (x: wt.%)	Manufacturing and key parameters		Phases	E (GPa)	σ (MPa)	σ_0.2_ (MPa)	δ (%)	EAS (%)	Ref.
15	Sintering (1600 °C × 3 h in Ar), heating/cooling at 3.5 °C/min		α+β	–a	977	942	2.4	–	[61]
20			α+β	–	1107	1086	1.4	–	
25			α+β	–	1124	1124	1.1	–	
40	Melting, HR^b^ (900 °C), WR (550-700 °C)	As-rolled	α+β	–	1069	786	2	–	[35]
		AN (810 °C × 0.5 h), WQ, ageing (530 °C × 4 h), FC	α+β	–	1200	986	4	–	
		AN (810 °C × 0.5 h), WQ	α''+β+α	–	814	469	20	–	
		AN (1000 °C × 2 h), FC	α+β	–	793	793	1	–	
20	SPS (1200 °C × 5 min at 30 MPa), HR (600 °C) by 80%, AC		α+β	88.036	860.327	-	7.348	–	[161]
30	SPS (1200 °C × 5 min at 30 MPa), HR (600 °C) by 30%, AC		β+α	–	1085	870	5.2	–	[162]
48.6	SPS (1200 °C × 5 min at 40 MPa), HR (700 °C) by 85%, AC	As-rolled	–	–	1108	998	–	–	[147]
		HR + AN (600 °C × 0.5 h), WQ	–	–	1226	950	–	–	
48.6	SPS (1200 °C × 5 min at 40 MPa), HR (800 °C), CR, AN (600 °C), WQ	HR by 20%	β+α	79.8 ± 1.1	910 ± 56	740 ± 27	8.6 ± 0.7	0.93	[69,70]
		HR by 40%	β+α	80.4 ± 0.7	980 ± 47	780 ± 26	8.9 ± 1.4	0.97	
		HR by 60%	β+α	80.6 ± 0.9	1030 ± 61	830 ± 32	10.2 ± 0.6	1.03	
50	SPS (1200 °C × 5 min at 40 MPa), HR (700 °C) by 50%, AC		β+α+α''	82	1060	872	10.9	1.06	[71]
48.6	Sintering (1400 °C × 2 h in vacuum), FC		β+α	74.1	1251	–	–	–	[60]
55.8			β	76.8	1170	–	–	–	
61.8			β	82.4	1056	–	–	–	
67.1			β	76.5	820	–	–	–	
79.1			β	68.4	290	–	–	–	
48.5	Cold spray, HT ((1000-1250) °C × (2-72) h, cooling at 5 °C/min		β+α	83.13	742	–	–	–	[62]
79			β	85.53	604	–	–	–	
61.8	Sintering (1200-1500) °C × 2 h in vacuum), FC		β	89.8	925	–	–	–	[60]
			β	83.8	1000	–	–	–	
			β	82.4	1060	–	–	–	
			β	81.5	755	–	–	–	
67.1	SPS (1100 °C × 1 h at 30 MPa)	As-sintered	β+α	65 ± 3.25	791 ± 9.42	646 ± 7.75	9.5 ± 0.42	0.99	[68]
		Homogenization (1500 °C × 12 h)	β+α	88 ± 4.4	709 ± 8.5	709 ± 8.5	0.8 ± 0.05	0.81	
		Homogenization (1500 °C × 12 h), HF (1100 °C)	β+α''	61 ± 3.05	868 ± 10.41	828 ± 9.93	8.5 ± 0.38	1.36	
20	Melting, AC		α′	88	834	745	12	0.85	[146]
22			α'+α''	72	658	531	14	0.74	
24			α'+α''	80	627	517	20	0.65	
28			α'+α''	80	758	613	14	0.77	
30			α'+α''	87	993	–	0.7	–	
10	Melting (10 times), homogenization (1000 °C × 6 h), CR, ST (950 °C × 1 h), ice WQ		α′	100	510	400	21	0.40	[13,136,163,165,166]
20			α′	82	540	470	19	0.57	
25			α''	64	565	480	20	0.75	
30			α''	69	587	420	21	0.61	
40			α''	81	580	350	23	0.43	
50			α''	88	530	380	25	0.43	
60			β+α''	82	690	500	23	0.61	
70			β	67	600	375	16	0.56	
80			β	101	640	600	11	0.59	[13]
30	Melting, homogenization (1000 °C × 6 h), CR, ST (950 °C × 1 h), ice WQ, ageing for 72 h, ice WQ	ST	α''	69	587	–	21	–	[13,136,163,165,166]
		ST + ageing (500 °C)	α	76	892	–	9.3	–	
		ST + ageing (600 °C)	α+β	93	750	–	12	–	
40		ST	α''	81	580	–	23	–	
		ST + ageing (500 °C)	α+β	97	1025	–	10	–	
		ST + ageing (600 °C)	α+β	87.5	980	–	10	–	
50		ST	α''	88	530	380	25	0.43	
		ST + ageing (500 °C)	α+β	93	1012	877	2.6	0.94	
		ST + ageing (600 °C)	β+α	77	713	612	11.6	0.79	
40	Melting, HR (850 °C), AN (1000 °C × 0.5 h), ice WQ		α''	72	612	–	24.4	–	[154]
55			α''+β	75	578	–	21.3	–	
65			β+α''	66	551	–	17.6	–	
48.6	Sintering (1400 °C × 2 h), HS (1000 °C), AN (1000 °C × 2 h), WQ		β+α+ α'+α''	79	1570	1060	3.5	1.34	[65]
55	Melting, homogenization, HR (900 °C), AN (800 °C × 6 min), ice WQ		α''+β	75.3	578	418	21.3	0.56	[167]
59	Melting, HS (950 °C)		β+α''+ω	89.9 ± 6.1	673.8 ± 78.2	549.4 ± 17.0	28.55 ± 2.19	0.61	[137]
68	SPS (1100 °C × 1 h at 30 MPa), homogenization (1500 °C × 12 h), HF (1100 °C), HT (1500 °C × 1 h), WQ		β	–	800	780	8.4	–	[168]

a –: data not stated or not available.

b see nomenclature in the note of Table 3.

**Table 5:** Tensile properties and microstructure features of as-built Ti-Ta alloys by PBF-LB/M.

Ti-xTa (x: wt.%)	Powder feedstock	Printing parameters	Relative density (%)	Phases	E (GPa)	σ (MPa)	σ_0.2_ (MPa)	δ (%)	EAS (%)	Ref.
0	Ti: D_50_ = 30.3 μm (s) Ta: D_50_ = 3.66 μm (i)	P = 340 W v = 1200 mm/s h = 70 μm d = 50 μm	98.8 ± 0.8	Lath α	115 ± 5	641 ± 10	560 ± 13	∼18.0	0.49	[85]
6			95.6 ± 1.0	Lath α'+Ta	108 ± 4	697 ± 2	595 ± 5	∼17.5	0.51	
12			96.3 ± 1.5	Lath α'+cellular β+Ta	99 ± 6	783 ± 18	650 ± 7	∼14.5	0.66	
18			95.6 ± 1.2	Acicular α'+cellular β+Ta	96 ± 3	808 ± 17	668 ± 20	∼9.7	0.70	
25			95.0 ± 1.1	Acicular α'+cellular β+Ta	89 ± 4	1186 ± 14	1029 ± 8	∼5.5	1.16	
10	Ti: D_50_ = 36.0 μm (s) Ta: D_50_ = 24.6 μm (i)	P = 220 W v = 550 mm/s h = 140 μm d = 30 μm	99.9	Dendritic/cellular α'+β+Ta	118 ± 3	1020 ± 20	885 ± 10	8.7 ± 0.5	0.75	[84]
15			99.4	Dendritic/cellular α'+β+Ta	110 ± 2	1102 ± 17	902 ± 15	5.1 ± 0.3	0.82	
20			99.8	Dendritic/cellular α'+β+Ta	102 ± 3	972 ± 15	828 ± 10	11.1 ± 1.2	0.81	
15	Ti-15Ta: D_50_ = 35.2 μm (s)	P = 250 W v = 800 mm/s h = 100 μm d = 50 μm	99.78	Acicular α'' + cellular β	90 ± 2	545 ± 18	475 ± 14	20.0 ± 1.0	0.53	[103]
		P = 250 W v = 800 mm/s h = 80 μm d = 50 μm	99.62	Acicular α'' + cellular β	88 ± 3	540 ± 7	460 ± 10	22.1 ± 3.0	0.52	
0	Ti: D_50_ = 43.5 μm (s) Ta: D_50_ = 44 μm (i)	NA^a^	99.938	Lath α	127 ± 10	695 ± 16	602 ± 10	10.8 ± 0.2	0.47	[117]
10			99.961	Lath α'+Ta	113 ± 27	780 ± 20	730 ± 12	10.5 ± 0.2	0.65	
30			99.764	Acicular α''+lath α'+Ta	72 ± 13	950 ± 5	920 ± 10	10.0 ± 0.5	1.28	
50			99.534	Equiaxed β+acicular α''+lath α'+Ta	86 ± 7	925 ± 8	900 ± 5	9.4 ± 0.8	1.05	
10	Ti: 15-53 μm (s) Ta: D_50_ = 25 μm (i)	P = 400 W v = 800 mm/s	–b	Lath α'/α+Ta	–	480	∼450	17	–	[97]
	Ti: D_50_ = 43.5 μm (s) TaH_2_: D_50_ = 25 μm (i)	P = 400 W v = 800 mm/s	–	Blocky α'/α+ β+Ta	–	570	∼500	15	–	
25	Ti: 15-53 μm (s) Ta: 15-45 μm (s)	P = 100 W v = 120 mm/s h = 60 μm d = 20 μm	98.76	Lamellar α''+Ta	75 ± 1	856 ± 113	693 ± 52	1.5 ± 0.2	0.92	[82,83]
		P = 100 W v = 220 mm/s h = 60 μm d = 20 μm	99.67	Lamellar α'+α''+β+Ta	80 ± 2	1076 ± 2	795 ± 16	7.5 ± 0.4	0.99	
		P = 100 W v = 1100 mm/s h = 60 μm d = 20 μm	99.98	Lamellar α'+α''+Ta	65 ± 2	771 ± 30	589 ± 17	31.0 ± 1.0	0.91	
25	Ti: D_50_ = 30.3 μm (s) Ta: D_50_ = 3.66 μm (i)	P = 320 W v = 600 mm/s h = 70 μm d = 50 μm	100 (5.66 g/cm^3^)	Acicular α'+ cellular/columnar β+Ta	94.3 ± 0.7	1197 ± 15.9	998 ± 22.1	5.5	1.06	[86]
25	Ti: 20-45 μm (s) Ta: 15-45 μm (s)	P = 95 W v = 100-700 mm/s h = 35 μm d = 25 μm	>99.8 (single melt)	Lath α'+ β+Ta	65 ± 5	509 ± 7	426 ± 14	25 ± 1	0.65	[87,159]
			>99.8 (remelt)	Lath α'+α''+Ta	65 ± 5	566 ± 6	545 ± 9	11 ± 4	0.84	
50	Ti: D_50_ = 43.5 μm (s) Ta: D_50_ = 44 μm (i)	P = 360 W v = 400 mm/s h = 125 μm d = 50 μm	99.9	Equiaxed β+Ta	75.77 ± 4.04	924.64 ± 9.06	882.77 ± 19.60	11.71 ± 1.13	1.16	[81]
50	Ti/Ta mix (s) D_50_ = 32.9 μm	P = 240 W v = 660 mm/s h = 80 μm d = 20 μm	99.86	Equiaxed β+acicular α''+lath α'/α+Ta	–	802.1 ± 9.8	797.4 ± 11.0	19.8 ± 2.3	–	[91]
65	Ti: 15-45 μm (s) Ta: 15-45 μm (s)	P = 95 W v = 350 mm/s	>99.8 (single melt)	Equiaxed β+Ta	81	820	740	10.5	0.91	[88]
			>99.8 (remelt)	Equiaxed β+Ta	96	985	940	6	0.98	
30	Ti: D_50_ = 43.5 μm (s) Ta: D_50_ = 44 μm (i)	P = 190 W v = 1100 mm/s h = 100 μm d = 50 μm	–	α/α'+Ta	–	755	684	8.4	–	[90]
		P = 190 W v = 500 mm/s h = 100 μm d = 50 μm	–	α''+Ta	–	732	639	4.7	–	
		P = 225 W v = 500 mm/s h = 100 μm d = 50 μm	–	α''+Ta	–	745	616	14.7	–	
66.7	Ti: 15-53 μm (s) Ta: <80 μm (i)	P = 280 W v = 200 mm/s h = 70 μm d = 30 μm	96.85	Cellular β+ lamellar α''+Ta	99.31 ± 4.5	1014.71 ± 82.99	–	1.33 ± 0.05	–	[89]
		P = 280 W v = 400 mm/s h = 70 μm d = 30 μm	98.24	Cellular β+ lamellar α''+Ta	42.57 ± 1.32	884.11 ± 34.08	–	10.87 ± 1.56	–	
		P = 280 W v = 800 mm/s h = 70 μm d = 30 μm	99.87	Cellular β+ lamellar α''+Ta	36.70 ± 2.11	806.09 ± 9.38	–	15.66 ± 1.27	–	
0	PA Ti-Ta: 15-53 μm (s)	P = 200 W t^c^ = 50 μs h = 65 μm d = 30 μm	99.9	α′	115	395	–	–	–	[105]
25		P = 200 W t = 50 μs h = 65 μm d = 30 μm	99.3	α′	88	1093	–	–	–	
55		P = 200 W t = 60 μs h = 65 μm d = 30 μm	96.7	β+α′	891 ± 5	74 ± 4	–	–	–	
75		P = 200 W t = 70 μs h = 65 μm d = 30 μm	100	β	875	99	–	–	–	

a NA: not presented.

b –: data not stated.

c t: exposure time.

**Table 6:** Microstructural features and effective compressive properties of Ti-Ta lattices made by PBF-LB/M process. Compressive properties of human bones (femur and tibia only) are incorporated for comparison.

Ti-xTa (x: wt.%)	Topology	Design porosity (%)	Actual porosity (%)	Strut size (μm)	Phases	E (GPa)	σ (MPa)	σ_0.2_ (MPa)	δ (%)	EAS (%)	Deformation/failure mode	Ref.
25	F_2_CC_z_	87	55 ± 0.04	463 ± 33 (V^e^) 546 ± 40 (D^e^)	α'+Ta	10.4 ± 0.1^f^	534±9^f^	∼395^f^	>30	∼3.8	Buckling	[118]
25	Dense	0	0.2 ± 0.07	–	α''+Ta	73.5 ± 1.8	–	620 ± 27	>70	0.84	Barrelling	[106]
	Schwarz	25	14.3 ± 1.7	750 (design)	NA	36.1 ± 2.2	–	319 ± 8		0.88		
		42	26 ± 1.0	600 (design)	NA	23.3 ± 1.5	–	233 ± 2		1.00		
		64	38.1 ± 5.4	500 (design)	NA	14.3 ± 0.7	–	86 ± 7		0.60		
25	Dense	0	–	–	α'+α''+Ta	70.3 ± 5.2	1458 ± 212	682 ± 55	32 ± 10	0.97	45° cleavage	[106,113]
	Gyroid	60	48.5 ± 1.3	505 ± 16.4	NA	10.4 ± 1.5	171 ± 8	74 ± 4	>70	0.72	Barrelling	
	Diamond	60	30.7 ± 2.4	403 ± 18	NA	15.8 ± 0.5	236 ± 6	126 ± 11	>70	0.80	Barrelling	
	Schwarz	60	49.9 ± 0.9	446 ± 33	NA	18.9 ± 2.0	371 ± 56	102 ± 9	26 ± 8	0.54	Buckling	
25	Gyroid	90	87.98	–	α'+β+Ta	1.83 ± 0.1	76.24 ± 2.8	55.52 ± 2.1	45	3.03	∼45° crushing	[86]
25	Auxetic (A3P-S)	85	–	–	α'+Ta (solid)	0.14 ± 2	54.3 ± 2	9.2 ± 0.42	>47	6.57	NA	[83]
	Diamond		–	–		0.34 ± 1	19.9 ± 0.2	15.6 ± 0.38		4.59	∼45° crushing	
25	NA^c^	–d	64.69 ± 1.47	413 ± 23.5	NA	2.26 ± 0.20	109.52 ± 4.13	–	>40	–	Barrelling	[176]
25	NA	80	–	200	α′	2.15	50.83	–	–	–	NA	[105]
30^a^	Stochastic	–	79	–	α''+Ta	3.1 ± 0.1	–	41.3 ± 0.4	>50	1.33	Progressively yielding and angled crushing	[114]
			83	–		2.3 ± 0.0	–	30.1 ± 0.1		1.31		
			86	–		1.6 ± 0.0	–	21.1 ± 0.1		1.32		
			89	–		1.0 ± 0.0	–	12.3 ± 0.1		1.23		
			92	–		0.5 ± 0.0	–	6.3 ± 0.0		1.26		
50	Cubic with diagonal strut	85	63	190 ± 30 (V) 210 ± 40 (D)	–	3.9	–	119	–	3.05	NA	[112]
		87	71.5	250 ± 40 (V) 250 ± 40 (D)	–	3.3	–	80	–	2.42		
		86	73.5	230 ± 20 (V) 260 ± 20 (D)	–	2.5	–	65	–	2.60		
		84	68	190 ± 30 (V) 230 ± 20 (D)	–	3.0	–	81	–	2.70		
		88	74.5	200 ± 20 (V) 230 ± 20 (D)	–	2.4	–	57	–	2.38		
50	Voronoi tessellation	–	58	–	α/α'+Ta	2.3	–	45	>50	1.96	Barrelling	[95,119]
			39	–	α''+Ta	6.2	–	142	>50	2.29	∼45° crushing	
			30	–	β+α''+Ta	7.2	–	200	30-40	2.78	∼45° crushing	
50	NA	–	61.84 ± 6.60	405 ± 7.3	NA	1.07 ± 0.21	89.83 ± 3.26	–	>40	–	Barrelling	[176]
55	NA	80	–	200	β+α′	1.91 ± 0.08	48.86 ± 2.49	–	–	–	NA	[105]
65	F_2_CC_z_	87	58 ± 0.04	478 ± 20 (V) 524 ± 40 (D)	β+Ta	9.7 ± 0.1^f^	490 ± 18^f^	∼377^f^	>30	∼3.9	∼45° crushing	[118]
75	NA	–	65.87 ± 5.54	417 ± 24.5	NA	1.31 ± 0.03	96.63 ± 4.14	–	>40	–	Barrelling	[176]
75	NA	80	–	200	β	2.24	45.72	–	–	–	NA	[105]
Cortical bone^b^	NA	NA	3-12	NA	NA	15.4-35.3	179-213	–	–	0.6-1.2	NA	[14]
Cancellous bone^b^	NA	NA	50-90	NA	NA	0.02-0.83	2.2-10.6	–	2.05-8.30	1.2-13	NA	

a Yield strength is the stress at 1% strain offset.

b EAS of human bones (tibia and femur only) is calculated using compressive strength as yield strength.

c NA: not presented, or not applicable.

d –: data not stated.

e V is for vertical struts while D is for diagonal struts.

f Data recalculated with lattice porosity included.

**Fig. 9:**
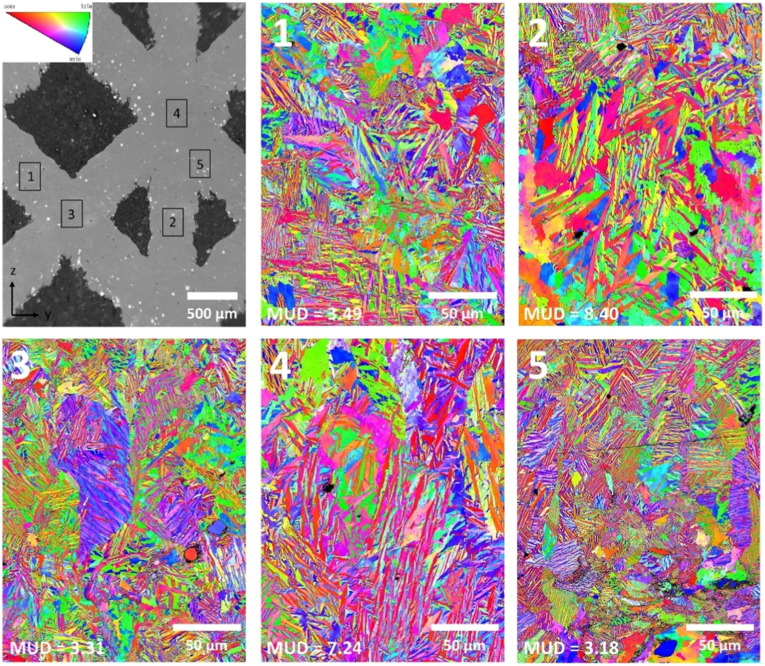
SEM image and EBSD inverse pole figure maps showing the microstructure in different regions of a Ti-25Ta lattice printed by PBF-LB/M in Z direction in a remelt mode [118].

Like their dense counterparts, Ti-Ta lattices also encounter thermal gradients. However, its effect on the microstructure is related to the inclination of the lattice strut/node with respect to the building direction. This is because the thermal gradient of a specific strut or node is directed by not only the heat from their melt pool but also the heat from their nearest struts or nodes. As a result, the microstructure of a strut or node appears to depend on its location or orientation relative to the building direction. Fig. 9 presents an example detected from the Ti-25Ta lattice [118]. A significant fraction of α′ laths formed in the vertical strut (region 2) and node (region 4), visibly orienting along the building direction Z. In contrast, the α′ laths in the diagonal strut (region 1) or node (region 3) distribute randomly. This strut/node inclination-dependent microstructure occurred in Ti-65Ta lattice too, where some elongated/columnar β grains formed in vertical struts and nodes [118]. In contrast, equiaxed β grains formed in diagonal struts or nodes [118].

## 4 Mechanical properties of Ti-Ta alloys

### 4.1 Mechanical properties of conventionally prepared alloys

#### 4.1.1 Alloys with stable phases

**Fig. 10:**
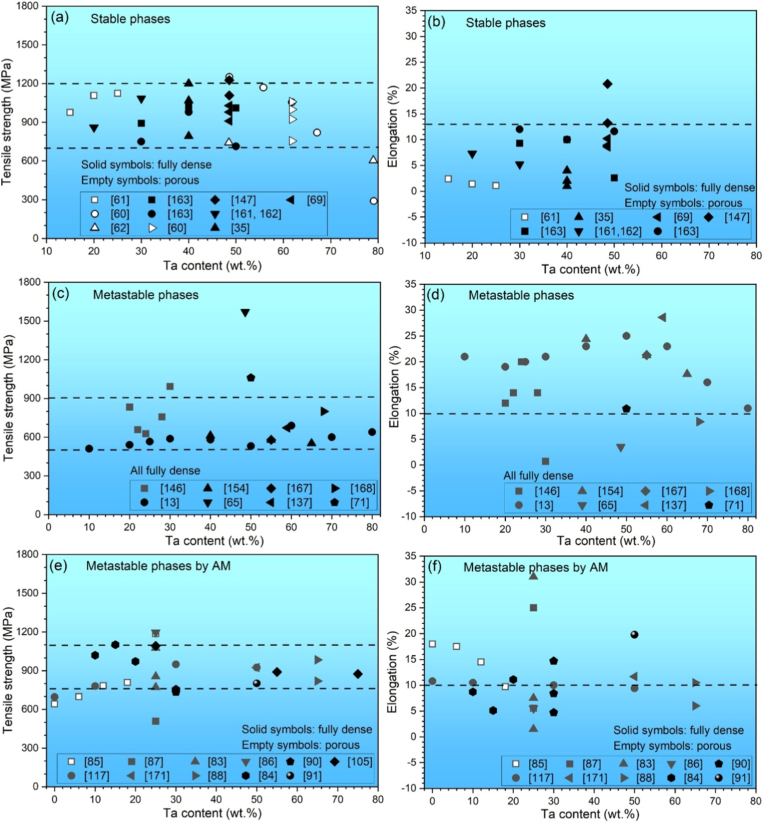
Ultimate tensile strength and elongation of Ti-Ta alloys fabricated by various approaches as a function of Ta content. (a, b) Conventionally fabricated alloys consisting of stable phases, (c, d) conventionally fabricated alloys composed of metastable phases, (e, f) additively manufactured alloys which contain metastable phases only owing to fast cooling. Dash lines in the plots outline the range of tensile strength or the bottom/upper limit of elongation that most experimental data are positioned.

Table 4 lists the tensile properties of Ti-Ta alloys along with their key processing parameters and corresponding phase constitution. Elastic admissible strain (EAS), indicative of the mechanical suitability of an implant material, is defined by [160].

(3) $$EAS=\frac{\sigma_{0.2}}{E}\times 100$$

where $\sigma_{0.2}$ and E are yield strength and elastic modulus, respectively. The EAS of Ti-Ta alloys is discussed in Section 4.5. For Ti-Ta alloys composed of stable phases, the strength initially increases with increasing Ta content, peaking at about 40-50%Ta, then decreasing, as indicated in Fig. 10a [35,[60], [61], [62],^147^,[161], [162], [163]]. The strength values mainly lie in 700-1200 MPa, varying with the alloy composition and processing history. In contrast, the ductility does not exhibit an evident dependence on the alloy composition (Fig. 10b [35,61,69,147,[161], [162], [163]]). The strength variation with Ta content is primarily a result of the changes in phase constitution and microstructure features.

**Fig. 11:**
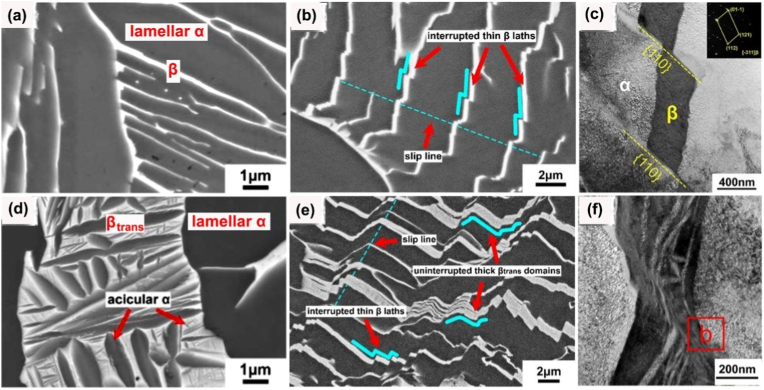
Microstructure comparison between (a-c) Ti-15Ta and (d-f) Ti-25Ta before and after tensile testing [61]. (a, d) SEM image before testing, (b, e) SEM image of an area near the fracture, (c, f) transmission electron microscopy (TEM) image of a single β lath near the fracture. Acicular α precipitates (in grey) are visible in thick β laths in (f).

• In Ti-Ta alloys with >50%Ta, the microstructure is usually dominated by equiaxed β grains dispersed with needle-like α precipitates in the matrix (Fig. 5c-d), where the α precipitates act as reinforcement; increasing Ta content reduces the fraction of α precipitates and consequently decreases the strength and hardness.
• A different mechanism exists for the mechanical property-microstructure relationship in α phase-dominated Ti-Ta alloys (with ≤40%Ta). Wang et al. [61] reported that such α-phase-dominated Ti-Ta alloys consist of lamellar α and β phases. As Ta content increased from 15% to 25%, the volume fraction of the β phase increased from 7.1% to 16.2%. At the same time, prior-β grain size decreased from 84.9 ± 22.0 μm to 75.2 ± 35.2 μm, and both grain boundary α and lamellar α reduced notably in width. These microstructure evolutions synthetically increased the tensile strength from 977 MPa to 1124 MPa. More interestingly, a closer examination revealed that a higher Ta content resulted in thicker β laths, which incurred the precipitation of acicular α in thick β laths. This microstructure notably changed the interaction between dislocations and α/β boundaries, hence the deformation mechanism. In Ti-15Ta, which contained thin β laths only (without acicular α precipitation), dislocations easily crossed α/β interface and cut through the β laths, forming interrupted β steps, as shown in Fig. 11a-c. In contrast, dislocations in Ti-25Ta were hindered at α/β boundaries by acicular α precipitates in thick β laths, leading to distorted but uninterrupted β domains (Fig. 11d-f). Thin β lath being cut through by dislocations was also reported in Ref. [63].

Tantalum is a weak β-stabilizer and slow diffuser, which dictates the structural arrangement and elemental redistribution during phase transformation. Thus, the cooling from T_β-tran_ influences the microstructure and resulting mechanical properties of Ti-Ta alloys. Stable phases of Ti-Ta alloys are formed by slow cooling at a rate of <10^−1^ °C/s. A decrease in the cooling rate enables a coarser microstructure and thus offers Ti-Ta a lower strength and ductility [164]. If accompanied by an increasing α phase fraction [124], the alloy's properties can be offset, since α phase has a higher strength but lower ductility than β phase. This can be inferred from Zhou et al.’s study [136]. Solution treatment and aging at 600/500 °C for 72 h provided Ti-50Ta with respectively 28 vol% and 58 vol% α phase in the β matrix; the former exhibited tensile properties σ = 714 MPa, σ_0.2_ = 612 MPa and δ = 11.6% while the latter exhibited σ = 1012 MPa, σ_0.2_ = 877 MPa and δ = 2.6%. A similar tensile property-phase constitution relation was reported in Ti-30/40Ta alloys [163].

#### 4.1.2 Alloys with metastable phases

Regarding Ti-Ta alloys consisting of metastable phases, the strength similarly increases with Ta content up to about 50%Ta. However, it fluctuates visibly at higher Ta contents [13,165]. As indicated in Table 4 and Fig. 10c [13,65,71,137,146,154,167,168], the ultimate tensile strength (σ) and yield strength (σ_0.2_) mostly fall in the range of 500-900 MPa and 350-600 MPa, respectively. Higher strengths up to 1570 MPa were achieved in alloys made by PM process [65], where the strengthening by impurities, to some extent, is inevitable [168]. Nevertheless, the overall strength is lower than that of the counterparts composed of stable phases (UTS: 700-1200 MPa in Fig. 10a). These alloys exhibit superior ductility to their counterparts (Fig. 10d) [13,65,71,137,146,154,167,168]. The tensile elongation (δ) increases up to 28% from 10% with increasing Ta content until ≤60%Ta; but it deteriorates notably at ≥70%Ta as the alloy enters the single β phase region. This declined ductility is unexpected because BCC crystals are known to have good ductility due to their abundance of slip systems.

Metastable Ti-Ta alloys undergo plastic deformation via dislocation slip, twinning and/or stress-induced martensitic transformation β → α'' (SIMT). The constituent phase seems to be a major factor in determining the mode activated for deformation. In an inhomogeneous microstructure, those three modes may occur synchronously. For example, Xu et al. [65] examined the microstructure of Ti-48.6Ta alloy before and after tension and observed the occurrence of slip and twinning in α′ laths of the Ta-rich zone, slip in β phase of Ti-rich zone, and SIMT plus slip in β phase of the diffusional zone; XRD further detected an increased fraction of α'' phase after deformation, corroborating SIMT mechanism. In another study by Xu et al. [147], they *in situ* observed SIMT in annealed Ti-48.6Ta during tension at ambient temperature using synchrotron radiation. The α'' from SIMT maintains an orientation relationship with its parent β phase, i.e., *a* axis of the orthorhombic α'' being parallel to an axis of the cubic β while *b* and *c* axes being parallel to Ref. [68] planes of β phase [138]. To accomplish the transformation, the axis of the cubic lattice in *a*, *b*, and *c* directions needs to undergo either elongation or contraction of a small percentage.

The ω phase has a high elastic modulus but is intrinsically brittle. Therefore, its precipitation in the microstructure will offer Ti-Ta alloys a higher modulus, strength, and hardness, but dramatically compromises ductility. Apart from acting as an obstacle to dislocation slip, the ω phase can also suppress the SIMT process. Margevicius and Cotton [138] measured the threshold stress for SIMT in Ti-60Ta, which increased with the fraction of ω phase. Nano-sized ω precipitates were observed in the untransformed β and transformed α'' phase. They concluded that these precipitates caused local strengthening in either β or α'' phase and, as a result, mutually impeded the β → α'' transformation. It is worth noting that SIMT is reversible after the load is released. However, the reverse transformation is subjected to factors including α''/β interface friction, elastic misfit of phases, and intense dislocations, etc. [137].

Varying cooling rates between 10° °C/s and 10^3^ °C/s enables various phases in different fractions with respect to alloy compositions, further tailoring the mechanical properties of the resulting Ti-Ta alloys. When cooling from α/β region, primary α phase exists in the microstructure along with the transformed α/α'/α'' phase and/or retained β. Faster cooling tends to enhance the β to α/α'/α'' transformation, changing the microstructures and tensile properties. For instance, air cooling Ti-40Ta from the α/β field produced discontinuous α lamellae in the β matrix, delivering tensile properties σ = 1069 MPa, σ_0.2_ = 786 MPa and δ = 2%; in contrast, water quenching from the field generated a microstructure comprising residual β, newly formed α'' platelets and trace primary α, offering tensile properties σ = 814 MPa, σ_0.2_ = 469 MPa and δ = 20% [35]. It is worth mentioning that existing α acts as nucleation sites preferentially for β to α transition, making the β to α'/α'' transformation possible only by fast cooling. This is supported that argon quenching of Ti-40Ta formed β, α and few α'' phase. Cooling from β field at 10° - 10^3^ °C/s produces metastable phases, but the effect of cooling rate remains unexplored so far. Studies of analogous materials may give a clue. Afonso et al. [169] observed that the dominant α'' phase increased in volume fraction but decreased in size, as Ti-20Nb alloy cooled from T_β-tran_ at a rate of 4.6-139.6 °C/s. Their other study of Ti-40Nb presented that an increase in cooling rate from 5 °C/s to 50 °C/s led to an increased fraction of the dominant β phase and accordingly decreased hardness and modulus of the alloy [170].

### 4.2 Additively manufactured dense alloys

Table 5 summarizes the tensile properties of as-built Ti-Ta alloys prepared by AM process (only PBF-LB/M data available). Most compositions exhibit a tensile strength between 750 MPa and 1100 MPa and an elongation of around 10%, as shown in Fig. 10e and f [[83], [84], [85], [86], [87], [88],^90^,^91^,^105^,^117^,^171^]. Compared to the counterparts fabricated conventionally (UTS: 500-900 MPa in Fig. 10c; elongation: >10% in Fig. 10d), AM Ti-Ta alloys of the same composition exhibit higher strengths but lower ductility. This may result from the fine microstructure, residual porosity and unmelted Ta in AM alloys. The effect of unmelted Ta on the strength is supposed to be insignificant [106] or even detrimental [82]. Gao et al. [82] observed that the sample containing the most unmelted Ta (10.3 vol%) and the fewest pores (0.02% porosity) exhibited the lowest tensile strength; Ta particles in areas near the fracture were severely elongated without cracking or detaching from the matrix. Therefore, the authors concluded that unmelted Ta particles adversely affected the strength of Ti-Ta alloys. Despite disregarding the difference in phase constitution, their interpretation is probably reasonable when taking into account the weak Ta-matrix bond and the soft and ductile nature of pure Ta (σ = 170-480 MPa, σ_0.2_ = 140-345 MPa and δ ≥ 25%, ISO 13782 [172]). This means that malleable Ta imparts a great capacity for deformation, allowing unmelted Ta particles to deform synchronously with the matrix without developing into a stress concentrator.

The cooling rate of AM fabrication varies with the process, which is a likely factor behind the different microstructure and mechanical properties of the as-built Ti-Ta alloys. PBF-EB/M employs a high substrate temperature, e.g., 540 °C [101], thus offering a slow cooling rate. Moreover, the substrate temperature is close to or even higher than T_β-tran_ of Ti-Ta alloys, and thus the as-built alloys consist of stable phases, and the microstructure is relatively coarse. For example, PBF-EB/M Ti-61.8Ta exhibited prior-β grains averaging 19.5 μm [101], whereas prior-β grains in PBF-LB/M Ti-61.8Ta printed from the same powder feedstock were 5.0 ± 3.0 μm [104] (Fig. 7c and d). DED-LB/M and PBF-LB/M are featured by fast cooling, respectively >10^3^ °C/s and >10^4^ °C/s, providing Ti-Ta alloys with metastable phases. The faster cooling with PBF-LB/M process enables finer cells and dendrites as compared to DED-LB/M parts, e.g., 1-3 μm vs. 2-10 μm cells in Ti-50Ta [92,125]. Finer microstructures with PBF-LB/M impart Ti-Ta alloys a higher strength but lower ductility. Take Ti-10Ta for example, DED-LB/M delivered tensile properties σ = 735 MPa, σ_0.2_ = 642 MPa and δ = 16.4% [127], while PBF-LB/M offered σ = 780-1020 MPa, σ_0.2_ = 730-885 MPa and δ = 8.7-10.5% [84,117]. In addition, the cooling rate of AM process is partly determined by the energy density; Han et al. [173] reported that the cooling rate in PBF-LB/M process decreased from 7.2 × 10^6^ °C/s to 2.9 × 10^6^ °C/s with the energy density increasing from 42.735 J/mm^3^ to 177.78 J/mm^3^. Varying energy densities leads to notable changes in the microstructure and mechanical properties, as evidenced in Refs. [82,83,89,90]. Nevertheless, these changes are a synergistic result of variations in melting pool size, melt temperature, and temperature gradient, all of which are associated with the energy density.

### 4.3 Additively manufactured lattices

Table 6 summarizes the effective compressive properties of Ti-Ta lattices fabricated by AM. Properties of human tibia and femur bones under compression are included for comparison. Two significant observations can be drawn from Table 6:

• The mechanical properties of Ti-Ta lattices are strongly dependent on their topology. Take Ti-25Ta as an example, Schwarz lattices with a relative density of 49.9 ± 0.9% show notably superior modulus (18.9 ± 2.0 GPa) and compressive yield strength (102 ± 9 MPa) to that of TPMS-based gyroid (10.4 ± 1.5 GPa, 74 ± 4 MPa) at a similar relative density (48.5 ± 1.3%).
• Some Ti-Ta lattices match human bones in terms of elasticity and compressive strength. For example, Ti-30Ta stochastic lattices presented *E* = 0.5-1.0 GPa, $\sigma_{0.2}$ = 6.3-12.3 MPa and *EAS* (1.23-1.26) at 89-92% porosity, all overlapping with the corresponding range of cancellous tibia/femur bones (*E* = 0.02-0.83 GPa, *σ* = 2.2-10.6 MPa, *EAS* = 1.2-13). Similarly, the Ti-25Ta strut-based diamond lattice with a designed porosity of 85% largely matches cancellous bones. Regarding cortical bones, their properties (*E* = 15.4-35.3 GPa, *σ* = 179-213 MPa, and *EAS* = 0.6-1.2) are close to those of Ti-25Ta TPMS-based diamond lattice (*E* = 15.8 ± 0.5 GPa, $\sigma_{0.2}$ = 236 ± 6 MPa, and *EAS* = 0.8) at 30.7% porosity.

**Fig. 12:**
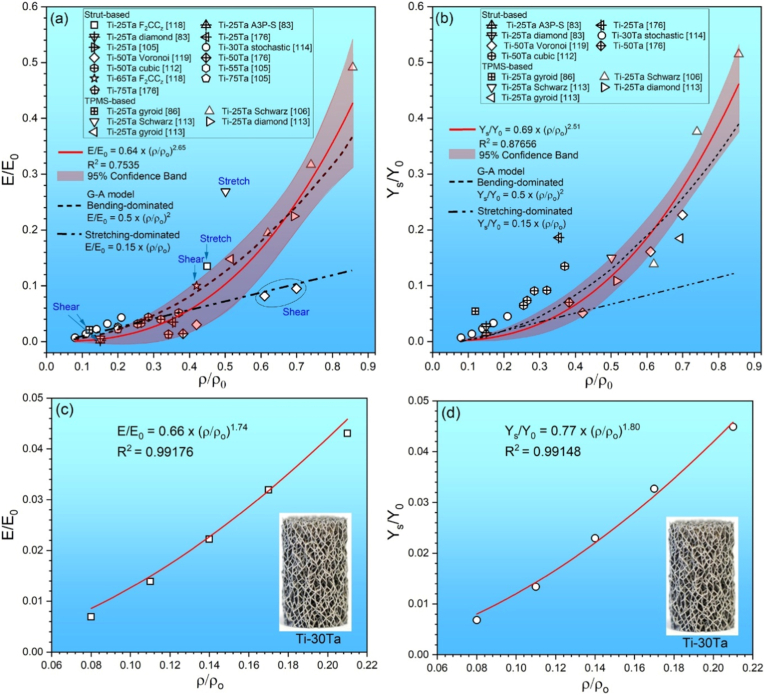
Variation of (a) relative elastic modulus and (b) relative compressive yield strength of Ti-Ta lattice structures with their relative density. Gibson-Ashby model for respectively bending-/stretch-dominated deformation was superimposed for comparison. Most lattices likely deform in a bending-dominated deformation mode except th0se indicated as stretching-/shear-dominated mode. (c) The relative modulus and (d) relative yield strength of Ti-30Ta stochastic lattice relative to the density, which are well fitted by the Gibson-Ashby model with high R^2^ values (>0.99) [114]. The elastic modulus and yield strength of dense Ti-30Ta, Ti-50Ta and Ti-75Ta from PBF-LB/M are cited from Refs. [81,105,117]. The effective elastic moduli of Ti-Ta lattices were listed in Table 6.

The effective elastic modulus and yield strength of porous structures decrease with the increase of their porosities, which can be described by the Gibson-Ashby (G-A) model as follows [174]:

(4) $$\frac{E}{E_{0}}=C_{E}{\left(\frac{\rho }{\rho_{0}}\right)}^{k_{E}}=C_{E}{\left(1-\rho_{r}\right)}^{k_{E}}$$

(5) $$\frac{Y_{S}}{Y_{0}}=C_{Y}{\left(\frac{\rho }{\rho_{0}}\right)}^{k_{Y}}=C_{Y}{\left(1-\rho_{r}\right)}^{k_{Y}}$$

where $E_{0}$, $Y_{0}$, and $\rho_{0}$ are elastic modulus, yield strength, and density of dense materials, respectively; ρ and $\rho_{r}$ are the density and porosity of porous materials; $C_{E}$, $k_{E}$, $C_{Y}$, and $k_{Y}$ are constants associated with structure features. Fig. 12a and b shows relative elastic modulus vs. relative density and relative compressive yield strength vs. relative density in Ti-Ta lattices with corresponding fitting curves. Nevertheless, factors such as lattice topology and data inaccuracy slightly scatter the experimental data particularly in yield strength. Re-plotting the data from Ti-30Ta stochastic lattices presents a better fit into the G-A model with R^2^ > 0.99, as shown in Fig. 12c and d. This can be ascribed to their low relative densities (≤22%) and high strut length-to-thickness ratios (slenderness ≫5), which perfectly meet the applicability criteria of the G-A model to metal lattice materials [175].

Over a wide porosity range, Figs. 12a and b indicate a positive power relationship with exponents slightly above 2.5 in the relative elastic modulus or yield strength vs. relative density [83,86,105,106,[112], [113], [114],^118^,^119^,^176^]. Most elastic modulus data fall in the 95% confidence band regardless of the porosity level and lattice topology, whereas the yield strength data scatter appreciably because the strength is sensitive to not only the constituent phases and their fractions but also their morphologies and sizes. As for the elastic modulus, the fitting curve presents an exponent 2.65, which is close to the G-A model value 2 for bending-dominated deformation mode but far higher than 1 for stretching-dominated mode [14]. This agrees with that the deformation of many Ti-Ta lattices proceeds dominantly via bending. Deformation dominated by stretching or shear reportedly occurs in some Ti-Ta lattices, indicated in Fig. 12a. A clear correlation between the lattice topology/porosity and the deformation mechanism cannot be defined from the limited experimental evidence. In general, the deformation behavior of Ti-Ta lattices in response to compressive stress is associated with three major factors: i) lattice topology, which determines the stress distribution between the lattice struts. Take Ti-25Ta for instance, the deformation of a TPMS-based Schwarz structure was stretching-dominated while that of a TPMS-based gyroid structure was bending-dominated, given their similarities in the phase and porosity [113]; ii) the porosity, which reflects the slenderness of lattice struts. Ti-50Ta of a Voronoi tessellation structure deformed in a bending-dominated mode at 58% porosity but deformed in a shear-dominated mode at 30/39% porosity [95,119]; and iii) composition and constituent phase, which dictates the material ductility. Soro et al. [118] reported that Ti-25Ta and Ti-65Ta lattcies, although the identical lattice unit (F_2_CC_z_) and porosity (55% vs. 58%), respectively exhibited stretching-dominated and shear-dominated deformation modes because of their differences in constituent phases (α′ vs. β). Fig. 12 proves that G-A model is applicable to cellular structures that have high porosities with strut slenderness greater than 5, like Ti-30Ta stochastic lattices [114]. While for most Ti-Ta strut-based lattices, a coupled mechanical property model comprising bending, stretching, and shear deformation mechanisms, proposed by Zhong et al. [175], seems to be more applicable.

### 4.4 Fatigue properties

The fatigue performance of Ti-Ta alloys is critical for their orthopedic applications in load-bearing areas, as such implants are subjected to dynamic forces from daily life activities. Table 7 summarises the fatigue properties of Ti-Ta alloys (both bulk and lattices) fabricated by conventional and AM processes.

**Table 7:** Fatigue properties of Ti-Ta alloys.

Ti-xTa (x: wt.%)	Processing	Topology	Porosity (%)	Phases	Gauge size (mm)	Frequency (Hz)	Load	R	σ_f_ (MPa)					Ref.
									10^3^	10^4^	10^5^	10^6^	10^7^	
50	SPS, HR	Bulk	0	α-Ti+β-Ta+α''	59.25 × 20 × 3	25	Tension	0.1	–d	–	720	540	440	[71]
52^a^	Melting, ST (890 °C), ageing (500 °C)	Bulk	0	NA^c^	Ø7 × 7.66	10	Torsion	−1	–	∼300	∼185	∼130	80	[177,178]
	Melting, ST (700 °C), ageing (500 °C)								–	∼270	∼175	∼120	50	
	Melting, ageing (500 °C)			NA					–	–	∼320	∼180	70	
	Melting, ST (890 °C), ageing (500 °C)			NA	3 × 2 (R30)		Bending	0	–	660	∼380	∼240	220	
	Melting, ST (700 °C), ageing (500 °C)			NA					–	500	300	∼250	280	
	Melting, ageing (500 °C)			NA					–	400	∼370	340	300	
25	PBF-LB/M	Bulk	0.15 (S^b^)	α'+Ta	8 × 3 × 1.5	–	Push-pull	−1	–	490	320	157^e^	–	[159]
			0.37 (R^b^)						654	422	–	120^e^	–	
65	PBF-LB/M	Bulk	0.12 (S)	β+Ta	8 × 3 × 1.5	–	Push-pull	−1	564	463	–	130^e^	–	[88]
			0.03 (R)						691	498	–	210^e^	–	
25	PBF-LB/M	F_2_CC_Z_	55	α'+Ta	10 × 10 × 10	–	Push-pull	−1	∼260	∼150	∼80	∼50	–	[118]
65			58	β+Ta					∼350	∼170	∼80	∼40	–	
30	PBF-LB/M	Stochastic	79	α''+Ta	Ø13 × 21	15	Compression	0.1	–	–	–	8.3 ± 0.4	–	[114]
			83						–	–	–	6.0 ± 0.3	–	
			86						–	–	–	4.2 ± 0.2	–	
			89						–	–	–	2.6 ± 0.1	–	
			92						–	–	–	1.3 ± 0.1	–	
50	PBF-LB/M	Voronoi tessellation	30	β+ α''+Ta	Ø5 × 10	20	Compression	0.1	–	230	200	140	–	[119]
			39	α''+Ta					–	–	160	105	–	
			58.6	α/α'+Ta					–	–	38	27	–	
Cancellous bone (tibia)	NA	NA	NA	NA	0.07-0.14 thick	2	Bending	0	140	125	113	100	–	[182]
Cortical bone (tibia)	NA	NA	NA	NA	0.07-0.14 thick	2	Bending	0	170	165	155	160	–	[182]
Cortical bone (femur)	NA	NA	3.5-19.0	NA	25 × 5 × 3	2	Compression		88	75	60	47	–	[181]

a The actual alloy composition was Ti-52Ta-4Sn.

b S: single melt applied to the fabrication; R: remelt applied to the fabrication.

c NA: not presented or not applicable.

d –: data not stated.

e Extrapolated value.

#### 4.4.1 Fatigue properties of bulk Ti-Ta alloys

Experimental data from the limited number of studies shows that Ti-Ta alloys possess a fatigue limit from 50 MPa to 440 MPa at 10^7^ cycles [71,177,178]. The best fatigue strength – 440 MPa measured from SPSed and hot rolled Ti-50Ta, is superior to that of coarse-grained pure Ti (210 MPa, grade 2) and comparable to that of ultrafine-grained pure Ti (400-490 MPa) [179] and wrought Ti-6Al-4V (around 400 MPa) [180]. This outstanding value is ascribed to the alloy's unique microstructure, featured by alternating Ti and Ta lamellae and thin diffusion layers at their interface.

In contrast, bulk Ti-Ta alloys built by PBF-LB/M exhibited an inferior fatigue limit – maximum 343 MPa at 4.7 × 10^4^ cycles in Ti-25Ta and 463 MPa at 10^4^ cycles in Ti-65Ta, when tested at a strain amplitude of ±0.5% [88,159]. Remelt scanning slightly improved Ti-65Ta (498 MPa at 10^4^ cycles) but worsened Ti-25Ta (372 MPa at merely over 10^4^ cycles). All specimens were machined from build blocks and ground with 1000-grit paper, precluding the effect of as-built rough surface. For both compositions, cracks initiated at a single surface site and propagated nearly across the entire fracture surface when tested at a low strain amplitude (±0.5%); however, testing at a high strain amplitude (±0.8%) induced multiple crack sites, resulting in smaller crack propagation zones. Under all conditions examined, pores in the direct vicinity of the specimen surface acted as the crack initiation site, whereas unmelted Ta particles remained coherent and made no contribution to the crack initiation and propagation. This is largely derived from the multiple slip systems of BCC Ta and its malleability, which substantially preclude dislocations from accumulating at the Ta-matrix boundary and hence facilitate a coherent Ta particle-matrix deformation.

#### 4.4.2 Fatigue properties of Ti-Ta lattices

Ti-Ta lattices display promising fatigue performances under high- and low-cyclic loading conditions in few studies. Ghouse et al. [114] investigated the high-cycle fatigue property of Ti-30Ta stochastic lattices with compression-compression loading at R = 0.1 and a frequency of 15 Hz. The measured fatigue strength increased from 1.3 ± 0.1 MPa to 8.3 ± 0.4 MPa at 10^6^ cycles as the relative density increased from 8% to 21%. Such strength values are far lower than those of cortical (tibia/femur) and cancellous (tibia) bones [181,182], but comparable to or even better than those of Ti-6Al-4V (1.5 ± 0.1 MPa to 7.4 ± 0.4 MPa) and pure Ti (1.3 ± 0.1 MPa to 8.5 ± 0.4 MPa) stochastic structures fabricated under similar conditions. A recent study by Liu et al. [119] realized a dramatic improvement from Ti-50Ta Voronoi tessellation-based lattices with lower porosities, achieving a fatigue strength of 100 MPa at 39% porosity and 140 MPa at 30% porosity at 10^6^ cycles, outperforming pure Ti (53 MPa at 43% porosity) and pure Ta lattices (36 MPa at 56% porosity). Decreasing the porosity is efficient to improve the fatigue properties of Ti-Ta lattices but at the cost of increasing effective elastic modulus.

In the low-cycle fatigue regime, Ti-50Ta Voronoi tessellation-based lattices also exhibited an advantageous fatigue behavior over pure Ta and Ti lattices at 10^5^ cycles, respectively 200 MPa in Ti-50Ta (30% porosity), 160 MPa in Ti (43% porosity) and 38 MPa in Ta (58.6% porosity) [119]. Ti-25Ta and Ti-65Ta lattices in F_2_CCz structure were examined under tension-compression loading at R = −1; a fatigue life of approximately 5 × 10^5^ cycles at 50 MPa, 3 × 10^4^ cycles at 120 MPa, and 5 × 10^2^ cycles at 300 MPa was measured in Ti-25Ta, and correspondingly about 5 × 10^5^ cycles, 3 × 10^4^ cycles, and 2 × 10^3^ cycles were obtained in Ti-65Ta at these loads [118]. Both are much better than Ti-6Al-4V, which is just over 10^4^ cycles in the as-built state or 10^5^ cycles in the heat-treated state when tested at the same stress amplitude [183]. The inferior fatigue performance of Ti-6Al-4V is supposed to result from its lower ductility, 1.9-2.3% tensile elongation to fracture vs. 3.54-5.89% tested in Ti-(25, 65)Ta. The underlying principle is that low-cycle fatigue failure is dominated by crack propagation, which strongly depends on the material ductility [184].

### 4.5 Elastic modulus and elastic admissible strain

**Fig. 13:**
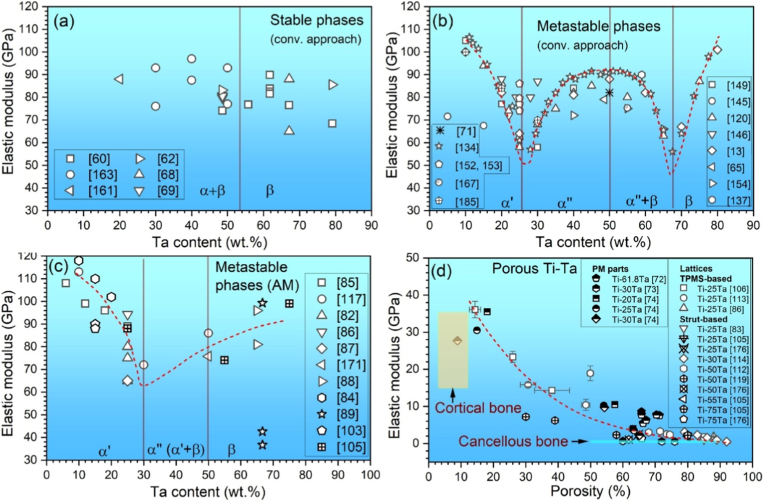
Elastic modulus as a function of Ta content in dense Ti-Ta alloys, consisting of (a) stable phases fabricated by conventional approaches after slow cooling, (b) metastable phases by conventional approaches after moderate or rapid cooling, (c) metastable phases by AM process. (d) Effective elastic modulus of porous Ti-Ta including lattices and PM parts as a function of porosity. Moduli measured by the indentation technique are not included in the plot.

Elastic modulus is a key indicator of mechanical biocompatibility for a candidate biomaterial for orthopedic applications [185]. Like other materials, the elastic modulus of Ti-Ta alloys is essentially determined by their phase constitution and porosity. Fig. 13a-c plot the elastic modulus varying with Ta content in Ti-Ta alloys consisting of stable and metastable phases from conventional approaches and AM processes [13,60,62,71,[84], [85], [86], [87], [88], [89],^103^,^117^,^120^,^134^,^137^,^145^,^146^,^149^,^152^,^153^,^161^,^163^,^167^,^171^,^185^]. The data from the alloys with stable phases is limited. No clear trend can be concluded between the elastic modulus and Ta content (Fig. 13a), probably because other factors like porosities mask their relationship [60]. In contrast, alloys with metastable phases by conventional approaches exhibit an interesting “W-shaped” elastic modulus-Ta content relationship (Fig. 13b). Two minimum values, respectively 58 GPa at 25%Ta and 61 GPa at 70%Ta, are present within 10% to 80% Ta content range examined hitherto. They appear at the phase transformation boundary from α′ to α'' and from α''+β to β (refer to session 3.3 and Table 3) [186]. Among the metastable phases in Ti-Ta alloys, ω phase reportedly has the highest elastic modulus, followed by α′, α'' and then β phase. The mixture rule can predict the modulus of Ti-Ta alloys. AM Ti-Ta alloy almost presents an identical modulus-Ta content variation curve (Fig. 13c) to the one observed in conventionally prepared alloys (Fig. 13b) within 6-65%Ta range. If excluding the abnormal data (36.70 ± 2.11 GPa or 42.57 ± 1.32 GPa in Ti-66.7Ta) reported by Zhang et al. [89], only one minimum – 65 GPa at about 25%Ta, is obtained. This similarity between AM and conventionally rapidly cooled Ti-Ta alloys is reasonable, considering that their phases similarly depend on the composition and cooling rate. It is noteworthy that, provided with the same composition, AM Ti-Ta exhibits a higher modulus than the conventional alloy (Fig. 13b and c), which is supposed to derive from the unmelted Ta in the build.

Fig. 13d depicts the effective elastic modulus of porous Ti-Ta alloys as a function of the porosity with respect to the material fabrication strategy and lattice topology [[72], [73], [74],^83^,^86^,^106^,[112], [113], [114],^119^,^176^]. The elastic modulus decreases monotonically with the increase of alloy porosity, indicating that fabricating porous structures is an efficient and viable strategy to relegate the elastic modulus of Ti-Ta alloys. In limited cases, porous Ti-Ta alloys present promising elastic moduli close to that of cancellous or cortical bones, for example, Ti-30Ta stochastic lattices at 89-92% porosity (*E* = 0.5-1.0 GPa) and Ti-25Ta TPMS-based diamond lattices at 30.7% porosity (*E* = 15.8 ± 0.5 GPa) [106,113,114]. The porosity level is crucial to the design of porous Ti-Ta alloys, which must meet the elasticity requirements for orthopedic implant applications. It can be roughly predicted from the G-A model if the strut length to width ratio is > 5 [174], or from stretching-bending-shear analytical model if the ratio is < 5 [175]. Other factors, such as fabrication process, lattice topology and alloy composition, make secondary contributions to the elasticity variations because materials’ elasticity is relatively sensitive to the percentage of microstructure features rather than their morphology and dimension. But, these factors are influential for other properties, such as biological behavior, particularly (see Section 7). For instance, porous parts from PM process usually contain isolated pores, which offer inferior permeability for blood flow, nutrient delivery and metabolic waste removal.

**Fig. 14:**
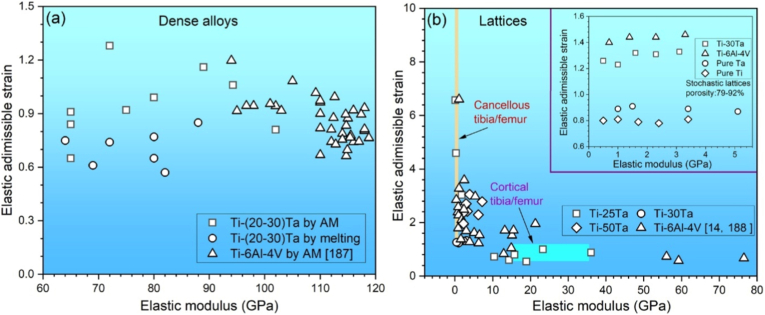
Elastic admissible strain of Ti-Ta alloys as a function of effective elastic modulus, plotted from data in Table 4, Table 5, Table 6 (a) Dense Ti-(20-30)Ta compared to dense Ti-6Al-4V fabricated by powder bed fusion [187], (b) Ti-Ta lattices vs. Ti-6Al-4V lattices [14,188], and the inset focusing on stochastic lattices of Ti-30Ta, Ti-6Al-4V, pure Ti and pure Ta [114].

Elastic admissible strain (EAS) is another critical parameter that determines the suitability of biomaterials for applications in load-bearing areas. It is a measure of the maximum extent to which a biomedical material undergoes elastic deformation. Load-bearing implants, such as joint replacements and fracture fixation devices, withstand the mechanical stress of daily life activities, which requires a high yield strength to fulfil their function. Meanwhile, such implants are responsible for transferring dynamic loads from the implant to the host bone. To prevent the stress shielding effect, an elastic modulus that can match that of human bones is imperative. In this regard, a high EAS is strongly demanded in the design and development of biomaterials for load-bearing implants. Fig. 14a compares the EAS between dense Ti-Ta alloys and Ti-6Al-4V – the most widely used biomaterial in such areas [187]. For simplicity, Ta content is limited to 20-30% because this composition range offers the lowest elastic moduli (Fig. 13b). It is evident that most Ti-(20-30)Ta alloys exhibit an EAS varying from 0.6 to 1.2, essentially comparable to that of Ti-6Al-4V; AM Ti-Ta alloys have a slightly higher EAS than their counterparts made by melting, probably because of their finer microstructures and thereby higher yield strengths. Regarding Ti-Ta lattices, there is no clear dependence between their EAS and porosity level or lattice structure. Contrary to dense Ti-Ta, the EAS of Ti-Ta lattices decreases with the increase of effective elastic modulus and roughly splits into two groups (Fig. 14b [187]). In the group with elastic moduli <4 GPa, Ti-Ta lattices usually have an EAS between 1.2 and 3, falling in the EAS range of human cancellous tibia/femur bones (EAS≥1.2). In contrast, in the other group with elastic moduli of 10-35 GPa, Ti-Ta lattices have an EAS of 0.54-1.0, barely meeting the requirement of cortical tibia/femur bones (EAS = 0.6-1.2). Compared to Ti-6Al-4V lattices, Ti-Ta lattices exhibit a lower EAS. However, they are superior to pure Ti and Ta lattices, provided they all have the same topology and close porosity levels [114].

## 5 Surface modification of Ti-Ta alloys to enhance bioactivity

The surface (composition and topography) of the manufactured Ti-Ta alloys plays important roles in the corrosion resistance and biocompatibility of the alloy, essential for its biomedical applications, especially in regulating protein adsorption and determining subsequent cell responses, including cell attachment, proliferation, migration and differentiation [46,[189], [190], [191], [192]].

The excellent biocompatibility of Ti-Ta alloys is mainly due to the naturally occurring nanometre-thick oxide layer (e.g., TiO_2_ and Ta_2_O_5_) on the surface of the alloy [28,193,194]. Such an oxide layer makes the alloy inert, preventing it from reacting with the body's tissues or fluids. This reduces the risk of adverse reactions, such as inflammation or rejection, when implanted in the body [195,196]. Also, the oxide layer can enhance the alloy's corrosion resistance by acting as a barrier against corrosive body fluids (see discussion in Section 6), impeding the release of metal ions into surrounding tissue and ensuring long-term implant stability [44,85]. As a result, various oxide-forming methods have attracted extensive interest in designing and manipulating such biocompatible oxide layers on the surface of Ti-Ta alloys. The surface topography of Ti-Ta implant materials, specifically its roughness and texture, plays a crucial role in the success of osseointegration, the interlocking process at the bone-implant interface. Different levels of topography, including macro-, micro-, and nano-topography, influence bone formation, cell adhesion, and ultimately, the implant's stability and longevity [189]. This section elaborates on the surface modification techniques that have been explored for Ti-Ta alloys. It should be noted that the microstructure underlying the surface may also be altered, particularly after treatment at high temperatures, such as liquid metal dealloying. Such internal microstructure changes have little effect on the bioactivity of Ti-Ta implants and, therefore, are not addressed in this review.

### 5.1 Anodization

**Fig. 15:**
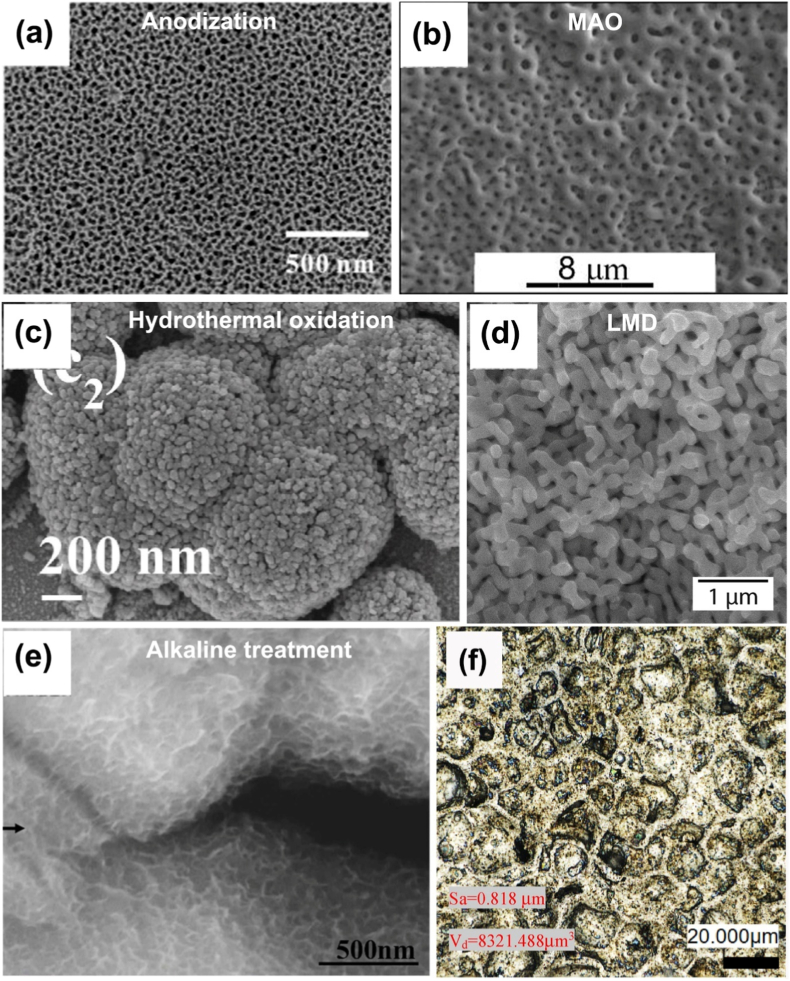
Surface morphology of Ti-Ta alloys after modification: (a) self-ordered nanotube coating on Ti-50Ta anodized in 1 M H_2_SO_4_ + 0.15 wt% HF [197]; (b) porous oxide layer on Ti-25Ta processed by MAO in an electrolyte consisting of 0.35 M (CH_3_COO)_2_Ca·H_2_O, 0.02 M C_3_H_7_Na_2_O_6_P·5H_2_O and 0.1 M (CH_3_COO)_2_Mg·4H_2_O, by a DC power source at 250 V, 2.5 A, at room temperature for 60 s [205]; (c) cauliflower-like morphology on Ti-60Ta plate after hydrothermally treated in 30 mL H_2_O_2_ solution (30 wt%) at 140 °C for 24 h [209]; (d) Ti-Ta dealloyed in Cu_32_Ag_68_ medium (T_m_ ∼780 °C) bath for 1 h [212]; (e) Ti-15Ta after alkaline heat treatment forming a nanoscale porous surface [76]; (f) Ti-Ta processed by ultrasonic cavitation and particle micro-abrasion exhibiting circular pits and a toughened surface [221].

Anodization is an effective way to grow TiO_2_-Ta_2_O_5_ nanotube arrays on Ti-Ta substrates [150,195,[197], [198], [199], [200], [201]], as shown in Fig. 15a [195]. By modifying the voltage, electrolyte, and anodization time, nanotubes with diameters ranging from 25 to 120 nm and lengths of approximately 1 to 60 μm can be created [195,[197], [198], [199], [200], [201]], without altering the microstructure of the underlying Ti-Ta alloys [198]. Aside from the anodization processing parameters, the composition and phase constitution of the Ti-Ta alloys are important for forming TiO_2_-Ta_2_O_5_ nanotubes. For example, Lee et al. [150] anodized arc-melted Ti-(10, 20, 30, 40)Ta alloys in 1.0 M H_3_PO_4_ + 0.8 wt%NaF electrolyte at a potential of 10 V for 2 h, and observed that with Ta content increasing from 10% to 40% the length of TiO_2_-Ta_2_O_5_ nanotubes increased from about 3.0 μm to 4.0 μm, and meanwhile their diameters became less distinct. Tsuchiya et al. [197] examined Ti-(13, 25, 50, 80)Ta alloys by anodizing the materials at a potential of 20 V for several hours in 1 M H_2_SO_4_ + 0.15 wt%HF electrolyte. The surface of all four alloys was covered with amorphous self-ordered TiO_2_-Ta_2_O_5_ nanotubes with diameters of 30-120 nm. However, only the Ti-80Ta alloy yielded uniformly homogeneous nanotube arrays (surface coverage and tube length). At the same time, the Ti-13Ta and Ti-25Ta showed an uneven appearance, and the Ti-50Ta exhibited unequal tube lengths [197]. Phase constitution of Ti-Ta alloys obtained by conventional methods in Table 3, Section 3.3 indicates Ti-80Ta consists of a single β phase, while Ti-13, 25, and 50Ta comprise α+β phases. Tsuchiya et al.’s observation [197] reflects that single β phase supports more evenly grown nanotubes on the surface of the Ti-Ta alloys. In contrast, α+β phase microstructures exhibit disrupted tube formation.

In general, TiO_2_-Ta_2_O_5_ nanotubes from anodization approach are amorphous, which is stable up to 700 °C [199]. Post heat treatment at temperatures starting from 850 °C leads to the formation of crystalline TiTa_2_O_7_, Ta_2_O_5_, and TiO_2_ phases [199]. It has been reported that crystalline TiO_2_ exhibits higher chemical stability [199] and corrosion resistance than amorphous TiO_2_ [198], which suggests that the crystalline oxide nanotubes after heat treatment may offer better biological responses. At a temperature of 1000 °C, the nanotube walls become visibly rougher with the oxide grain size growing from 18.01 nm at 800 °C to 35.60 nm, exceeding the original nanotube wall thickness of 23.11 nm. Due to such grain size increases, heat treatment at temperatures above 1000 °C results in a total collapse of the nanotube structure. It should be noted that anodization generally yields weak adhesion between the TiO_2_-Ta_2_O_5_ nanotubes and the Ti-Ta alloy substrate without a transition zone in between [[202], [203], [204]]. Although anodized Ti-Ta alloys exhibit wettability and biological properties superior to their counterparts without anodization treatment (Sections 6.1, 7.3), this weak adhesion makes long-term biomedical applications of such surfaces challenging or impossible.

### 5.2 Micro arc oxidation (MAO)

MAO process transforms Ti-Ta surface into a strongly adherent and porous TiO_2_-Ta_2_O_5_ layer. The pore size varies from ∼0.1 μm to ∼10 μm, and the oxide layer thickness ranges from 6.2 μm to 9.6 μm (Fig. 15b), by adjusting MAO parameters such as current, voltage, and electrolytes [196,205,206]. For instance, Kuroda et al. [206] observed that with the increase of current from 1 A to 2 A, the bioceramic layer thickness increased from 6.2 ± 1.3 μm to 9.6 ± 2.7 μm. In comparison, with a further increase of current to 2.5 A, the thickness started to reduce (9.1 ± 1.6 μm). The influence of current on pore morphology and size is negligible – similar roundish pores ranging from ∼0.1 μm to ∼10 μm were created from all current conditions. The current also influenced both the crystallinity and the rutile-to-anatase ratio of the TiO_2_ crystal structure. The crystallinity of TiO_2_ decreased from ∼45% (1A) to ∼30% (1.5-2.5 A) while the rutile-to-anatase ratio increased from 1:4 (1A) to 3:10 (1.5-2.5 A) [206]. These effects are significant as it has been reported that TiO_2_ rutile crystalline on pure Ti can offer superior corrosion resistance compared to anatase crystalline and amorphous TiO_2_ [207], which can also be applied to Ti-Ta alloys. MAO coatings exhibit superior adhesion compared to anodized nanotube layers, which is achieved by a thin gradient metal-oxide transition zone between the oxide layer and Ti-Ta alloy [196,208]. This makes MAO a more reliable choice for long-term biomedical applications. MAO treatment substantially roughens the surface, enhancing the wettability and bioactivity of Ti-Ta alloys (see Sections 6.1, 7.3).

### 5.3 Hydrothermal oxidation

Proposed by Liao et al. [209], Ti-Ta alloys were immersed in pre-heated H_2_O_2_ (30 wt%, 140 °C) and isothermally held for 24 h, which enabled a micro/nano hierarchical surface, as shown in Fig. 15c. The nanoparticles of 20-40 nm in size, arranging in a coagulated, cauliflower-like structure on Ti-60Ta substrate, were identified to be anatase TiO_2_ and amorphous Ta_2_O_5_, while the bulk microstructure of the underlying Ti-Ta remains unchanged. The oxide surface layer is probably bonded firmly to the substrate through a thin gradient metal-oxide transition zone, inferred from a similar study on pure Ti [210]. Such hierarchical surface features offer superhydrophilic wettability to water with a contact angle approaching zero [209]. Furthermore, subsequent precalcification in saturated CaCl_2_ and K_2_HPO_4_ solutions to deposit bioactive Ca^2+^ and HPO_4_^2−^ ions in the surface layer remarkably promotes the in-body apatite formation, making the surface entirely covered by apatite after immersing in SBF for only 1 day.

### 5.4 Liquid metal dealloying (LMD)

LMD exploits the different solubilities of alloy components in molten metals (medium), in which the miscible component is extracted from the alloy while the immiscible element self-organizes into a 3-dimensional porous network filled with the medium. After removing the medium, generally by etching in acid, interconnected ligaments of the immiscible element are thus exposed, forming a nano-/micro-scale porous structure [211]. LMD of Ti-Ta alloys has been intensively investigated with the use of Cu, GuAg or CuAgBi as the dealloying medium, where Ti is dissolved away at a rate varying with the medium composition, dealloying temperature and time, and precursor alloy composition [[212], [213], [214], [215]]. Accordingly, the dealloyed structure topology, dealloying depth, ligament thickness, and pore size can be subtly tuned through controlling these factors. For example, when the dealloying temperature in molten Cu increased from 1200 °C to 1400 °C, the ligament width from Ti-40Ta increased from 1.25 ± 0.44 μm to 4.01 ± 1.0 μm; likewise, increasing the dealloying time from 10 s to 60 s, the ligament width increased from 1.17 ± 0.32 μm to 1.25 ± 0.44 μm [214]. Fig. 15d shows the typical porous surface of dealloyed Ti-Ta alloys, where bulk Ti-Ta was specifically dealloyed in CuAg (T_m_: ∼780 °C) bath for 1 h [212]. Analogous to LMD, solid metal dealloying of Ti-Ta was proposed by Wu et al. [216]. They electrochemically deposited a Cu layer on Ti-61.8Ta and then annealed the plated alloy at 800/900 °C for 2-8 h to realise selective extraction. Insufficient dealloying in either liquid or solid metals can constrain the process to the alloy surface while the interior remains intact, creating an open porous surface layer. This largely retains the mechanical properties of the precursor Ti-Ta alloys and simultaneously enhances the bioactive functions. It should be noted that the surface layer of the dealloyed Ti-Ta alloys is predominated by Ta with possibly trace Ti residue inside the ligaments, which presumably offers another advantage to dealloyed Ti-Ta implants besides the porous surface because Ta exhibits a superior biocompatibility to Ti [217]. The biological properties of dealloyed Ti-Ta alloys remain unexplored so far. Nonetheless, the improved cell viability of dealloyed Ti-Zr alloy and upregulated alkaline phosphatase activity of dealloyed Ti-6Al-7Nb alloy are indicative of the positive effect of LMD on Ti-Ta alloy's biological response [218,219].

### 5.5 Alkaline treatment

Like hydrothermal oxidation, alkaline treatment involves immersing Ti-Ta alloy in a pre-heated alkaline solution for hours to induce the alloy surface modification topologically and compositionally. Hulka et al. [145] observed that the surface of bulk Ti-(5, 15, 25)Ta after being treated in 10 mol/L NaOH for 24 h at 60 °C was coated with a smooth film consisting of sodium titanate, sodium tantalate, TiO_2_ and Ta_2_O_5_. The resulting film, however, can be roughened by a subsequent heat treatment in air, as inferred from the study by Gao et al. [76]. The authors further isothermally held the alkaline-treated Ti-15Ta at 600 °C for 1 h, which created shallow nanopores on the surface (Fig. 15e). XRD and EDS analyses indicate the surface contains Na_2_Ti_6_O_13_, TiO_2_ and Ta_2_O_5_. Alkaline-treated Ti-Ta alloys exhibited improved corrosion resistance and outstanding apatite formation ability with or without subsequent heat treatment. After being immersed in SBF for 7 days, the alkaline heat-treated Ti-15Ta was covered with abundant fluffy apatite particles.

### 5.6 Ultrasonic cavitation and particle micro-abrasion

This technique relies only on mechanical force to roughen the Ti-Ta surface. Fu et al. [220,221] introduced a mechanical pathway to produce a rough micro- and nano-featured surface on Ti-Ta cuboid through synergistic ultrasonic cavitation (20 ± 0.5 kHz, 1500 W, 10 min) and micro-abrasion using spherical SiO_2_ particles. Such severe plastic deformation created circular erosion craters on the sample surface (Fig. 15f) [220]. Increasing the SiO_2_ particle size and/or its concentration in the abrasion suspension essentially increased the crater diameter and depth, thus a greater crater volume and surface roughness. For example, SiO_2_ particles of Φ10 μm at 2 wt% concentration offered a mean crater volume of 2625 μm^3^ and surface roughness of 0.311 μm, which increased to 11,173 μm^3^ and 1.201 μm, respectively, when using Φ30 μm particles. The thus roughened surface of Ti-Ta with erosion craters exhibited improved biocompatibility for osteoblast cells, where cell absorbance increased by 10-50% with the SiO_2_ size increasing from 10 μm to 30 μm, or by 60-75% with the SiO_2_ concentration increasing from 2 wt% to 8 wt% [221] (see details in Section 7.3).

## 6 Physicochemical properties of Ti-Ta alloys

### 6.1 Wettability

A wettable surface is highly desired for Ti-Ta implants, which enhances the adsorption of water and protein molecules at the early implant-host bone interaction stage [189,[222], [223], [224]]. The chemistry and topography of the Ti-Ta alloys are decisive to their wettability [225].

**Fig. 16:**
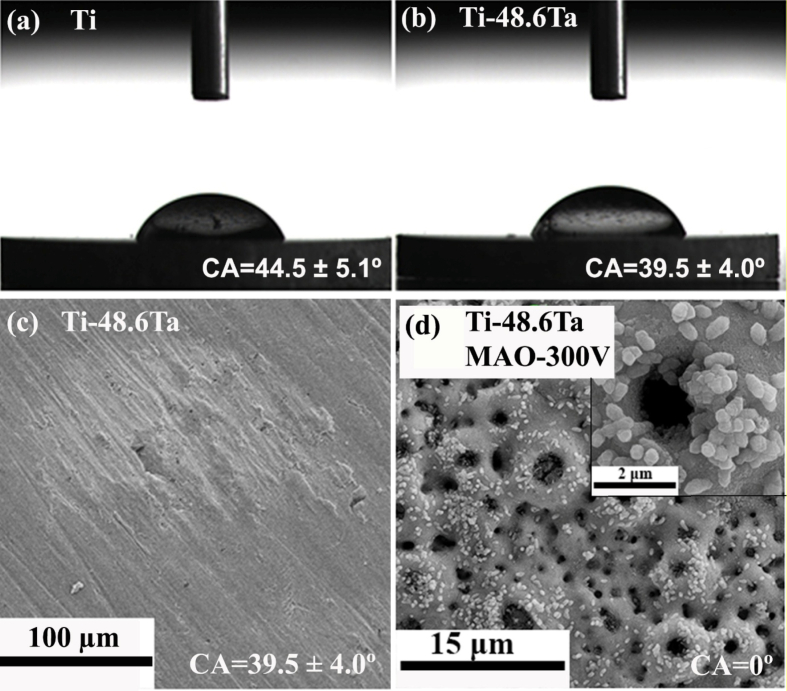
Water contact angle (CA) on Ti-Ta substrates: (a) Ti (44.5 ± 5.1°) and (b) Ti-48.6Ta (39.5 ± 4.0°) showing the effect of alloy chemistry on wettability; (c) surface of the as-ground (by 240 grit abrasive paper) Ti-48.6Ta corresponding to (b) (CA = 39.5 ± 4.0°), and (d) a hierarchical porous Ti-48.6Ta surface after MAO at 300 V with superhydrophilicity (CA = 0°) [196].

Chemically, Ti-Ta alloy appears to be essentially hydrophilic to a wide range of liquids with contact angles of <90° in most cases (macroscopic apparent wettability measured under static sessile drop conditions using a goniometer). For example, within the same study and under identical surface preparation (as-ground 240-grit), Huang et al. [196] reported that Ti-48.6Ta had a lower water contact angle (39.5 ± 4.0°) than Ti (44.5 ± 5.1°), as shown in Fig. 16a and b. Similarly, Wu et al. [151] found that as-polished Ti-25Ta made by melting and heat treatment had a contact angle between 69.3 ± 2.7° and 79.4 ± 2.3° when tested with artificial saliva; Mitra et al. [46] investigated the wettability of as-built Ti-10Ta and Ti-25Ta fabricated by DED-LB/M to various fluids commonly used in biological property assays, and observed their contact angles ranged from 23 ± 2° (Ti-10Ta to ethylene glycol) to 42 ± 2° (Ti-10Ta to Dulbecco's Modified Eagle Medium). Li et al. [105] measured decreasing contact angles of PBF-LB/M Ti-Ta alloys to water (from 55.8° to 52.4°) and dimethyl sulfoxide (DMSO) (from 33.1° to 25.6°) as Ta content increased from 25% to 75%. Such hydrophilic wettability of Ti-Ta alloys is mainly associated with the hydrophilic TiO_2_-Ta_2_O_5_ layer on the surface [86,226]. It can improve protein adsorption and support attachment and proliferation of osteoblast-like tumor cells (see detailed discussion in Sections 7.1, 7.2).

Surface topography and its corresponding roughness are two key factors affecting the wettability of Ti-Ta alloys [227,228]. Within individual studies, different surface preparations have shown substantial differences in apparent contact angle. For example, Ti-93.8Ta coating on Ti substrate consists of sub-micron triangular pyramid-like grains and has a water contact angle of 106 ± 2.5° [195]; whereas Ti-48.6Ta polished by 240 grit abrasive paper has a water contact angle of 39.5 ± 4.0° [196], and Ti-60Ta plate polished with 600 grit sandpaper has a water contact angle of 54.7° [209]. Liu et al. [119] detected a reduced water contact angle from approximately 70° to 30° as the surface roughness of Ti-50Ta lattices increased from approximately 11 μm to 22 μm. The lattices have a pore diameter of about 0.2-1 mm, which may have also influenced the measurement.

In Section 5, we have introduced techniques applied to modify the surface topography of the Ti-Ta alloys. Different surface topographies alter the surface roughness and their corresponding wettability.

• Densely arrayed oxide nanotubes on Ti-Ta surface by anodization (Fig. 15a). Capellato et al. [200] reported the hydrophilic behavior of anodized Ti-30Ta with nanotubes using a 2 mL droplet volume, which has water contact angles between 36.66 ± 1.39° and 41.31 ± 0.96ᴼ. These angles appear to increase with the increase of surface roughness. Annealing the anodized Ti-30 Ta alloy further improved the wettability [229]. Thampi and Ramanathan [195] reported another case, the nanotubes formed on Ti-93.8Ta remarkably improved surface wettability: The water contact angle (a 0.25 μL droplet) dropping from 106 ± 2.5° to 51 ± 1.0°. The nanotube characteristics, Ta content, and droplet size likely contributed to the different contact angles reported, 15.68° [[198], [51]] ± 1.0° [195]. The improved wettability of anodized Ti-Ta is attributed to the liquid penetrating through the nanotubes.
• Firmly adherent and porous oxide coatings on Ti-Ta surface by MAO (Fig. 15b). Huang et al. [196] have observed that surface roughness rises from 0.26 μm (the polished alloy in Fig. 16c) to 0.85 ± 0.03 μm (a hierarchical porous surface after MAO treatment at 300 V in Fig. 16d). At the same time, the water contact angle falls from 39.5 ± 4.0° to almost zero, indicating super-hydrophilicity as a result of surface topology modification. Heat treatment does not further improve the hydrophilicity of the oxidized Ti-Ta alloy [230]. This highlights that surface modification is a more decisive factor for wettability than the alloy microstructure, as stated in Ref. [231].
• Oxide nanoparticles on the alloy surface by hydrothermal-precalcification treatment. The formation of cauliflower-like morphology TiO_2_ particles of 20-40 nm on the as-polished Ti-60Ta plate surface (Fig. 15c) reduced the contact angle of distilled water from 54.7° to nearly zero [209]. Such a transition to superhydrophilicity suggests that the nano-topography enables the fluid to be fully absorbed into the nano-surface features. This superhydrophilicity facilitates the formation of a favorable hydration layer that influences protein adsorption and subsequent cell adhesion (will be discussed in Section 7.3) [232].

### 6.2 Corrosion resistance in simulated body fluid

Ti-Ta alloys exhibit outstanding corrosion resistance in acidic or alkaline solutions, and in most cases, they are superior to pure Ti. The widely accepted underlying reason is the formation of protective and self-adherent TiO_2_-Ta_2_O_5_ on the alloy surface. Both oxides are immune to most neutral media and acids except for hydrogen fluoride (HF), whilst Ta_2_O_5_ appears more resistant than TiO_2_. Therefore, increasing Ta content leads to a higher fraction of Ta_2_O_5_ in the film and thereby a better corrosion resistance [233], corroborated by their electrochemical examinations in 20-80 wt% H_2_SO_4_ [54,143], 6 M HNO_3_ [234], 5% HCl [165,193], 0.9% NaCl [41,195,235], etc.

#### 6.2.1 In neutral simulated body fluid

**Fig. 17:**
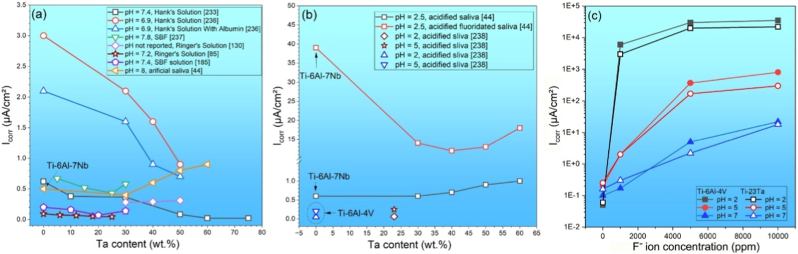
Corrosion response of Ti-Ta alloys to various media. Corrosion current density (I_corr_) as a function of Ta content: (a) in neutral media with pH around 7 such as SBF, Hank's solution, Ringer's solution, and protein-containing solutions, and (b) in acidic media with pH between 2 and 5. (c) I_corr_ of Ti-23Ta in fluoridated artificial saliva in comparison with Ti-6Al-4V [238].

In such fluids, most studies report that Ti-Ta alloys generally display a corrosion behavior comparable to or better than pure Ti, Ti-6Al-4V and Ti-6Al-7Nb [44,85,97,130,133,149,195,235]. Gordin et al. [130] investigated the corrosion performance of Ti-(30, 40, 50)Ta fabricated by cold crucible levitation melting in Ringer's solution (Na^+^ = 147.0 mmol/L; K^+^ = 4.0 mmol/L, Ca^2+^ = 2.3 mmol/L, Cl^−^ = 155.5 mmol/L). Like the reference alloy Ti-6Al-4V, these Ti-Ta alloys consisted of stable α and β phases. Electrochemical tests showed that Ti-Ta alloys corroded similarly in the solution. However, they were more resistant than Ti-6Al-4V, particularly in terms of the passive current density, of which Ti-Ta alloys (12-14 mA/cm^2^) was about one twentieth that of Ti-6Al-4V (243 mA/cm^2^). In another study, Kim et al. [149] examined the corrosion behavior of metastable Ti-(10-40)Ta alloys fabricated by arc melting and observed that they were comparable to pure Ti and Ti-6Al-4V in Hank's solution, but better than Ti-Nb alloys in either Hank's solution or 0.1% lactic acid. AM Ti-Ta alloys also display good corrosion resistance to Ringer's solution, reported by Zhao et al. [85]. In their study, Ti-Ta alloys containing 0, 6, 12, 18 and 25%Ta were prepared by PBF-LB/M, with a relative density between 95% and 99%. Their corrosion resistance gradually improved with increasing Ta content, for instance, the corrosion rate decreased monotonically from 3.69 × 10^−4^ mm/year in pure Ti to 1.72 × 10^−4^ mm/year in Ti-25Ta. On the contrary, some studies observed inferior corrosion resistance of Ti-Ta alloys to pure Ti or Ti-6Al-4V [69,130,235]. Huang et al. [69] found that as-sintered Ti-Ta was slightly less resistant to phosphate-buffered solution (pH = 7.4) than pure Ti. In their study, Ti-48.6Ta alloy consisted of alternate Ti-/Ta-rich micro-bands and Ti-/Ta-rich nano-sized α+β lamellae, and this inhomogeneous two-phase lamellar structure might account for its inferior corrosion resistance. With the increase of Ta content, most studies report improved corrosion resistance with Ti-Ta alloys in neutral media with few opposite cases, as shown in Fig. 17a [85,130,185,233,236,237]. For example, Mareci et al. [44] found that I_corr_ of Ti-Ta alloys increased monotonically from 0.4 ± 0.1 μA/cm^2^ to 0.9 ± 0.1 μA/cm^2^ in artificial saliva as Ta content increased from 30% to 60%.

**Fig. 18:**
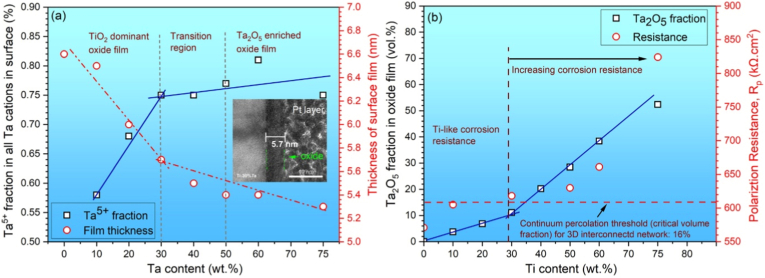
Analyses of naturally passivated oxide film on Ti-Ta alloys and the corrosion behavior of the alloys in an aerated Hank's balanced salt solution [233]. (a) Thickness of the film measured by TEM, and Ta_2_O_5_ fraction in the oxide film measured by X-ray photoelectron spectroscopy, (b) nominal TaO_x_ (including TaO, TaO_4_, and Ta_2_O_5_) volume fraction in the oxide film, and the polarization resistance of Ti-Ta alloys detected by electrochemical impedance spectroscopy.

Increasing Ta content in Ti-Ta alloys leads to variations of the oxide surface film including its thickness and oxide fraction, altering the corrosion behavior. Mendis et al. [233] conducted a comprehensive investigation of the oxide film of as-polished Ti-Ta alloy ingots that were naturally passivated in ambient atmosphere for 48 h, Fig. 18. As Ta content increased from 10% to 75%, TaO_x_ (including Ta_2_O_5_, TaO_2_ and TaO) in the oxide layer increased from 3.71 vol% to 52.36 vol%, and particularly Ta_2_O_5_ alone increased from 2.16 vol% to 39.27 vol%. Correspondingly, the polarization resistance of Ti-Ta alloys in an aerated Hank's balanced salt solution increased, although the thickness of the oxide film reduced with Ta content. The authors pointed out that ∼30%Ta appeared to be a threshold composition, above which a continuous Ta_2_O_5_ skeletal network would be formed in the oxide layer. Alkaline heat treatment – immersing as-polished Ti-Ta fabricated by arc melting in NaOH solution and then treating at elevated temperatures in air, can enhance the presence of TiO_2_/Ta_2_O_5_ surface layer and thus improve the alloy corrosion resistance; re-immersing the treated alloy in SBF for several days can further increase the corrosion resistance because of the formation of a hydroxyapatite layer extra to existing TiO_2_/Ta_2_O_5_ layer [76].

#### 6.2.2 In acidic/fluorine-containing environments

Ti-Ta alloys for dental applications are susceptible to acid or fluoride (F^−^) ion attack. Therefore, their resistance to such corrosive environments is of significance. For most prophylactic products for dental treatment, their concentration of F^−^ ions varies, around 200 ppm in buccal rinses, 1000-1500 ppm in toothpastes, and 10000-20000 ppm in gels [238]. Mareci et al. [44] systematically studied the corrosion behavior of Ti-(30, 40, 50, 60)Ta alloys in four artificial saliva electrolytes. Aerated artificial saliva was used as the reference electrolyte, and then lactic acid and fluoride were added separately or together. Experimental results showed all Ti-Ta alloys examined and reference Ti-6Al-7Nb produced a passive and stable oxide surface film in artificial saliva (pH = 8), acidified saliva (pH = 2.5), and fluoridated saliva (pH = 8, 1000 ppm F^−^). With the increase of Ta content, all compositions remained passive in acidified saliva, but their resistance growingly deteriorated, as inferred from Fig. 17b. When introduced fluorine together (pH = 2.5, 1000 ppm F^−^), all the alloys were actively corroded and appeared to be worse with increasing Ta content (Fig. 17b). It is found that the electrolyte intensely attacks α phase in the structure, i.e., the equiaxed primary and acicular α in Ti-6Al-7Nb, intergranular and lamellar α in Ti-30Ta, and needle-like α in Ti-50Ta. Due to the differences in the morphology and volume fraction of the α phase, Ti-6Al-7Nb, Ti-30Ta and Ti-50Ta exhibited overall, localised, and uniform corrosion behavior. Ti-60Ta containing the least α phase, however, did not exhibit the best corrosion resistance; on the contrary, its I_corr_ reached 18.0 ± 2.0, higher than other compositions (12.0-14.0 on average) but still lower than that of Ti-6Al-7Nb (39.0 ± 4.0).

Ti-Ta alloys are immune to fluorine in neutral solution but vulnerable to fluoridated acidified solution. This was further corroborated in other studies. Robin and Meirelis [238] investigated the corrosion response of Ti-23Ta in artificial saliva and compared with that of Ti-6Al-4V. The saliva was an aqueous solution containing NaCl, KCl, CaCl_2_, NaH_2_PO_4_, and Na_2_S, and added with NaF to offer an F^−^ ion concentration of 0, 1,000, 5000 and 10,000 ppm or added with HCl/NaOH to offer pH values of 2, 5 and 7. Corrosion assays at 37 ± 1 ᴼC indicated that both alloys were passive only in solutions at pH = 2 without F^−^ ion, pH = 5 in F^−^ ion ≤1000 ppm, or pH = 7 with in all F^−^ ion concentrations; increasing F^−^ ion concentration or decreasing pH value led to reduced corrosion resistance and less stable passive oxide film, as evidenced in Fig. 17c. Compared to Ti-6Al-4V, Ti-23Ta alloy exhibited slightly better corrosion resistance to all the saliva examined. Fojt et al. [239] carried out similar investigations of Ti-15Ta and Ti-30Ta in comparison to pure Ti and Ta in NaCl aqueous solution. The solution was adjusted to pH 4.2/200 ppm F^−^, pH 5.8/1000 ppm F^−^ and pH 7/0 ppm F^−^ by adding phthalate buffer solution and NaF. They observed that as the solution became more aggressive (lower pH) Ti-Ta alloys were less resistant, and more Ti/Ta ions released; a higher Ta content enhanced the corrosion resistance and reduced the ion release in an acidic solution, for example, 18.9 ± 1.4 μg/cm•h (Ti) plus 2.49 ± 0.32 μg/cm•h (Ta) in Ti-15Ta vs. 3.064 ± 0.086 μg/cm•h (Ti) plus 0.923 ± 0.013 μg/cm•h (Ta) in Ti-30Ta, when tested in pH 4.2/200 ppm F^−^. The decreased corrosion resistance of Ti-Ta alloys in fluoridated acidified solution is attributed to the combination of F^−^ with H^+^ leading to the formation of HF which reacts with Ti oxide component in the surface film to form soluble Ti-F compounds, consequently destroying the protective surface film [44]. While Ti oxide is attacked, Ta oxide component in the film remains relatively stable. This selective degradation in a fluoridated acidified environment can increase local Ti-ion release [240,241] and induce adverse reactions [217,242].

## 7 Biological responses of Ti-Ta alloys

### 7.1 *In vitro* evaluation

The static biological response of Ti-Ta alloys to cells has been intensively assessed and compared to other biomedical materials that are commonly used in clinical surgeries. In a typical *in vitro* protocol, cells are seeded on the surface of Ti-Ta alloys and control materials (such as pure Ti, Ta or Ti-6Al-4V), and incubated in a culture medium (commonly Dulbecco's Modified Eagle Medium (DMEM) supplemented with 10% Fetal Bovine Serum) under standard conditions (5-10%CO_2_, 37 °C and 95-100% humidity). After culturing for a particular time (from hours to weeks), samples are stained, fixed and/or dehydrated for cell adhesion or morphology analyses on a fluorescent microscope. In such analyses, cell morphology offers important insight into the quality of cell–material interactions. Well-spread cells with flattened bodies, extended filopodia, and defined focal adhesion points typically indicate strong adhesion, high cytoskeletal organization, and favorable surface compatibility. Conversely, rounded or poorly spread cells may signal weak interaction or early detachment [46,86,189,243]. At the same time, they can be evaluated using a colorimetric assay such as 3-(4,5-dimethylthiazol-2-yl)-2,5-diphenyl tetrazolium bromide (MTT), 3-(4,5-dimethylthiazol-2-yl)-5-(3-carboxymethoxyphenyl)-2-(4-sulfophenyl)-2H-tetrazolium (MTS), water-soluble tetrazolium salt-1 (WST-1), and water-soluble tetrazolium salt-8 (WST-8) to determine cell viability and proliferation. These test results and image analysis present a comprehensive expression of cell metabolism, attachment, viability, proliferation, differentiation, and mineralization behavior over culture time.

Ti-Ta alloys' *in vitro* biological responses e.g., cell adhesion, viability, proliferation and osteogenesis, are crucial indicators of their suitability for implant applications. These responses are influenced by the alloy's composition, structure, porosity, and surface features. Table 8, Table 9 summarise these trends. We discuss them in detail as follows.

**Table 8:** *In vitro* biocompatibility of dense Ti-Ta alloys compared to the control.

Alloys (wt.%)	Fabrication technique	Surface characteristics^a^	Cell line	Cell proliferation		ALP		ECM mineralization		Ref.
				Original value	%^p^	Original value	%^p^	Original value	%^p^	
Ti-5Ta	-	As-polished	Human squamous epithelial cells (SKMES)	2.172 ± 0.07^c^	99	-				[43]
			Mouse embryonic fibroblasts (NIH-3T3)	1.595 ± 0.13	111					
			Mouse fibroblasts (L929)	1.101 ± 0.08	107					
Ti∗	-		SKMES	2.195 ± 0.10	100	-				
			NIH-3T3	1.436 ± 0.09	100					
			L929	1.028 ± 0.10	100					
Ti-10Ta	Melting + ST + WQ	As-polished	L-929 (non-filtrated)	87^d^	87	-				[193]
			L-929 (filtrated)	96	96					
Ti-30Ta			L-929 (non-filtrated)	91	91	-				
			L-929 (filtrated)	100	100					
Ti-30Ta	Melting + STA + WQ		L-929 (non-filtrated)	91	91	-				
			L-929 (filtrated)	99	99					
Ti-70Ta	Melting + ST + WQ		L-929 (non-filtrated)	93	93	-				
			L-929 (filtrated)	102	102					
Ti			L-929 (non-filtrated)	89	89	-				
			L-929 (filtrated)	95	95					
Plastic well (positive)∗	-	-	L-929 (non-filtrated)	100	100	-				
			L-929 (filtrated)							
Ti-25Ta	Melting + HT at 700 °C	As-polished CA = 75.2 ± 1.9**°**^b^	Human osteosarcoma cells (MG-63)	98^e^	98	-				[151]
			NIH-3T3	110	110					
	Melting + HT at 800 °C	As-polished CA = 79.4 ± 2.3**°**	MG-63	99	99	-				
			NIH-3T3	104	104					
	Melting + HT at 900 °C	As-polished CA = 73.4 ± 2.9**°**	MG-63	97	97	-				
			NIH-3T3	111	111					
	Melting + HT at 1000 °C	As-polished CA = 69.3 ± 2.7**°**	MG-63	103	103	-				
			NIH-3T3	101	101					
Plastic well (positive)∗	-	-	MG-63	100	100	-				
			NIH-3T3	100	100					
Ti∗	PBF-LB/M	As-polished, CA = 57.5°, 2 μL droplet	Mouse embryonic osteoblast precursor cells (MC3T3-E1)	1.45^f^	100	11.00^q^	100	7.00^u^	100	[105]
Ti-25Ta		As-polished, CA = 55.8°, 2 μL droplet		1.52	105	15.10	137	12.50	179	
Ti-55Ta		As-polished, CA = 53.0°, 2 μL droplet		1.60	110	13.76	125	21.25	304	
Ti-75Ta		As-polished, CA = 52.4°, 2 μL droplet		1.56	108	15.50	141	18.75	268	
Ta		As-polished, CA = 49.1°, 2 μL droplet		1.56	108	17.50	159	14.50	207	
Ti∗		As-polished, CA = 57.5°, 2 μL droplet	Raw264.7 cell lines	1.70	100	11.24	100	-		
Ti-25Ta		As-polished, CA = 55.8°, 2 μL droplet		1.75	103	15.50	138	-		
Ti-55Ta		As-polished, CA = 53.0°, 2 μL drople		1.84	108	17.50	156	-		
Ti-75Ta		As-polished, CA = 52.4°, 2 μL drop		1.75	103	13.76	122	-		
Ta		As-polished, CA = 49.1°, 2 μL droplet		1.80	106	13.74	122	-		
Ti-40Ta	Ingot metallurgy	As-polished	NIH-3T3	78300^g^	111	-				[45]
		As-ground (SiC 600#)		40625	108					
Ti-50Ta		As-polished		76562	109	-				
		As-ground (SiC 600#)		31250	83					
Ti-6Al-4V		As-polished		71875	102	-				
		As-ground (SiC 600#)		40625	108					
Ti∗		As-polished		70312	100	-				
		As-ground (SiC 600#)		37500	100					
Ti-48.6Ta	PM	As-polished	Murine osteoblast cells (MC3T3-E1)	100^h^	100	-				[60]
			Murine bone marrow stroma cells (OP9)	87	87					
Ti-55.8Ta			MC3T3-E1	105	105	-				
			OP9	85	85					
Ti-61.8Ta			MC3T3-E1	96	96	-				
			OP9	85	85					
Ti-67.1Ta			MC3T3-E1	92	92	-				
			OP9	83	83					
Ti-79.1Ta			MC3T3-E1	98	98	-				
			OP9	83	83					
Plastic well (negative)∗	-		MC3T3-E1	100	100	-				
			OP9	100	100					
Ti-48.6Ta	PM	As-ground (SiC 240#) CA = 39.5 ± 4.0**°**	Human osteosarcoma cells (SAOS-2)	1.4^i^	108	1.6^r^	114	0.27^v^	108	[196]
Ti		As-ground (SiC 240#) CA = 44 ± 5.1**°**		1.3	100	1.4	100	0.25	100	
Ti-48.6Ta	SPS	As-sintered	hBMSCs	1.8^j^	106	-				[69]
Ti∗				1.7	100	-				
Ti-25Ta	PBF-EB/M Scan speed (0.65 m/s)	As-polished CA = 71**°,** 4 μL droplet	MC3T3-E1	104^k^	104	-				[96]
Ti-25Ta	PBF-EB/M Scan speed (0.675 m/s)	As-polished CA = 64**°,** 4 μL droplet		105	105	-				
Ti-25Ta	PBF-EB/M Scan speed (0.7 m/s)	As-polished CA = 60**°,** 4 μL drople		92	92	-				
Plastic well (negative)∗	-	-		100	100	-				
Ti-25Ta	PBF-LB/M	As-built	Bone marrow mesenchymal stem cells (BMSCs)	1.2^i^	150	0.055^s^	167	1.2^w^	171	[86]
Ti∗				0.8	100	0.033	100	0.7	100	
Ti-25Ta	PBF-LB/M	As-polished	Human bone marrow mesenchymal stem cells (hBMSCs)	7^l^	88	1^t^	263	1.6^x^	800	[244]
Ti-65Ta				9	113	1.13	297	1.65	825	
Ti-6Al-4V∗				8	100	0.38	100	0.2	100	
Ti-30Ta	PBF-LB/M	As-polished	MC3T3-E1 preosteoclast cells	1.03^m^	103	-				[90]
				1.06	106	-				
				1.07	107	-				
Plastic well (negative)∗				1	100	-				
Ti-5Ta	Cold spray	As-polished	Mouse Fibroblast (GPE86)	28^n^	140	-				[107]
Ti-10Ta				35	175	-				
Ti-30Ta				55	275	-				
Ti∗			GPE86	20	100	-				
Ti-30Ta	Cold spray	As-polished	GPE86	70^n^	233	-				[246]
Ti∗	-			30	100	-				
Ti-30Ta	Cold spray	As-sprayed	GPE86	82^o^	75	-				[245]
Ta				89	81	-				
Ti-6Al-4V	PBF-EB/M	As-built		68	62	-				
Plastic well (positive)∗	-	-		110	100	-				

∗Control material.

a Unless otherwise specified, reagent water was used under normal conditions (goniometer, macroscopic sessile-drop conditions, room temperature) for contact angle values (CA) measurements.

b Artificial saliva (pH = 7.0) as the test liquid under normal conditions (no droplet size reported).

c Optical density (OD)/absorbance measured at 550 nm, day 1, using MTT assay.

d Relative cell proliferation values measured at day 14, using the MTT assay.

e 540 nm, day 5, using the MTT assay.

f 450 nm, day 7, using CCK-8 assay.

g Number of cells after 48 h using MTT Assay at 570 nm.

h 570 nm, day 4, using MTT assay, normalised to the negative control (DMEM, 100%).

i 450 nm, day 5, using the CCK-8 assay.

j 450 nm, day 7, using the CCK-8 assay.

k Cells proliferation normalised to the negative control, OD of 450 nm, day 7 using CCk-8 assays.

l Cell proliferation values of 7 days using the MTS assay absorbance, normalised to the initial 4 h absorbance.

m day 2 using the MTT assay normalised to the negative control (DMEM, 100%).

n Absolute cell fraction (%): Percentage of the specimen surface area covered by adhered cells after 6 h, quantified from SEM images.

o day 1, using MTS assay, normalised to TCPS (Tissue Culture Polystyrene Medium) Control.

p Values normalised to their respective control and expressed as a percentage of the control.

q Quantitative Mean ALP Fluorescent Intensity.

r Quantitative analysis of ALP activity measured at day 5.

s Quantitative analysis of ALP activity measured at day 14 in u/mg protein.

t Picogreen, pNP Assay (nmol, min, ugDNA) values after 7 days, normalised to Same-Well DNA (Picogreen, dsDNA Assay/Invitrogen).

u Percentage of stained area using Alizarin red S Assay.

v 570 nm, day 10, Alizarin red S assay.

w 620 nm, day 21, Alizarin red S assay.

x Osteoimage Intensity after 4 weeks evaluated using Lonza Stain.

**Table 9:** *In vitro* biocompatibility of porous Ti-Ta alloys compared to their control.

Alloys (wt.%)	Fabrication technique	Porous structure	Surface characteristics^a^	Cell line	Cell proliferation		ALP		ECM mineralization		Ref.
					Original value	%^i^	Original value	%^i^	Original value	%^i^	
Porous Ti-Ta alloys											
Ti-30Ta	PM	60% porosity, 0.1-90 μm nonuniform pores	As-processed	Normal human osteoblast (CC-2538)	109^c^	**-**					[73]
				Human periodontal ligament fibroblast (CC-7049)	110						
		72% porosity, 0.1-90 μm nonuniform pores		CC-2538	98						
				CC-7049	125						
		76% porosity, 0.1-90 μm nonuniform pores		CC-2538	106						
				CC-7049	112						
Dense Ti∗	-			CC-7049	100	**-**					
Ti-20Ta	DED-LB/M	Grid, 400–450 μm pore size	As-built	Mouse calvarial preosteoblasts (ATCC CRL-2593)	0.008^d^	24	-				[121]
Ti-40Ta					0.01	29	-				
Ti-60Ta					0.02	59	-				
Ti-80Ta		Grid, 400 μm pore size			0.031	91	-				
Ta∗		Grid, 500 μm pore size			0.034	100	-				
Ti-10Ta	DED-LB/M	200-300 μm nonuniform pores	As-built, CA = 42 ± 2°^b^	Human fetal osteoblasts (hFOBs)	0.252^e^	168	-				[46]
Ti-25Ta			As-built, CA = 38 ± 1°		0.248	165	-				
Dense Ti∗		-	TiO_2_ nanotubes		0.15	100	-				
Ti-25Ta	PBF-LB/M	Trabecular, 478 μm pore size	-	Mouse embryonic pre-osteoblasts (MC3T3-E1)	2.6^f^	200	-				[223]
Ti∗		Trabecular, 486 μm pore size	-		2.0	154	-				
Ti-25Ta	PBF-LB/M	Gyroid, 60% porosity, 429 ± 15 μm pore size	As-ground (SiC 4000#)	Alveolar-bone-derived human teeth osteoblast cells	74^g^	70	-				[113]
		Diamond, 60% porosity, 276 ± 17 μm pore size			86	81					
		Schwarz p., 60% porosity, 700 ± 16 μm pore size			122	115					
Dense Ti-25Ta∗		-			106	100	-				
Ti-50Ta	PBF-LB/M 100W	Voronoi tessellation trabecular, 59% porosity	As-built	MC3T3-E1	0.2^f^	25	1.2^j^	100	0.18^m^	72	[119]
	PBF-LB/M 220W	Voronoi tessellation trabecular, 39% porosity	As-built		1.2	150	1.7	142	0.3	120	
	PBF-LB/M 340W	Voronoi tessellation trabecular, 30% porosity	As-built		1.2	150	1.6	133	0.28	112	
Ti∗	PBF-LB/M	Voronoi tessellation trabecular, 42% porosity	As-built		0.8	100	1.2	100	0.25	100	
Ti-25Ta	PBF-LB/M	BCC scaffold, 60% porosity, 321 μm pore size	As-built	MC3T3-E1	2.67^f^	105	0.89^k^	110	0.57^m^	107	[176]
Ti-50Ta		BCC scaffold, 60% porosity, 340 μm pore size			2.81	111	0.98	121	0.91	172	
Ti-75Ta		BCC scaffold, 60% porosity, 331 μm pore size			2.88	113	1.02	126	1.02	192	
Ti∗		BCC scaffold, 60% porosity, 302 μm pore size			2.54	100	0.81	100	0.53	100	
Porous Ti-Ta alloys with surface modification											
Ti-48.6Ta	PM + MAO at 250 V	-	Microporous, 0.15-15 μm pore size, CA = 0**°**	SAOS-2	1.5^h^	115	1.7^l^	121	0.49^n^	126	[196]
	PM + MAO at 300 V	-	Hierarchical micro-nano-porous, 0.05-15 μm pore size, CA = 0**°**		1.6	123	1.8	129	0.52	133	
Dense Ti∗	PM	-	As-ground (SiC 240#), CA = 44 ± 5.1**°**		1.3	100	1.4	100	0.39	100	

∗Control material.

a Unless otherwise specified, reagent water was used under normal conditions (goniometer, macroscopic sessile-drop conditions, room temperature) for contact angle values (CA) measurements.

b Dulbecco's Modified Eagle Medium (DMEM) as the test liquid under normal conditions (0.5-1.0 μL droplet).

c MTT assay absorbance of 4 days, normalised to the Ti-conditioned medium.

d Cell growth after 12 days, the increase in sample weight in grams.

e OD absorbance measured at day 7, using MTT assay.

f 450 nm, day 7, using the CCK-8 assay.

g Nuclei count at 3 days, proportional to cell number and proliferation, as measured by the PicoGreen DNA assay.

h 450 nm, day 5, using the CCK-8 assay.

i Values normalised to their respective control and expressed as a percentage of the control.

j 405 nm, day 7, using ALP assay.

k day 14, in u/mg protein, using ALP assay.

l Quantitative analysis of ALP activity measured at day 5.

m 562 nm, day 21, using Alizarin red S assay.

n 570 nm, day 14, using Alizarin red S assay.

#### 7.1.1 Dense Ti-Ta alloys

Several significant observations can be summarized from the extensive *in vitro* study of dense Ti-Ta alloys, as follows:

##### 7.1.1.1 Adhesion, viability and proliferation of cells

Ti-Ta alloys are proved to be compatible with a wide range of cells, e.g., epithelial, fibroblasts, osteoblast-like cells and stem cells [19,60]. All of these cells can adhere well to and proliferate on the Ti-Ta alloy surfaces regardless of their surface finish, delivering cell viability comparable to or better than pure Ti, pure Ta, and Ti-6Al-4V (>100% cell proliferation in Table 8).

**Fig. 19:**
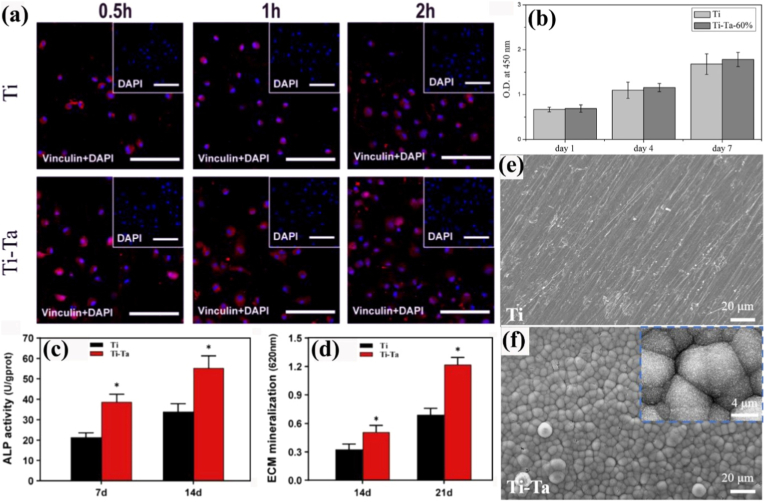
(a) CLSM images showing the adhesion of BMSCs on the surface of pure Ti and Ti-25Ta after 0.5 h, 1 h and 2 h culture in DMEM [86]. As-built disc samples of 10 × 10 × 2 mm^3^ in size were printed by PBF-LB/M. Samples were stained by mouse anti-vinculin monoclonal antibody, Rhodamine-conjugated secondary antibody (red) and subsequently by DAPI to express cell nuclei (blue). Scale bar: 200 μm. (b) Optical density of human BMSCs seeded on fully dense as-sintered Ti-48.6Ta surface and pure Ti after 1-, 4-, and 7-day culture [69]. Ti-Ta alloy was prepared by hot pressing, hot rolling, cold rolling, annealing and water quenching, forming a lamellar structure. Cells after seeding for 24 h were fixed with 4% paraformaldehyde and stained with 5 μg/mL phalloidin-TRITC. The images show that the cells formed a fusiform shape and were well attached to both sample surfaces. (c) Cell differentiation ability assayed by ALP, and (d) cell mineralization assayed by ARS in as-built Ti-25Ta and pure Ti [86]. Bulk samples were made by PBF-LB/M and seeded with BMSCs for up to 21 days. Surface morphology of pure Ti (e), and Ti-25Ta (f) after immersion in simulated body fluid for 4 weeks [86]. The spheroid-like particles on Ti-25Ta consisted of TiO_2_, Ta_2_O_5_ and hydroxyapatite containing A-type carbonate.

Compared to their controls, favorable cell adhesion has been consistently observed on Ti-Ta alloys across as-built and as-polished surfaces. For example, Zhao et al. [86] cultured bone marrow mesenchymal stem cells (BMSCs) on as-built Ti-25Ta from PBF-LB/M for 0.5 h, 1 h and 2 h, and observed higher cell spreading and attachment at all time points compared to pure Ti (Fig. 19a). In another study, Brodie et al. [244] observed comparable human BMSC cell attachment and spreading morphology on polished Ti-6Al-4V, Ti-25Ta and Ti-65Ta made by PBF-LB/M after 4 h of incubation. Cells were elongated with well-organized actin cytoskeletons and numerous filopodia [244]. Zeng et al. [107,245,246] also reported enhanced early cell adhesion on polished Ti-(5-30)Ta cold-sprayed on Ti-6Al-4V. They found that mouse fibroblasts (3 and 6 h culturing in DMEM) exhibited significantly higher early cell fractions (140%, 175% and 275% as listed in Table 8), indicating the cells adhered well on the Ti-Ta surfaces, compared to pure Ti cold-sprayed on Ti-6Al-4V. These results consistently indicate that Ti-Ta provides a surface that supports robust bioactive cell attachment. One contributing factor is the surface oxide layer rich in Ta_2_O_5_, which renders the surface more hydrophilic (lower contact angle) and thus more supportive of cell adhesion.

Cell viability tests focus on short timeframes, e.g., 0.5 h to 4 days. As documented in Table 8, a study by Villa et al. [45] indicated that as-ground and as-polished Ti-(40, 50)Ta supported favorable cell viability (mouse fibroblast cells) by 8-11% higher relative to pure Ti after 48 h of incubation. Prigent et al. [43] also reported the same trends. They investigated the viability of epithelial cells and two types of fibroblast cells on the polished surface of Ti-5Ta, pure Ti and pure Cu. After 48 h of culture in RPMI 1640 media, cells of all types spread out on specimen surfaces except cytotoxic Cu. Quantitatively, Fibroblast cell viability on Ti-5Ta displayed 7-11% higher than pure Ti, while the epithelial cell number was close on Ti-5Ta and pure Ti (Table 8) [43]. Similarly, Liu et al. [90] also investigated cell viability on as-polished Ti-30Ta where 48 h MTT assays using murine pre-osteoblast cell extracts showed no cytotoxicity relative to the negative control (cells cultured in complete medium without exposure to the material). This was consistent with negligible ion release after 3–7 days of immersion, and SEM images further confirmed favorable cell adhesion and spreading. In another study, Liu et al. [60] incubated murine marrow stroma (OP9)/murine osteoblast cells (MC3T3-E1) on as-polished Ti-(48.6-79.1)Ta alloys for 4 days, obtaining viability values ranging from 83% (Ti-67Ta with OP9 cells) to 105% (Ti-55.7Ta with MC3T3-E1 cells) compared to cells grown on tissue culture plastic (the negative control). This proves that Ti-Ta alloys were not cytotoxic according to ISO 10993-5 standard, which defines materials with ≥70% cell viability as non-cytotoxic [60], providing a bioactive environment for cells.

Cell proliferation uses the same assays as cell viability and focuses on relatively long timeframes (e.g., 3 to 7 days). A higher cell viability at earlier time points often correlates with, and may indicate, greater cell proliferation at later stages. This review uses viability values from later time points to represent relative proliferation. Quantitatively, Ti-Ta alloys maintain cell proliferation values ranging from 83% to 138% relative to their controls (Table 8). Most notably, Zhao et al. [86] demonstrated the significant cell proliferation enhancement over 7 days in DMEM, showing that as-built Ti-25Ta PBF-LB/M achieves 138% proliferation rate compared to 100% for the control. Furthermore, Fig. 19b illustrates the cell proliferation of human BMSCs seeded on as-sintered Ti-48.6Ta and pure Ti surfaces for up to 7 days in DMEM [69], revealing better biocompatibility with Ti-Ta than pure Ti. These findings confirm that Ti-Ta alloys support excellent cell adhesion, viability, and proliferation responses.

##### 7.1.1.2 Osteogenesis

Osteogenesis is the process by which bone tissue is created and renewed. It proceeds through osteogenic differentiation and bone mineralization, which can be assayed respectively by alkaline phosphatase (ALP) and Alizarin red staining (ARS). ALP assay measures alkaline phosphatase enzyme of cells as they begin to differentiate into bone-forming osteoblasts. On the other hand, the ARS assay measures the calcium deposition in the bone extracellular matrix (ECM), an important indicator of matrix production and maturation as the differentiation advances. Ti-Ta alloys exhibit an advantageous osteogenesis ability over pure Ti and Ti-6Al-4V [86,244]. Figs. 19c and d are histograms depicting the better ALP activity and ECM mineralization ability of BSMCs cultured on as-built Ti-25Ta than on pure Ti from PBF-LB/M after 7, 14 and 21 days [86]. The corresponding quantitative data in Table 8 show that Ti-25Ta boosted ALP to 167% and mineralization to 171% relative to the Ti control on day 21. Li et al. [105] measured that Ti-(25, 55, 75)Ta alloy's mineralization reached 179%, 304% and 268% that of pure Ti, comparable to and even better than pure Ta (207% that of Ti), at day 7. Brodie et al. [244] demonstrated a similar advantage of as-polished Ti-(25, 65)Ta fabricated by PBF-LB/M in ALP and ECM mineralization ability over Ti-6Al-4V after 4 weeks of culture (297% in ALP and 825% in ECM mineralization as shown in Table 8).

##### 7.1.1.3 Apatite-forming ability

Apatite is a calcium phosphate mineral with the formula Ca_10_(PO_4_)_6_(OH)_2_, naturally found in human bones, which contributes to their hardness and strength [86]. Implant surfaces that form apatite layers in SBF (a solution closely matching human blood plasma) promote osteoclastic resorption, bone regeneration, and remodeling during bone healing. Ti-Ta alloys offer an outstanding apatite-forming ability on their surface due to the spontaneous generation of self-passivating Ta_2_O_5_ and TiO_2_ oxide films. Upon immersion in physiological media, the Ta_2_O_5_ film hydrates to form surface Ta-OH groups that produce a negative charge, nucleating calcium phosphate deposition [86,196]. This apatite-inducing behavior confirms the bioactive nature of Ti-Ta alloys, a predictor of bone-bonding potential. For example, immersing Ti-25Ta in a modified SBF for 4 weeks produced spheroid-like particles clustering densely on the specimen surface as the particles contained a uniform distribution of Ca, P and O elements (apatite substance). This contrasts with the pure Ti surface, which showed no particle formation (Fig. 19e and f) [86].

#### 7.1.2 Porous Ti-Ta alloys

**Fig. 20:**
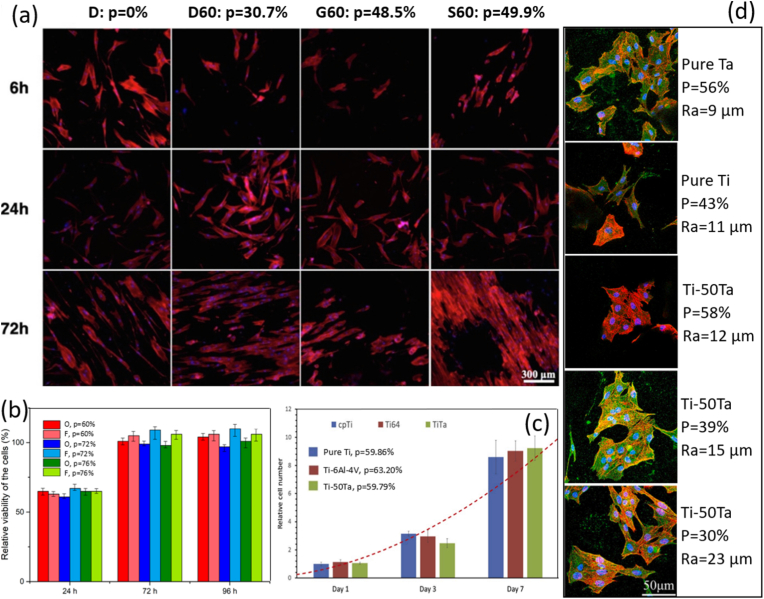
*In vitro* assessment of porous Ti-Ta alloys. (a) CLSM images showing osteoblast cells spreading on dense and Ti-25Ta lattices with a designed porosity of 60% [113]. Dense (D) and lattice specimens with TPMS-gyroid (G60), diamond (D60) and Schwarz (S60) unit cells were fabricated by PBF-LB/M. (b) Cell viability on porous Ti-30Ta with various porosities relative to pure bulk Ti (represented 100% cell viability) [73]. Specimens were fabricated by vacuum sintering of Ti-Ta-Mg powder compacts, in which the evaporation of Mg (30-50 wt%) generated porous scaffolds of 60-76% porosity. Human osteoblast cells (O) and periodontal ligament fibroblast cells (F) were seeded and assayed via MTT. (c) Cell viability on pure Ti, Ti-50Ta and Ti-6Al-4V lattices [117]. All specimens, by PBF-LB/M, were in a cubic cell structure with a porosity of around 60%, and the relative cell number was measured using a dsDNA picogreen assay on a spectrofluorometer. (d) Immunofluorescence staining images of Col-1 expression in Ta, Ti and Ti-50Ta Voronoi tessellation-based lattices at day 5, merged with F-Actin, Col-1 and DAPI images [119]. All lattices were printed by PBF-LB/M at various laser energy densities, leading to variations in porosity (p) and surface roughness (Ra).

The biological response of porous Ti-Ta alloys, including their lattice structures, has been addressed, and we summarized their *in vitro* biological responses in Table 9 [46,73,121,196]. Compared to dense counterparts, a porous structure generally promotes a better cell proliferation rate over time. For example, Soro et al. [113] observed fewer osteoblast cells on porous Ti-Ta surface at the early incubation stage (6 h). However, they found that the cells proliferated faster over time. The cell proliferation rate increased significantly after 72 h of culture, as shown in Fig. 20a. This was further corroborated by a quantitative examination of cell metabolic activity and DNA content using a spectrofluorometer. The initially lower fluorescence signal detected on porous surfaces may largely arise from a measurement artifact rather than an intrinsic material limitation. This occurs because imaging captures only the outer surface; the inner pores and cells colonizing are not fully detected. Studies have also noted that porous 3D scaffolds can hinder the diffusion of assay colorimetric agents within their structure, which can contribute to underestimated results [247]. Once the cells become fully confluent and cover the whole surface (e.g., at day 7), a comparable or even higher cell number can be achieved from porous Ti-Ta. This reflects the enhanced osteoconductivity of porous structures, which provide a larger specific surface area for cell attachment and interconnected pathways that facilitate nutrient diffusion and sustained proliferation throughout the scaffold [222,248].

With regard to porosity, its effect on cell viability is not straightforward. For example, within the 60%, 72% and 76% porosity examined, there was no significant difference in cell viability in porous Ti-30Ta (Fig. 20b) [73]. A similar observation was recently reported by Liu et al. [119] in Voronoi tessellation-based Ti-50Ta lattices recorded at various fluorescence and quantitative assays (e.g., live/dead staining, CCK-8, ALP, ARS) after day 1, 4 and 7. It was found that the lattices with 30% and 39% porosities showed excellent cell viability (both at 150%), differentiation and mineralization (ALP to 133% and 142%; and ECM mineralization to 112% and 120%), outperforming the pure Ti control [119]. On the other hand, as the porosity of the Ti-50Ta lattice increased to 59%, the lattice exhibited very low cell viability (25% as shown in Table 9) [119]. Apart from porosity, the authors also attributed the better biological performance of lattices with 30%/39% porosities to their rougher surfaces compared to the 59% porosity, significantly enhancing cell adherence and proliferation.

Both the lattice topology and pore sizes directly affect the *in vitro* performance. Fig. 20a shows different cell proliferation results for Ti-25Ta lattices with different lattice topologies, all with a designed porosity of 60% [113]. As shown in Table 9 quantitatively, Schwarz primitive (Schwarz p.) topology achieved the highest cell proliferation at 119%, followed by diamond topology at 81%, while gyroid topology showed 71% proliferation relative to Ti control, tested with 3-day culturing of alveolar-bone-derived human teeth osteoblast cells [113]. Interestingly, the relationship between pore size and cell performance was not linear: the largest pore size (Schwarz p., 700 ± 16 μm) demonstrated the highest cell proliferation (119%), while the smaller pore sizes (Gyroid, 429 ± 15 μm) and (Diamond, 276 ± 17 μm) showed a similarly lower proliferation (71% and 81%, respectively). Although there is an unavoidable topology effect as discussed above, the results suggest that larger pore sizes within lattice structures may facilitate better nutrient transport and cell migration, promoting cell proliferation in this Ti-25Ta system. This trend is consistent with the observations in Ti-6Al-4V lattices. Wang et al. [249] investigated the *in vitro* responses of Ti-6Al-4V diamond lattices with a porosity of 86-94% (virtually equal porosity) and different pore sizes (800, 900 and 1000 μm). Indeed, the lattices with the largest pore size of 1000 μm show the best cell proliferation. Nevertheless, more systematic exploration (e.g., changing the pore size only with the same Ti-Ta alloy, lattice topology, and porosity) is still required to show the effect of pore size on the *in vitro* performance of Ti-Ta lattices.

#### 7.1.3 The effect of Ta content

When comparing Ti-Ta alloys to other Ti materials (e.g., pure Ti and Ti-6Al-4V), provided the same lattice topology and porosity level, Ti-Ta lattice exhibited comparable biological properties with slightly faster cell proliferation over time (Fig. 20c) [117]. Furthermore, pure Ta lattices show better cell proliferation than Ti-Ta lattices, as evidenced in Fig. 20d [119]. Both figures show the advantageous effect of the Ta element on the *in vitro* cell response in general.

However, among some studies of *in vitro* biological properties of Ti-Ta alloys with different Ta content, Ta content has little impact. Comparative *in vitro* assays have been conducted on dense and porous Ti-Ta alloys containing 10/25%Ta [46], 40/50%Ta [45], 10-70%Ta [[165], [250]], 25/65%Ta [244], 25-75%Ta [[105], [176]]**,** and 48.6-79.1%Ta [60]. No discernible and persistent differences in cell viability were detected between all the compositions after cell culturing up to 14 days (Table 9). For example, Ti-10Ta and Ti-25Ta with nonuniform porous structures (pore size: 200-300 μm) achieved comparable cell proliferation of 168% and 165% [46]. In addition, Wu et al. [151] demonstrated that varying phase volume fractions in two-phase α''+β Ti-25Ta had little difference in cell viability (osteosarcoma and fibroblast). These findings imply that the *in vitro* biological response of Ti-Ta alloys appears insensitive to their composition and phase constitution. A possible explanation is the static environment *in vitro*.

When cells are seeded on the Ti-Ta implant surface, a variety of events occur before measurable cell responses are observed [19]. The first event is the adsorption of water and the formation of hydroxyl groups (Ti-OH and Ta-OH) on the TiO_2_-Ta_2_O_5_ surface. It provides a properly hydrated environment for subsequent apatite deposition and protein adsorption. Not until the formation of the protein layer following protein adsorption would induce measurable cell responses, such as cell adhesion, proliferation, and differentiation, occur. Conventional *in vitro* systems are static, lacking a continuous supply of fresh water, medium, or proteins. Consequently, cell adhesion, proliferation, and differentiation occur at a slow pace. This can mask subtle differences arising from Ta content. We will discuss the influence of Ta content on the bioactive responses *in vivo* (a dynamic environment) in Section 7.2.2.

On the other hand, specific reports confirm improved biocompatibility of Ti-Ta alloy with Ta content under specific conditions. Fuerst et al. [121] reported that cell growth increases with Ta content. In their work, a higher Ta content resulted in wider deposited beads and a smaller pore size in the grid, providing more areas for cells to adhere and proliferate. Recent findings from Liu et al. [176] showed the same trend. Increasing Ta content of the scaffolds from Ti-25Ta to Ti-75Ta led to increases in MC3T3-E1 cell proliferation (102% to 110%), ALP activity (110% to 126%) and mineralization (107% to 192%), see Table 9. Notably, all Ti-Ta scaffold maintained haemolysis below 0.5%, indicating good hemocompatibility. The improved cell viability with increasing Ta content has also been reported by Zeng et al. [107,245,246]. Specifically, they reported an interesting composition-related observation in Ti-48.6Ta with an inhomogeneous composition: human osteoblastic cells were found to preferentially adhere to Ta-rich and transition regions over Ti-rich regions at the early incubation stage [196]. However, macrophages did not exhibit such a region-dependent tendency [196].

### 7.2 *In vivo* evaluation

The *in vivo* biological properties of Ti-Ta alloys within a living system, particularly under physiologically dynamic loading conditions, have been investigated in limited studies [46,69,86,105,119,176,223]. Alloy rods are implanted in the distal femur of rats or rabbits, and harvested after weeks of implantation to evaluate the osteointegration, bone regeneration and blood vessel ingrowth capabilities. The effects of alloy composition, porosity and surface features have been particularly addressed compared to control groups in these *in vivo* studies. Table 10 provides comprehensive quantitative data (e.g., bone-implant integration, bone volume metrics, and trabecular thickness), demonstrating consistently superior performance to Ti controls across multiple animal models and evaluation timepoints.

**Table 10:** *In vivo* biocompatibility of dense and porous Ti-Ta alloys with different porosity and surface features compared to their respective control.

Alloys (wt.%)	Fabrication technique	Porous structure	Surface characteristics^a^	Host	Implantation region	Bone-implant integration apposition		Bone volume metrics and comparative data		Trabecular thickness		Ref.
						Original value	%^f^	Original value	%^f^	Original value	%^f^	
Ti-48.6Ta	SPS	Dense	As-sintered	Sprague-Dawley rats	Distal femur	**-**		4.7^g^	470	2^m^	200	[69]
Ti∗								1	100	1	100	
Ti∗	PBF-LB/M	Porous scaffold, 80% porosity, 500 μm pore size	As-built	New Zealand white rabbits	Femoral condyle	**-**	**-**	16.5^h^	-	-	-	[105]
Ti-25Ta								20	-			
Ti-55Ta								37.5	-			
Ti-75Ta								24	-			
Ta								26	-			
Ti-25Ta	PBF-LB/M	Gyroid, 90% porosity, 700-900 μm pore size	As-built	New Zealand rabbits	Lateral condyle distal femur	28^c^	156	24^i^	185	0.4^n^	133	[86]
Ti∗						18	100	13	100	0.3	100	
Ti-25Ta	PBF-LB/M	Trabecular, 478 μm pore size	As-built			-		0.72^j^	124	-		[223]
Ti∗		Trabecular, 486 μm pore size				-		0.58	100	-		
Ti-50Ta	PBF-LB/M 100W	Voronoi tessellation trabecular	As-built			21^d^	66	-				[119]
	PBF-LB/M 220W		As-built			41	128					
	PBF-LB/M 340W		As-built			37	116					
Ti∗	PBF-LB/M		As-built			32	100	-				
Ti-25Ta	PBF-LB/M	BCC scaffold, 60% porosity, 321 μm pore size	As-built	New Zealand Rabbit	Femoral Condyle	37^d^	123	18^k^	106	-		[176]
Ti-50Ta		BCC scaffold, 60% porosity, 340 μm pore size				38	127	26	153	-		
Ti-75Ta		BCC scaffold, 60% porosity, 331 μm pore size				53	177	27	159	-		
Ti∗		BCC scaffold, 60% porosity, 302 μm pore size				30	100	17	100	-		
Ti-10Ta	DED-LB/M	200-300 μm non-uniform pores	As-built CA = 42 ± 2**°**^b^, 0.5-1.0 μL droplet	Sprague-Dawley rats	Bilateral distal femur	67^e^	203	-				[46]
Ti-25Ta			As-built CA = 38 ± 1**°**			48	145	-				
Ti∗			-			33	100	-				
Porous Ti-Ta alloys with surface modification												
Ti-10Ta	DED-LB/M + anodization	200-300 μm non-uniform pores	TiO_2_-Ta_2_O_5_ NTs 0.088 ± 0.005 μm nanotube diameter	Sprague-Dawley rats	Bilateral distal femur	58^e^	183	-				[46]
Ti-25Ta			TiO_2_-Ta_2_O_5_ NTs 0.067 ± 0.012 μm nanotube diameter			75	240	-				
Ti∗			TiO_2_ nanotubes			30	100	-				
Ti-10Ta	DED-LB/M + anodization		TiO_2_-Ta_2_O_5_ NTs 0.088 ± 0.005 μm nanotube diameter	New Zealand Rabbits		-		32^l^	107	38^o^	141	
Ti-25Ta			TiO_2_-Ta_2_O_5_ NTs 0.067 ± 0.012 μm nanotube diameter			-		68	227	72	267	
Ta	DED-LB/M		As-built			-		82	273	38	141	
Ti∗			As-built CA = 40 ± 1°^b^, 0.5-1.0 μL droplet			-		30	100	27	100	

∗Control material.

a Unless otherwise specified, reagent water was used under normal conditions (goniometer, macroscopic sessile-drop conditions, room temperature) for contact angle measurements.

b Dulbecco's Modified Eagle Medium (DMEM) as the test liquid under normal conditions (0.5-1.0 μL droplet).

c The percentage of the implant surface directly in contact with bone after 8 weeks.

d The percentage of the implant surface directly in contact with bone after 12 weeks.

e Osteoid to osteoblast ratio on the implant surface (%) represents the percentage of osteoid covered by osteoblasts on the implant surface after 5 weeks.

f Values normalised to their respective control and expressed as a percentage of control.

g Fold changes of percentage of bone volume total volume (BV/TV) compared to Ti control group after 10 weeks.

h Percentage of bone regeneration area measured using VG staining at week 12.

i Percentage of BV/TV after 8 weeks.

j Percentage of BV/TV after 12 weeks.

k Bone fraction BA/TA of new bone within the defect area after 12 weeks.

l The proportion of new bone deposited on the implant surface after 7 weeks.

m Trabecular thickness compared to Ti group after 10 weeks.

n Trabecular thickness measured in mm after 8 weeks.

o Trabecular bone area (%) within a 250 μm region of interest at the implant interface after 7 weeks.

#### 7.2.1 The effect of dense and porous structure

Experimental results indicate that dense or porous Ti-Ta alloys offer advantageous osteointegration and bone regeneration capabilities over pure Ti.

**Fig. 21:**
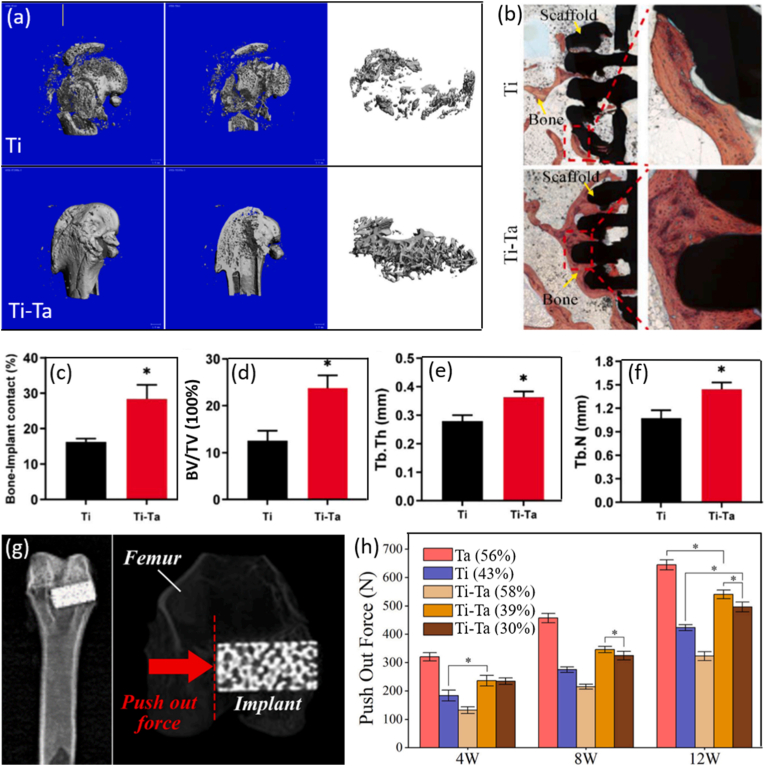
(a) Micro-CT images of dense pure Ti and Ti-48.6Ta implants in rats at week 10 of implantation [69]. Bone regeneration of porous pure Ti and Ti-25Ta scaffolds in rabbits at week 8 of implantation [86]: (b) histological image of explant sections after Van-Gieson staining, revealing the formation of new osseous tissue (in red) at the implant-host bone interface; (c) counting of the bone-implant contacts; quantitative analyses of the bone volume/tissue volume (BV/TV) percentage (d), trabecular thickness (Tb.Th) (e), and trabecular number (Tb.N) (f), with the assistance of micro-CT imaging. Push-out test of porous Ti-Ta implants in rabbits at week 4, 8 and 12 of implantation [119]: (g) micro-CT image and schematic diagram, and (h) push-out force comparison between Ta, Ti and Ti-15Ta samples (porosity is indicated in the bracket) at each time point.

Huang et al. [69] compared *in vivo* bone healing capability of dense pure Ti and Ti-48.6Ta. The former was commercially pure Ti (grade 2) while the latter was made using the SPS process, hot/cold working, annealing, and water quenching. Their rods (2 mm in diameter and 12 mm in length) were implanted transversely in the left hind-limbs of rats. X-ray examination showed that 2 out of 4 cases in Ti implantation had visible fracture lines at week 6 post-surgery and disengaged totally at week 10. In contrast, all 4 cases in Ti-Ta implantation had excessive calluses formed in the fracture area at week 6 and all fractured bones healed at week 10. This was further confirmed by micro-CT measurement shown in Fig. 21a, revealing the large filling of newly formed bone in Ti-48.6Ta in contrast with the partial filling of some tiny calluses in pure Ti at week 10 [69]. Quantitatively, the SPS-fabricated Ti-48.6Ta with as-sintered surfaces achieved 470% bone volume compared to Ti controls in rats, with trabecular thickness reaching 200% of Ti controls (Table 10) [69]. The authors ascribed the superior performance of Ti-Ta alloy to its lower elasticity (80.6 ± 0.9 GPa vs. 100-110 GPa in pure Ti), which is closer to that of human cortical bone (13.6-35.3 GPa [7,14]).

Most studies report an *in vivo* biological response of porous structured Ti-Ta alloys superior to that of pure Ti [86,105,119,176,223] and even comparable to that of pure Ta [105,119,176]. Zhao et al. [86]. They investigated *in vivo* performance of TPMS-based gyroid scaffolds of Ti-25Ta and pure Ti (designed porosity: 90%) fabricated by PBF-LB/M. The resultant Ti-25Ta gyroid scaffold had an actual porosity of 87.98%, an elastic modulus of 1.83 ± 0.1 GPa and a yield strength of 55.52 ± 2.1 MPa, essentially close to human cancellous bones (see Table 6). The pure Ti and Ti-Ta scaffolds were implanted perpendicularly in the femur of rabbits, harvested at week 8, mechanically prepared into thin explant sections, and stained with Van-Gieson staining. Fig. 21b shows the histological images. Clearly, more new osseous tissues formed around Ti-Ta implant. They readily filled the scaffold as compared to Ti implant, offering a higher percentage of bone-implant contact (Fig. 21c). Furthermore, quantitative analyses of micro-CT images indicated that Ti-Ta implant produced higher bone volume/tissue volume (Fig. 21d), trabecular thickness (Fig. 21e), and trabecular number (Fig. 21f) than pure Ti. Quantitatively, their Ti-25Ta gyroid scaffolds achieved 156% bone-implant contact, 185% bone volume/tissue volume, and 133% trabecular bone area compared to Ti controls (see Table 10) [86]. Similarly, Ti-25Ta trabecular structures implanted in the rabbit femur for 12 weeks demonstrated enhanced bone integration with 124% of the bone volume fraction compared to Ti controls (Table 10) [223]. These advantageous responses with porous Ti-Ta implants underlie their high push-out forces (Fig. 21g and h) [119], proving the outperforming osseointegration capability of Ti-Ta over pure Ti. At the same time, a porous structured implant can offer a favorable environment for bone regeneration in osteonecrosis [251]. As the bone tissue grows into the porous implant over time, the new bone and the implant form a composite which is expected to biomechanically differ from the original implant because of the decreased porosity and new constituent phase (bone) formation. Such variations are marginal early (e.g., within 12 weeks [252,253]) but substantial on a longer basis. For instance, Rubshtein et al. [254] reported that the bone-Ti implant composites measured elastic moduli respectively 21-34% at week 24 and 62-136% at week 52 higher than that of initial Ti implants. This increasing stiffness over time may induce the stress shielding and bone resorption during surgery recovery and implant servicing time, challenging the implant design.

#### 7.2.2 The effect of Ta content

**Fig. 22:**
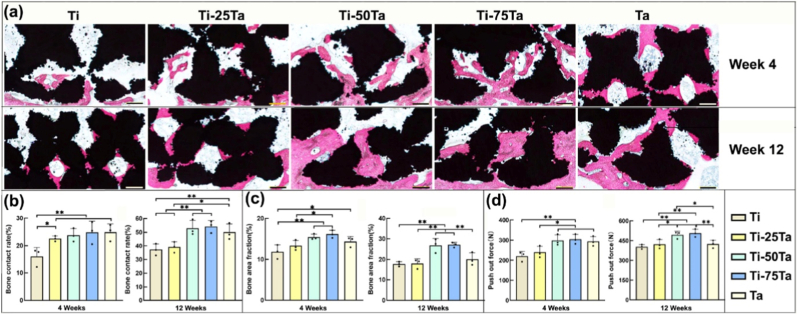
The effect of Ta content on the *in vivo* biological response of Ti-Ta lattices [176]: (a) histological examinations, stained with methylene and basic fuchsin (n = 3 rabbits); histomorphometric analyses of (b) bone contact rate, and (c) bone area fraction; (d) the average maximum push-out force of the various implants. Lattice implantation was carried out in rabbit femoral condyles and assessed at week 4 and 12, respectively. All lattices were printed by PBF-LB/M, exhibiting surface roughness of 7.0-12.8 μm, porosities of 61.84-65.87%, pore sizes of 299-340 μm and strut sizes of 403-435 μm. Scale bar = 200 μm.

**Fig. 23:**
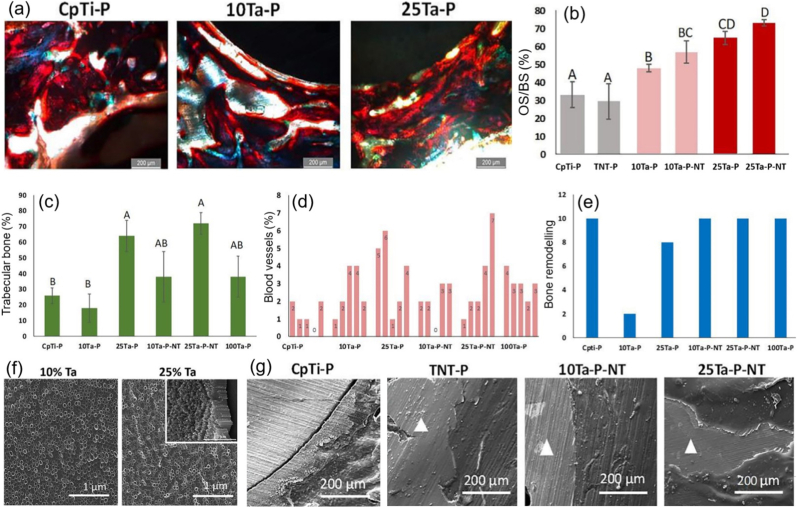
*In vivo* biological response of Ti-10Ta and Ti-25Ta implants as compared to pure Ti (CpTi) or TiO_2_ nanotubes (TNT) control [46]. (a) Histological micrographs of thin explant sections stained by Masson-Goldner's Trichrome. (b) Quantifications of the osteoid surface/bone surface (OS/BS) as a function of Ta content and coating treatment. The experiment was performed in a rat femur model and implants were retrieved at week 5. Similar work was conducted in a rabbit femur, and properties including the trabecular bone (c), blood vessel ingrowth (d), and bone remodelling (e), were quantitatively analysed at week 7. (f) SEM images showing the nanotube-coating Ti-Ta surface after electrochemical anodization. (g) SEM images of the implant-host bone interface after 5-week implantation. In the plots and images, letter 'P' denotes 'porous', and 'NT' denotes 'nanotube'.

Unlike the static *in vitro* assessment (Section 7.1), *in vivo* studies of Ti-Ta alloys have revealed a positive effect of Ta content on the biological properties under dynamic environment. Liu et al. [176] systematically evaluated the *in vivo* response of Ti-25Ta, Ti-50Ta and Ti-75Ta lattices implanted in rabbit femoral condyles. These lattices, made by PBF-LB/M, maintained comparable porosities (61.8-85.8%), pore size (320-340 μm), strut size (405-417 μm) and slight variations of surface roughness (7-9 μm), offering effective elastic moduli 1.07-2.26 GPa and compressive strengths 89.8-109.5 MPa. At week 4 of post-implantation, histological examinations showed that the lattices produced increasing new-bone fraction areas and bone contact rates with the increase of Ta content, Fig. 22a-c; at week 12, the lattices exhibited similar trends but the difference between Ti-50Ta and Ti-75Ta weakened. The increasingly enhanced bone ingrowth ability with Ta content was further justified with push-out test, which showed that Ti-50Ta and Ti-75Ta achieved high push-out loads (∼300 N at week 4 and ∼500 N at week 12), surpassing those of Ti-25Ta (∼240 N at week 4 and ∼420 N at week 12), Fig. 22d. Mitra et al. [46] carried out a similar comparison. Porous Ti-10Ta (10Ta-P) and Ti-25Ta (25Ta-P) (pore size: 200-300 μm; porosity: not presented), made by DED-LB/M, were implanted respectively in rat distal femur for 5 weeks and in rabbit femur for 7 weeks. Histological analyses revealed that Ti-25Ta was superior to Ti-10Ta in terms of osteoid formation at the implant-bone interface (Fig. 23a-b), the amount of woven bone within the medullary canal, trabecular bone formation (Fig. 23c), blood vessel ingrowth (Fig. 23d) and bone remodeling (Fig. 23e). Other studies also imply the positive effect of Ta content on the *in vivo* properties. For instance, Li et al. [105] observed a decreasing inflammatory response from as-built Ti-(25, 55, 75)Ta scaffolds (porosity: 80%; pore size: 500 μm; strut size: 200 μm) with increasing Ta content at week 4 and 12; the Ti-Ta scaffolds also exhibited greater bone regeneration than the Ti control (16.5%), respectively reaching 20%, 37.5% and 24% at week 12 (Table 10). For instance, as-built PBF-LB/M Ti-25Ta gyroid scaffolds achieved significant improvements with 156% bone-implant contact, 185% bone volume/tissue volume, and 133% trabecular bone area compared to its gyroid Ti controls at week 8 (Table 10) [86]. As-built PBF-LB/M Ti-50Ta with different processing parameters (100 W, 220 W, 340 W) showed significant 117%, 228%, and 206% bone-implant contact, respectively, compared to its Ti controls at week 12 (Table 10) [119], indicating the effect of Ta content regardless of process strategies and consequent surface features. However, porous Ti-Ta may perform worse than pure Ti if inappropriate printing has led to numerous unmelted Ta particles in the microstructure which act as defects rather than contributing to the alloy's desired properties [119]. It is worth noting that Ti-75Ta lattices behaved essentially comparable to Ti-50Ta in Liu et al.’s study [176] and Ti-75Ta scaffolds were inferior to Ti-55Ta in Li et al.’s work [105], suggesting that increasing Ta content around 50% appears to be optimum.

We propose that there are two reasons for the much more substantial effect of Ta *in vivo*.

• The dynamic *in vivo* environment. In contrast to static *in vitro* conditions (7.1.3), *in vivo* responses are monitored in a dynamic environment (e.g., blood flow, interstitial fluid circulation, continuous molecular turnover). It continuously renews the fluid-surface interface, enabling continuous cell-Ti-Ta surface interactions. This amplifies the effect of Ta content, or the more biocompatible Ta_2_O_5_ in Ti-Ta alloys, making the differences in cell recruitment and bone formation more evident.
• Mechanical property (e.g., modulus) compatibility *in vivo.* In both *in vivo* studies regarding the effect of Ta content, the elastic modulus of Ti-Ta decreases with Ta content. For instance, the effective elastic modulus of Ti-Ta lattices decreased from 2.26 GPa to 1.31 GPa as Ta content increased from 25% to 75% [176], closer to that of cancellous bones (0.02-0.83 GPa). Under physiological loading conditions (walking, chewing), this mechanical compatibility enhances interfacial stress transfer, thereby improving mechanotransduction [86,119] and promoting bone formation and tissue regeneration *in vivo* [176,244]. This mechanism, however, is absent in conventional *in vitro* systems. As a result, the mechanical benefit of higher Ta content appears only in animal models, undiscovered in short-term cell assays (Section 7.1.3).

### 7.3 The effect of surface modification

As discussed in Sections 5, 6, 7.1, surface modification of Ti-Ta alloys has been intensively pursued to improve their wettability and corresponding biological performance in the physiological environment [109,255].

*In vitro* studies confirm that surface modifications consistently enhance the biological performance compared to unmodified surfaces, as detailed below.

• **Cell responses.** MAO treatment (forming adherent TiO_2_-Ta_2_O_5_ surface layer and creating nanopores) on Ti-48.6Ta substantially roughened the alloy's surface from as-polished R_a_ = 261 ± 11 nm to R_a_ = 882 ± 54 nm treated at 250 V or R_a_ = 854 ± 29 nm treated at 300 V. As evident in Table 8, Table 9, Table 10, the resulting alloy offered a superhydrophilic surface (CA = 0°) and expectedly improved biological responses: a relative cell proliferation of 123%, ALP of 129% and ECM mineralization of 133% to its control when MAO treated at 300 V [196]. Roughening Ti-Ta alloy surface by ultrasonic cavitation and SiO_2_ microparticle abrasion enhanced its biocompatibility to osteoblast cells (MC3T3-E1): the cell absorbance increasing with the diameter of SiO_2_ abrasive balls and SiO_2_ mass fraction in the solution [221]. For instance, the treatment using 10 μm SiO_2_ particles created a crater volume 60% higher than that from 1 μm SiO_2_ particles and accordingly a cell absorbance of 50% higher after 72 h incubation; increasing SiO_2_ mass fraction from 2 wt% to 6 wt% to 8 wt% increased the cell absorbance by 7.4% and 15.6%, respectively. These findings suggest surface modification can significantly enhance cell responses [46,85,119].
• **Apatite formation.** As mentioned in 7.1.1, the apatite formation layer enhances bone regeneration and healing. The surface modification further enhances the apatite formation capability of Ti-Ta alloys. For example, immersing anodized Ti-93.8Ta in SBF for 28 days produced a Ca/P ratio of 1.38, which is higher than that of non-anodized counterpart (1.19) and closer to the standard value (1.67), indicating an improved apatite formation ability [195]. Furthermore, hydrothermal-precalcification treatment can dramatically enhance the apatite-forming ability of Ti-Ta [209]: a Ti-60Ta plate had a rough hierarchical micro-/nano-structure on TiO_2_-Ta_2_O_5_ layers full of Ca^2+^ and PH $O_{4}^{2-}$ ions after the treatment. These ions acted as nuclei for apatite growth and promoted apatite formation within 1 day of immersion in SBF [209].

After anodization treatment, the *in vivo* studies of the Ti-(10, 25)Ta alloys demonstrate improved bone-implant interactions (as summarized in Table 10). A typical study was done by Mitra et al. [46]. They presented their comprehensive *in vivo* study of Ti-Ta alloys coated with oxide nanotubes (NT) in rat and rabbit femur models. They utilised anodization to create a hydrophilic surface layer with densely TiO_2_-Ta_2_O_5_ arrayed nanotubes on Ti-10 and 25Ta, Fig. 23f. In a rat mode at week 5 post-surgery, the anodized Ti-Ta implants exhibited a higher percentage of osteoid surface per bone surface than the TiO_2_ nanotubes (TNT) control (Fig. 23b). These nanotubes formation directly resulted in superior biological performance, with osteoid to osteoblast ratio of Ti-10Ta reaching 183% and Ti-25Ta achieving 240% compared to TNT controls (refer to Table 10). The same advantage was achieved in a rabbit model at week 7, with higher trabecular bone formation to 267% and 141% on anodized Ti-10Ta and Ti-25Ta, respectively, relative to its control Ti, including improved blood vessel and bone remodeling (Fig. 23c-e). Furthermore, regions with overlapping implant and bone tissues were formed at their interface in contrast to a noticeable gap retained in pure Ti control (Fig. 23g), demonstrating the potent bonding between the coated Ti-Ta implant and surrounding tissues. This study appears to be the only one *in vivo* comparing anodized Ti–Ta with TNT (TiO_2_ nanotube) surfaces. Their findings show that nanotube coating combined with a higher Ta content provides porous Ti-25Ta with the optimal biological properties, highlighting the significance of surface modification and the Ta content in the parent Ti-Ta alloys.

## 8 Conclusions and outlooks

### 8.1 Conclusions

This paper has reviewed the major characteristics of biomedical Ti-Ta alloys for orthopedic implant applications. These include the manufacturing and surface modification of Ti-Ta alloys, their phase transformation, microstructure, mechanical properties and *in vitro* and *in vivo* biological responses. Below is a summary of the main observations in Ti-Ta alloys:

1) Various manufacturing methods have been used to manufacture Ti-Ta alloys (bulk and porous). Conventional approaches such as melting and powder metallurgy account for ∼70%. Over the last decades, additive manufacturing of Ti-Ta alloys has attracted growing interest (∼27%) because of its manufacturability of complex geometries and topologies for patient-specific orthopedic implant applications.
2) Ti-Ta alloys' phase constitution and microstructure mainly rely on the cooling rate from β-transus temperature and alloy composition, regardless of the manufacturing method and the geometry of the alloy (bulk or porous). The Ti-Ta alloys consist of stable phases, such as α, β or their combinations after slow cooling (cooling rates <10^−1^ °C/s, typically furnace cooling), and of metastable phases, such as α′, α'', ω, β or their combinations, after moderate (cooling rate 10°-10^1^ °C/s, typically air cooling) or rapid (cooling rate >10^2^ °C/s, typically water quenching and AM) cooling. The Ti-Ta bulk alloys with metastable phases show slightly lower tensile strength (700-1100 MPa) and higher ductility (>10%) than those with stable phases (700-1200 MPa, <10%).
3) Dense and metastable Ti-Ta alloys exhibit a minimum elastic modulus of about 60 GPa at 25%Ta and 70%Ta, respectively, the starting point for further mimicking the mechanical properties of natural bone. Specifically, Ti-25Ta stochastic or strut-based diamond lattices at (80-90)% porosity enabled by AM exhibit elastic moduli and compressive strengths very close to those of cancellous tibia/femur bones. Furthermore, Ti-25Ta in a triply periodic minimal surface-based diamond lattice at 30% porosity essentially bio-mimics cortical bones.
4) As compared to pure Ti and Ti-6Al-4V in clinical surgeries for decades, Ti-Ta alloys offer comparable or superior properties, including corrosion resistance in a physiological environment, *in vitro* responses (e.g., cell adhesion, cell viability, cell proliferation and osteogenesis) and *in vivo* responses (e.g., osseointegration, bone volume and trabecular formation).
5) The Ta content in Ti-Ta alloys generally has little effect on short-term *in vitro* viability. However, increasing Ta content enhances *in vivo* bone regeneration, vascularization, remodeling and reduces inflammation, within a Ta range between 10% and 75% examined though benefits beyond 50%Ta weaken. We proposed that the static *in vitro* and dynamic *in vivo* conditions are the primary reasons for the substantially different influences of Ta content. Mechanical compatibility also plays a role. Porous Ti-Ta shows weaker early adhesion but superior long-term proliferation and mineralization, depending on porosity, surface roughness, and topology, compared to dense Ti-Ta. Surface nanofeatures improve both the *in vitro* (e.g., wettability, osteogenic activity, and apatite formation) and *in vivo* (bone bonding, trabecular growth, vascularization, and remodeling) responses.

### 8.2 Outlooks

While tremendous efforts have been dedicated to understanding and exploring the potentials of Ti-Ta alloys for orthopedic implant applications, there are still challenges, and a lack of understanding remains regarding important mechanical and biological properties crucial to their successful clinical applications. These underexplored properties and the associated research opportunities are described below.

1) Ti-Ta lattices do not concurrently match the static and dynamic mechanical properties of natural bones

Highly porous Ti-30Ta stochastic lattices (porosity: 89-92%) [114], for instance, can closely match the elastic moduli, strength, ductility and elastic admissible strain of cancellous tibia/femur bones [14], but, their high-cycle fatigue strength (1.3-2.6 MPa at 10^6^ cycles) remains orders of magnitude lower than that of cancellous tibia bones (∼100 MPa) [182]. Conversely, Ti-50Ta Voronoi tessellation-based lattices can meet fatigue demands but exhibits stiffness (2.3-7.2 GPa) inferior to that of cortical bones (15.4-35.3 GPa) [14,119,181]. Reducing porosity increases fatigue strength despite a concurrent increase in elastic modulus [14,119], which is applicable to the development of cortical bone replacements. In the case of cancellous bones, however, it is rather formidable to develop suitable Ti-Ta lattices offering a satisfactory combination of static and dynamic mechanical properties. Overcoming this intrinsic trade-off will require coordinated advances in topology optimization (e.g., triply periodic minimal surface-based unit cells to reduce stress concentration at the node [256]), surface engineering to mitigate surface roughness [257], and the use of pre-alloyed powder to control internal flaws [258]. Together, these strategies offer a pathway toward Ti-Ta lattices that may simultaneously satisfy the static and dynamic demands of cancellous bone.

2) Many clinically relevant properties of Ti-Ta alloys remaining underexplored or absent
  • **Tensile properties.** Clinical observations indicate that cortical bone fractures in most cases are caused by tensile stress and strain, while bones are subjected to bending or torsion [259]. To the best of our knowledge, there was only one study reporting the tensile responses of Ti-Ta lattice structures [118], where Ti-Ta lattices present unsatisfying ductility, e.g., 3.54 ± 0.04% in Ti-25Ta at 55% porosity and 5.89 ± 3.35% in Ti-65Ta at 58% porosity (both in F_2_CC_z_ unit cell) [118]. The lack of tensile property data imposes uncertainty for Ti-Ta lattices' applications to tibia/femur bone fixation.
  • **Wear resistance.** Wear resistance is particularly critical to the durability and longevity of implants subjected to cyclic loadings, such as those used in dental and joint replacements. It is another underexplored property of Ti-Ta alloys [193]. The intrinsic TiO_2_-Ta_2_O_5_ layer (<10 nm thick) [233] on the surface of the Ti-Ta alloys is insufficient to protect the implant from fretting wear. As a result, surface modification treatments are necessary, such as coating and oxidation.
  • **Corrosion resistance under inflammatory conditions.** The inflammatory environment (pH = 5.0-6.0) around implants creates a high risk of implant loosening due to individual immune responses and ion-induced cytotoxicity arising from wear particles and corrosion products [241,242]. In Ti-Ta alloys, ion release can originate from particle debris and partial dissolution of the protective oxide passive film. This acidic environment can also be associated with fluoride exposure, particularly in dental settings, which may further accelerate the film dissolution and increase ion release, potentially contributing to cytotoxicity and tissue damage [260,261]. Surface treatment may be an applicable approach to enhance the stability of the oxide film and further studies are required to understand the corrosion behaviors of Ti-Ta alloys and optimize their compositions under these environments.
  • **Dynamic wettability.** After implantation, the initial bone-implant interaction is water adsorption, where physiological fluids are continuously mobile. The extensive static wettability dataset in literature is far from revealing such dynamic and time-dependent wetting behavior. Dynamic wettability measurements [262] can capture this liquid motion, including spreading kinetics on dense (solid) surfaces, and capillary rise (infiltration) of Ti-Ta implant materials. Such data, which is currently absent, will provide valuable input to understanding and optimizing the bone-Ti-Ta implant interactions, especially at the initial stage.
  • **Long-term *in vivo* performance.** All *in vivo* studies of Ti-Ta alloys so far have been restrained to a time span of maximum 12 weeks [105,119,176,223], focusing on the bone ingrowth and integration with the implant and secondarily examining the mineralization, vascularization and bone remodeling. With the further passage of time, bone regeneration continues and fills the implant pores, reshaping the implant toward a bone tissue-implant composite. This dynamic bone-implant integration over time entails changes in the implant network structure (e.g., permeability) and mechanical properties (particularly stiffness) [253,254]. Furthermore, potential complications may arise unexpectedly from long-term post-implantation, including undermining osseointegration [263], loosening [264], and chronic inflammation [240]. To ensure Ti-Ta implant perform desirably and progress for clinical trials, it is essential to fully understand the implant structure evolution and property variation with the implant timeline through *in vivo* examination and monitoring on a regular basis.
3) Potential applications of Ti-Ta alloys in other areas
  • **Drug-eluting porous Ti-Ta implants.** Effective drug-eluting orthopedics must ensure compatibility with therapeutic agents and sustained release delivery [265]. Current drug-releasing orthopedic medical devices (e.g., polymer coatings or porous ceramics) face issues such as burst release [266], limited drug storage capacity, instability [267], and mechanical incompatibility with metallic implants [268]. Porous Ti-Ta may help overcome these challenges. The biocompatible TiO_2_-Ta_2_O_5_ oxide may improve surface anchoring for drug carrier layers, the micro-interlocking structures that secure therapeutic agents, preserve drug integrity, and mitigate burst release. A porous Ti-Ta implant can avoid the mechanical incompatibility issue while its interconnected pore network offers a substantial surface area for drug storage. On top of effective drug-releasing functionality, drug-eluting porous Ti-Ta implants can enhance bone healing from the porous architecture [268], and may passively reduce adverse host response from the stable protective TiO_2_-Ta_2_O_5_ surface. There are some investigations in other porous alloys (e.g., NiTi and Ti-6Al-4V) which show promising performances [269,270]. Future work should systematically evaluate drug loading capacity, release kinetics, and biological outcomes using porous Ti-Ta alloy designs.
  • **Ti-Ta for antibacterial functions** Post-surgical infection remains a major cause of implant failure because bacteria can adhere and form biofilms before host cells occupy the surface [271,272]. Although Ti-Ta surfaces exhibit excellent cytocompatibility due to their oxide chemistry, their native TiO_2_ and Ta_2_O_5_ possess limited intrinsic antibacterial activity [217,273]. To deliver sustained protection without compromising the biocompatibility of Ti-Ta alloys, one emerging approach is to use fluoridated TaO_x_ coatings (F-TaO_x_) on Ti-Ta, which can acidify and inhibit bacterial cells while maintaining fluoride levels below cytotoxic thresholds for host cells [274]. Another promising approach is hierarchical micro-/nano-structure engineering on the Ti-Ta surface that shows antibacterial effects to some extent [275]. In addition, nano-structure surface features have been reported to physically disrupt bacterial membranes via piercing while simultaneously supporting bone cell adhesion and osseointegration [276]. Last, introducing antibacterial elements, such as trace Cu [277], is always a simple and effective solution. However, fully examining their effects on other properties is essential in a bid to achieve a balanced comprehensive performance.

## CRediT authorship contribution statement

**S.D. Luo:** Writing – original draft, Conceptualization. **S. Paisansuthichol:** Writing – original draft, Visualization. **N. Tran:** Writing – review & editing, Supervision. **M. Qian:** Writing – review & editing, Supervision. **T. Song:** Writing – review & editing, Supervision, Project administration, Funding acquisition, Conceptualization.

## Ethics approval and consent to participate

There is no content in this review manuscript that relates to ethical approval and consent to participate.

## Declaration of competing interest

The authors declare that they have no known competing financial interests or personal relationships that could have appeared to influence the work reported in this paper.
